# Photocatalytic Hydroalkylation of Aryl-Alkenes

**DOI:** 10.1021/acs.joc.2c01304

**Published:** 2022-08-01

**Authors:** Cornelia S. Buettner, Michael Schnürch, Katharina Bica-Schröder

**Affiliations:** Institute for Applied Synthetic Chemistry, TU Wien, Getreidemarkt 9/163, 1060 Vienna, Austria

## Abstract

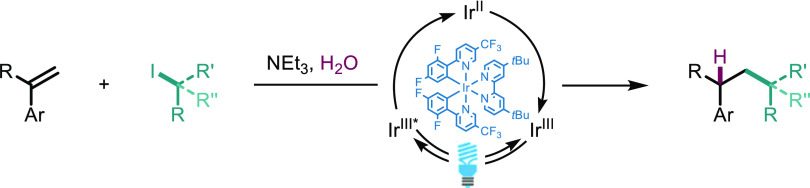

Here, we present a visible light-catalyzed hydroalkylation of aryl-alkenes affording C–C bonds using aryl-alkenes and alkyl iodides. We demonstrate the formation of various hydroalkylation products in excellent yields, with primary, secondary, and tertiary alkyl iodides being tolerated in the reaction. Mechanistic experiments reveal a pathway consisting of halogen atom transfer followed by a radical-polar crossover mechanism delivering the desired hydroalkylation products.

## Introduction

Photoredox catalysis has been utilized as a powerful tool in synthesis, rejuvenating the field of radical chemistry for development of modern organic transformations.^1−4^ In comparison to polar processes, radical reactions offer orthogonal reactivity that allows construction of organic scaffolds through non-traditional disconnections. Further, the use of visible light has led to a renaissance of radical chemistry.^5−9^

**Scheme 1:**
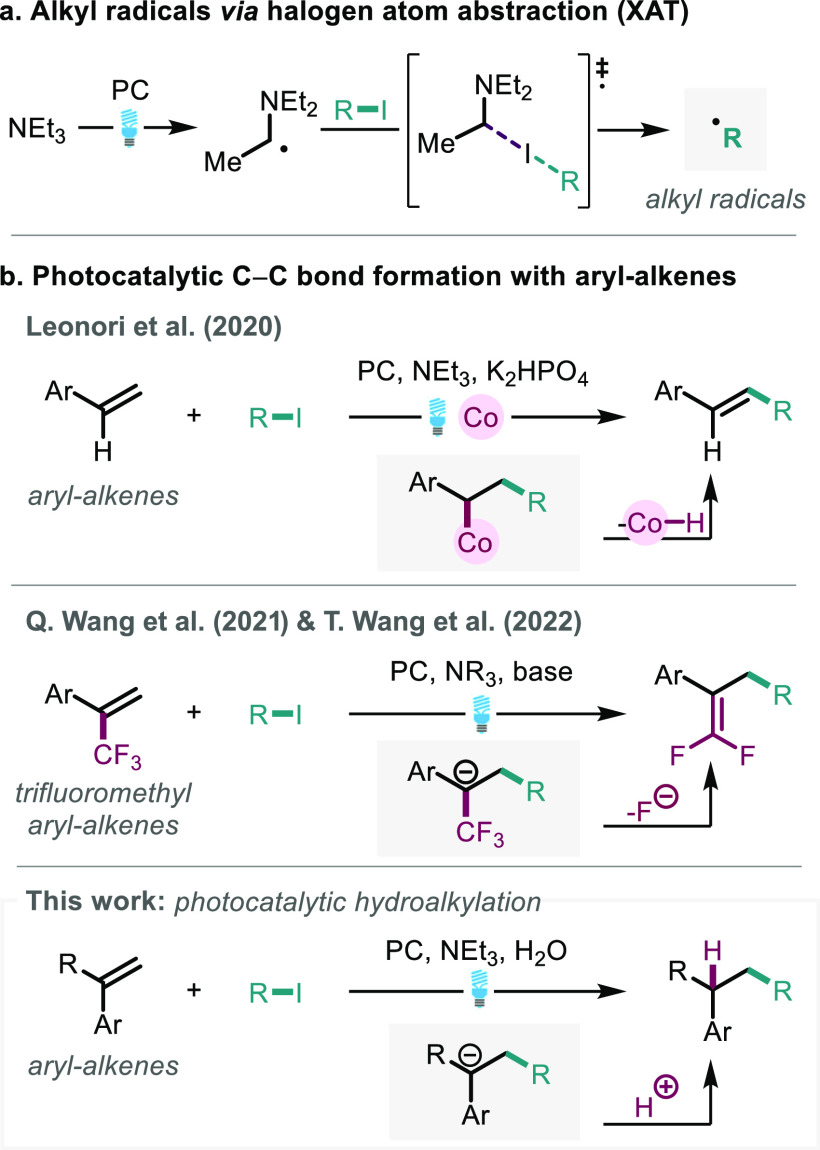
(a) Formation of Alkyl Radicals from Alkyl Iodides Using Triethylamine as a Halogen Atom Transfer (XAT) Reagent. (b) Examples of Photocatalytic Addition of Alkyl Radicals (from Alkyl Iodides) to Aryl-Alkenes. PC = Photocatalyst

Halogen atom transfer (XAT) is a key step for many radical reactions, furnishing radical species from alkyl/aryl halides. Traditionally, the use of tributyl tin as a halogen abstraction reagent has been heavily relied on for radical transformations.^10^ More recently, the uses of silicon, germanium, boron, phosphorus, and carbon radicals have established themselves as less toxic alternatives.^11^ Alkyl amines such as triethylamine are powerful XAT reagents undergoing facile oxidation in the presence of photocatalysts.^11,12^ Formation of the nitrogen-centered radical cation leads to a significant decrease in *p*Ka of the α-C–H bond (>40 to 14.7 in MeCN); loss of a proton furnishes the α-amino radical.^13^ Early work by Meyer et al. employed amines as reductive quenchers for ruthenium photocatalysts.^14,15^ Work by Yoon et al. utilized this approach to develop photocatalytic radical reactions.^16−19^ More recently, tertiary alkyl amines have been used for XAT with alkyl iodides, where the α-amino radical has been shown to undergo halogen atom abstraction *via* a co-linear and bimolecular transition state (Scheme 1a):^11^ this has been shown to be very fast for alkyl iodides.^20^ Furthermore, this method of radical formation negates the need for catalysts with reduction potentials comparable to those of alkyl iodides (less than −2 V vs SCE).^21,22^

The regioselectivity of radical addition to alkenes is generally dictated by the stability of the resulting radical. For example, the addition onto aryl-alkenes results in a stabilized benzylic radical.^23^ There are several methods to turnover such radical intermediates to give synthetically useful transformations; the alkylation of aryl-alkenes with alkyl iodides has been achieved using two methods (Scheme 1b). Leonori et al. reported the formal Heck reaction utilizing cobalt (and later copper) complexes to trap the benzylic radical and returning the derivatized alkene through β-hydride elimination.^24,25^ Wang et al. exploited pendant α-trifluoromethyl groups to afford the difluoroalkenes after elimination of fluoride.^26,27^ Here, we present the development of a photocatalytic hydroalkylation reaction without the requirement for additional transition metal complexes or strategically placed leaving groups, allowing access to hydroalkylation products catalyzed by visible light.^28^ Thus, we became interested in developing a reaction platform allowing for the photocatalytic, radical C–C bond formation between aryl-alkenes and alkyl iodides.

## Results and Discussion

Our initial investigations focused on representative hydroalkylation of olefin **1a** with 2-iodopropane **2a**, affording the C–C bond product **3a** (Table 1). Optimization studies (developed from the initial hit, entry 1) rapidly afforded conditions providing hydroalkylation product **3a** in excellent yield (entry 2). A screen of commonly used photocatalysts showed that the iridium photocatalyst (Ir[dF(CF_3_)ppy]_2_(dtbpy))PF_6_ (Ir-1)^29^ gave the highest conversion to desired product **3a**. The organic photocatalyst 4CzIPN^30^ (entry 3) afforded very low conversion to the desired C–C bond product **3a**. Likewise, popular iridium photocatalysts: Ir(ppy)_3_ (entry 4), [Ir(dtbbpy)(ppy)_2_]PF_6_ (entry 5), and [Ir(*p*F(*Me*)ppy)_2_(dtbbpy)]PF_6_ (entry 6) could not outperform Ir-1. Control reactions showed the requirement of water (entry 7), likely as a proton source (*vide infra*). Similarly, omission of irradiation or photocatalyst afforded no product **3a** (entries 8 and 9), indicative that the photocatalyst excited state is involved in the reaction. Finally, the reaction run under air (entry 10) ascertains quenching of the photocatalyst by singlet oxygen. With the optimized conditions in hand, we became interested in examining the scope for the hydroalkylation reaction and investigating the mechanism of the transformation.

**Table 1:**
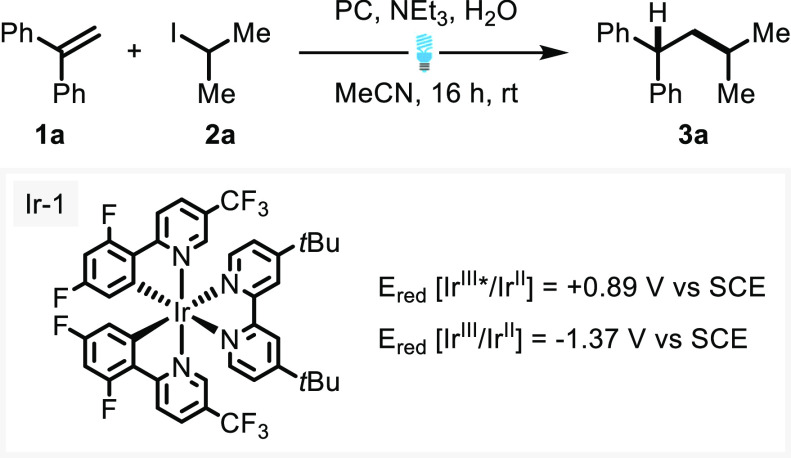
Reaction Optimization for Photocatalytic Hydroalkylation

entry	variation of conditions	yield of **3a**
1^a^	CH_2_Cl_2_	21%^b^
2		85%
3	4CzIPN	44%^b^
4	Ir(ppy)_3_	48%^b^
5	[Ir(dtbbpy)(ppy)_2_]PF_6_	54%^b^
6	[Ir(*p*F(*Me*)ppy)_2_(dtbbpy)]PF_6_	52%^b^
7	without H_2_O	45%^b^
8	no light	0%^b^
9	no PC	0%^b^
10	run under air	38%^b^

a **1a** (1 equiv), **2a** (2 equiv), NEt_3_ (4 equiv), Ir-1 = (Ir[dF(CF_3_)Ppy]_2_(dtbpy))PF_6_^31^ (1 mol %), H_2_O (10 equiv), 0.1 M MeCN, blue LED (40 W), FPT, argon atmosphere using cyclohexyl iodide affording **3b**.

b NMR yields determined with 1,1,2,2-tetrachloroethane as the internal standard.

**Scheme 2:**
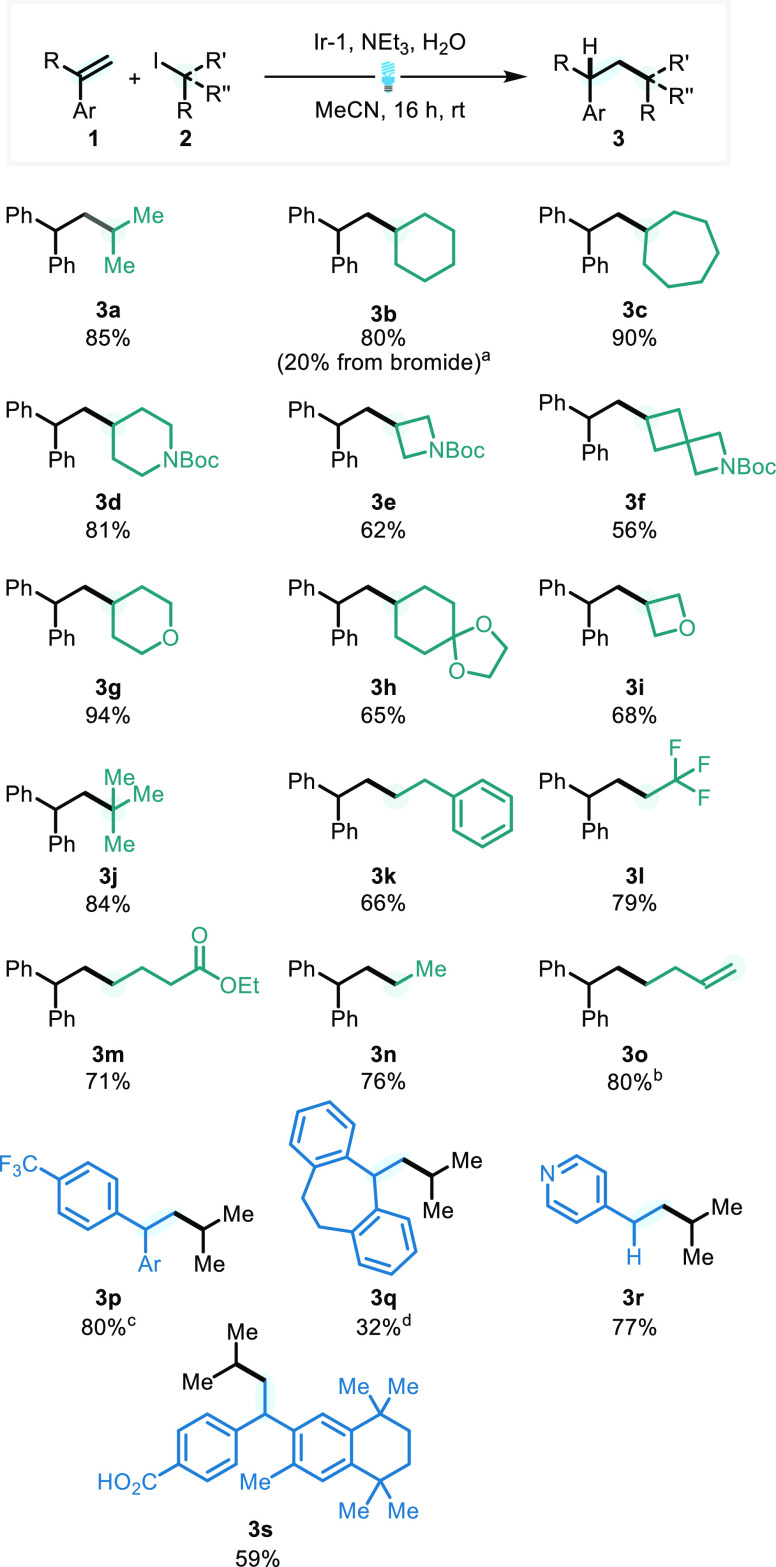
Hydroalkylation Substrate Scope Alkene (1 equiv), alkyl iodide (2 equiv), NEt_3_ (4 equiv), Ir-1 = (Ir[dF(CF_3_)ppy]_2_(dtbpy))PF_6_ (1 mol %), H_2_O (10 equiv), 0.1 M MeCN, blue LED (40 W), FPT, argon atmosphere. Yield determined by NMR with 1,1,2,2-tetrachloroethane as the internal standard. Using cyclopropylmethyl iodide. Ar = 4-CF_3_-C_6_H_4_. Difunctionalized product observed.

**Scheme 3:**
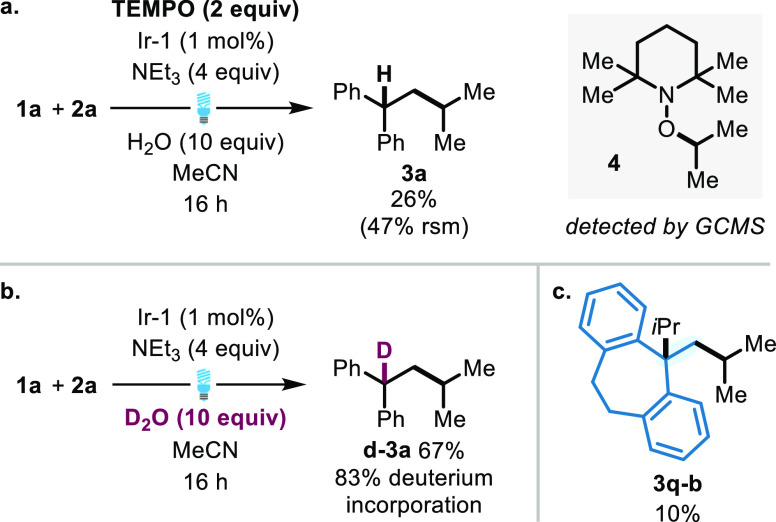
Control Experiments (a) Radical trap experiment using TEMPO. (b) Deuteration experiment. (c) Structure of di-alkylation product **3q**-**b**. TEMPO = 2,2,6,6-tetramethylpiperidinyloxyl; Ir-1 = (Ir[dF(CF_3_)ppy]_2_(dtbpy))PF_6_.

The simple alkyl iodide **2a** used for optimization studies afforded excellent yields of the hydroalkylation product **3a** (Scheme 2). Similarly, excellent yields were observed for other secondary alkyl iodides affording the cyclohexyl and cycloheptyl products **3b** and **3c**, respectively. Interestingly, the use of cyclohexyl bromide provided the desired product **3b** in only 20% yield. This is consistent with the higher BDE values for C–Br than the corresponding C–I compounds, resulting in slower rates of XAT.^11^ Protected nitrogen could be incorporated to afford protected piperidine **3d**, azetidine **3e**, and spirocyclic amine **3f**. Likewise, pyran and oxetane moieties were tolerated providing very good yields of the oxygen containing products **3g** and **3i**. The ketal gave good yields of the corresponding hydroalkylation product **3h**. *tert*-Butyl iodide underwent efficient hydroalkylation with alkene **1a** to provide a quaternary carbon center on *tert*-butyl product **3j** in excellent yield. The developed conditions equally allowed the use of primary alkyl iodide substrates, giving very good to excellent yields. Phenyl-bearing iodide gave aromatic compound **3k**, while 2,2,2-trifluoroethyl iodide gave fluorinated product **3l**. Primary alkyl iodides bearing ester functionality also underwent photocatalytic hydroalkylation to ester **3m** in very good yield. Surprisingly, iodoethane provided hydrocarbon **3n** in very good yields, suggesting the efficient interception of the proposed primary radical formed through XAT. The use of cyclopropylmethyl iodide provided olefin-bearing hydroalkylation product **3o** in excellent yield and provided evidence for a radical pathway, where the formation of the radical is concomitant with cyclopropyl ring-opening, typically observed for radical processes.^32,33^ Electron withdrawing groups on the alkene reaction partner, such as trifluoromethyl, allowed for excellent hydroalkylation providing fluorinated product **3p**. Hydroalkylated product **3q** was only isolated in modest yields. Isolation of di-alkylation product **3q**-**b** (Scheme 3c) inferred persistence of an intermediate that could be intercepted by radical–radical recombination to afford the observed, doubly alkylated side product. The significantly more impressive yield of **3r** from 4-vinylpyridine highlights the tolerance of the reaction toward aryl-alkenes bearing single aromatic moieties. Finally, this methodology was used to demonstrate a late-stage derivatization of pharmaceutical bexarotene, which provided the alkylated product **3s** in good yield.^34^

Next, we aimed to probe the mechanism of our C–C bond formation that gave rise to the hydroalkylation products **3a**-**3s**. The addition of radical scavenger TEMPO to the optimized reaction conditions between alkene **1a** and alkyl iodide **2a** provided a significantly reduced yield of **3a** (26%) and returned 47% of starting material **1a**. The remaining starting material, not typically observed without TEMPO addition, and more significantly detection of TEMPO-adduct **4** showed a radical process to be operative (Scheme 3a). Additionally, **4** establishes that the formation of the radical occurs from the alkyl iodide, which undergoes addition to the olefin **1a**.^35^ To probe the C–H bond formation, we studied the deuterium incorporation of **3a** (forming **d**-**3a**; Scheme 3b). Replacement of water with deuterium oxide in the reaction between **1a** and **2a** afforded 83% deuterium incorporation in the deuterium-alkylation product **d**-**3a**, clearly suggesting the source of the hydrogen to be from the added water. We have similarly observed that the addition of water is concomitant with an increase in yield as demonstrated in our optimization studies (*vide supra*). In isolation, C–H bond formation is able to proceed through a radical or ionic process. There is no evidence for the hydrogen atom transfer (HAT) to stabilize benzyl radicals (BDE_H-Bn_ = 85 kcalmol^–1^)^36^ from water (BDE_H-OH_ = 119 kcal mol^–1^);^36^ we expect this to be energetically unfavorable and thus unlikely.^37^ This suggests a polar process to be dominant for C–H bond formation.

**Figure 1:**
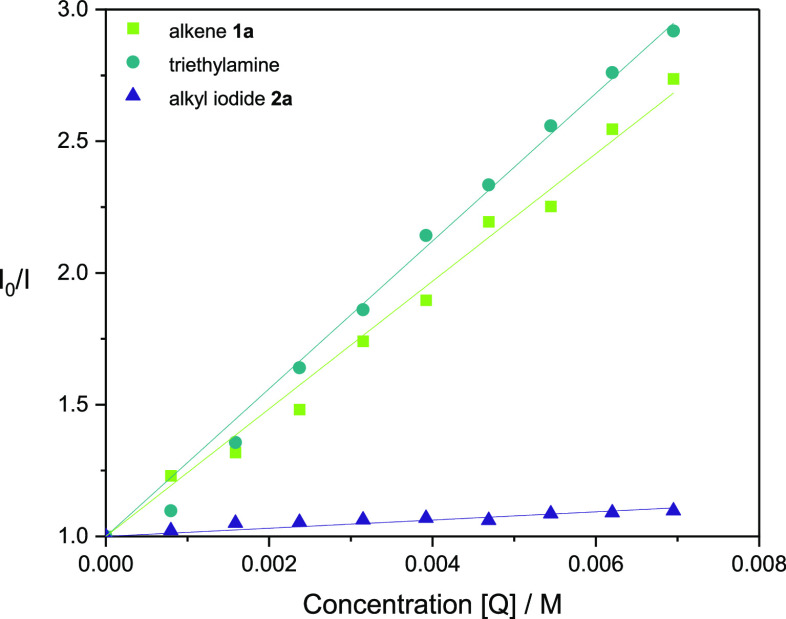
Stern–Volmer fluorescence quenching studies with triethylamine, alkene **1a**, and alkyl iodide **2a**.

We conducted Stern–Volmer quenching studies to investigate fluorescence quenching of the excited state iridium photocatalyst Ir-1 by triethylamine, alkene **1a**, and alkyl iodide **2a** (Figure 1). Quenching of the excited state photocatalyst by triethylamine was observed, likely proceeding through reductive quenching of the iridium photocatalyst (*E*_red_ [Ir^III*^/Ir^II^] = +0.89 V vs SCE)^29^ concomitant with single-electron transfer (SET) from the alkyl amine (*E*_red_ [Et_3_N^·^^+^/Et_3_N] = +0.83 V vs SCE).^21^ The quenching constant (*k*_q_) for triethylamine was determined using the Stern–Volmer relationship and the lifetime of the photocatalyst (τ_0_).^29^ It was calculated to be of the order of 10^8^ M^–1^ s^–1^ (Supporting Information), which is comparable to literature values.^38^

Additionally, fluorescence quenching of the photocatalyst by the alkene **1a** was observed in similar intensity to that of the amine. Electron transfer processes between alkenes and excited-state photocatalysts have been predominantly reported *via* reductive quenching of the photocatalyst yielding the corresponding radical cation of the alkene. Literature reports for photocatalytic oxidation of **1a** (*E*_red_ [**1a**^·^^+^/**1a**] = +1.54 V vs SCE)^21^ have predominantly utilized the highly oxidizing photocatalyst Mes-Acridine (*E*_red_ [PC*/PC^–^] = +2.08 V vs SCE).^31,39,40^ The significantly lower reduction potential of iridium complex Ir-1 (*E*_red_ [Ir^III*^/Ir^II^] = +0.89 V vs SCE)^31^ indicates the SET not to be feasible. We propose that the observed fluorescence quenching of **1a** is due to energy transfer from the excited photocatalyst to the alkene, an alternate pathway of quenching of excited triplet states involving no net electron transfer.^41,42^ Energy transfer has been utilized to afford *Z*-alkenes using an iridium photocatalyst.^43^ No significant fluorescence quenching was observed by the alkyl iodide **2a**.

**Scheme 4:**
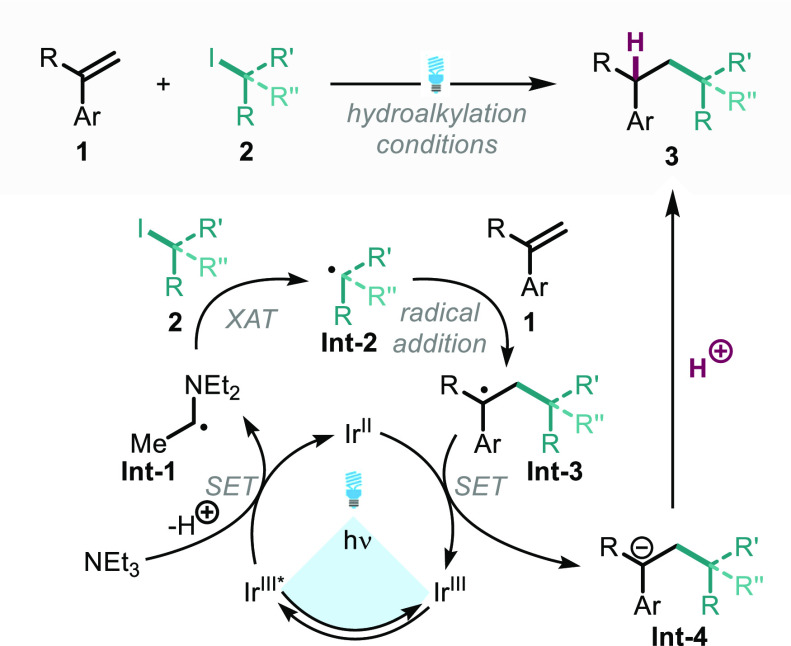
Proposed Photocatalytic Hydroalkylation Mechanism

From our study of this hydroalkylation reaction, we propose the following mechanism (Scheme 4) to furnish the desired C–C bond products **3**. Corroborating all of the experimental and literature evidence, we believe a radical-polar crossover mechanism to be operative. The Ir(III) catalyst excited by visible light undergoes SET with triethylamine (supported by fluorescence quenching studies), affording the reduced photocatalyst and oxidized amine (Et_3_N^·^^+^). The intermediate amine radical cation exhibits a significantly reduced *p*Ka for the α-C–H bond, allowing for facile deprotonation to give the α-amino radical **Int-1**.^13^ Halogen atom transfer between the α-amino radical **Int-1** and alkyl iodide **2** has been shown to be very fast (10^8^ M^–**1**^ s^–**1**^), providing key alkyl radical **Int-2**.^11,20^ The formation of this radical is further verified through the detection of its TEMPO adduct **4** (Scheme 3a). Addition of the carbon-centered radical to the aryl-alkene is well precedented, affording a highly stabilized radical **Int-3**.^26,27^ Reduction of radical **Int-3** to the anion **Int-4** by the reduced Ir(II) complex (*E*_red_ [Ir^III^/Ir^II^] = −1.37 V vs SCE)^29^ returns the ground state Ir(III) catalyst. Literature reports of single-electron reduction of substituted benzyl radicals (*E*_red_ [Bn^·^/Bn^–^] = −1.43 V vs SCE)^44^ show this potential to be beyond the reach of the photocatalyst; this result was confirmed experimentally, with no hydroalkylation of styrene observed. Furthermore, radical addition to **1a** has been demonstrated to be 30 times faster than addition to styrene.^45^ Alternatively, 4-vinyl pyridine proved to be an excellent substrate for hydroalkylation (**3r**), consistent with the “less negative” reduction potentials observed for electron poor aryl groups, which favor the radical-polar crossover process.^44^ Work by Fischer et al. has demonstrated that, while the addition of radical species to **1a** is very fast (10^7^ M^–**1**^ s^–**1**^), the radical–radical recombination of the 1,1-diphenylethyl radical with TEMPO proceeds considerably slower (10^4^ M^–**1**^ s^–**1**^).^46−48^ The observed formation of di-alkylation product **3q** indicates that, for this substrate, radical recombination between **Int-3** and **Int-2** becomes a competitive process, suggesting an accumulation of radical **Int-3**. This is further validated by the significant increase in reduction potentials for alkyl substituted benzyl radicals (*E*_red_ [3-MePhCH_2_^·^/3-MePhCH_2_^–^] = −1.50 V vs SCE),^44^ disfavoring SET to carbanion **Int-4** and leading to increased concentrations of **Int-3**. Regeneration of the Ir(III) catalyst ground state and protonation of carbanion **Int-4** close the catalytic cycle.

## Conclusions

In summary, we have presented photocatalytic hydroalkylation with aryl-alkenes, providing a net reductive C–C bond formation between aryl-alkenes and alkyl iodides. As such, we have demonstrated a substrate scope showing excellent yields and investigated the mechanism of the transformation shown. This has validated the formation of alkyl radicals *via* XAT from α-amino radicals, addition of the alkyl radicals to the aryl-alkenes, forming highly stabilized species, a radical-polar crossover pathway, and finally protonation, to furnish the desired hydroalkylation products. This is the first example of a photocatalytic hydroalkylation of aryl-alkenes using alkyl iodides.

## Experimental Section

### General Procedure for Photocatalytic Hydroalkylation

An oven-dried 8 mL screw-cap vial was charged with the photocatalyst (1 mol %), alkene (1 equiv), alkyl iodide (2 equiv), triethylamine (4 equiv), water (10 equiv), and acetonitrile (0.1 M), and the reaction was closed with a cap containing a septum. The vial content was frozen in a liquid nitrogen bath, and once frozen, a vacuum was applied. After 1 min, the reaction vial was refilled with argon (using a Schlenk line), removed from the liquid nitrogen bath, and left to thaw under argon. Once thawed, the reaction was removed from the Schlenk line and the cap was wrapped in parafilm. The reaction vial was placed in front of the light and stirred for a specified time. After this time, the reaction vial was removed from the light, concentrated *in vacuo*, and analyzed by ^1^H NMR using 1,1,2,2-tetrachloroethane (1 equiv) as an internal standard. The products were isolated from the crude material with flash chromatography using the specified conditions.

## Supporting Information Available

Additional experimental procedures, original spectral data, and materials and methods, including photographs of the experimental setup (PDF)
