# Supplementary material for: Photocatalytic Hydroalkylation of Aryl-Alkenes
Source: J Org Chem. 2022 Aug 1;87(16):11042–7. doi: 10.1021/acs.joc.2c01304 (PMC9396655; doi:10.1021/acs.joc.2c01304)
Supplement: Supplementary file 1 — jo2c01304_si_001.pdf [file jo2c01304_si_001.pdf]

Supporting Information

Photocatalytic hydroalkylation of aryl-alkenes

Cornelia S. Buettner, Michael Schnürch, Katharina Bica-Schröder*

Institute for Applied Synthetic Chemistry, TU Wien, Getreidemarkt 9/163, 1060 Vienna, Austria

*katharina.schroeder@tuwien.ac.at

General experimental S1
General procedures S3
Starting materials S4
Hydroalkylation products S8
Mechanistic studies S18
Spectra S25
References S47

General experimental

All reactions were run under an inert atmosphere (Argon) unless otherwise stated, with oven-dried glassware, using standard techniques. For photocatalytic reactions HPLC grade acetonitrile was degassed *via* freeze-pump-thaw (three cycles) before use. Petroleum ether is 40-60 bp unless stated otherwise. Reagents were used as supplied. Iridium catalysts were purchased from ABCR and used as supplied. Alkyl iodides and alkenes were synthesised using literature procedures.

Irradiation of the reaction mixture was achieved using a 40 W Kessil A160WE LED–Tuna blue aquarium light (setup: max blue, max intensity).

Analytical thin-layer chromatography (TLC) was performed on Merck Kieselgel 60 F254 0.20 mm pre-coated, aluminium backed silica gel plates. Visualisation was performed by UV absorbance (λ_{max} = 254 nm), by aq. $KMnO_4$ or by ceric ammonium nitrate (CAN) solution. Flash column chromatography was performed using silica gel (Merck Geduran Si 60, 40-63 µm) with the specified solvent system.

Nuclear magnetic resonance (NMR) spectra were recorded on a Bruker Avance 400 MHz spectrometer. Chemical shifts (δ) for ^1H NMR spectra are recorded in ppm from Me_4Si with the solvent resonance as the internal standard ($CDCl_3$ = 7.26 ppm). Data is reported as follows: chemical shift (multiplicity (s = singlet, d = doublet, t = triplet, q = quartet, p = pentet, m = multiplet, br. = broad), coupling constant, integration and molecular assignment). $^{13}C\{^1H\}$ NMR spectra are reported in ppm from Me_4Si with the solvent resonance as the internal standard ($CDCl_3$ = 77.16 ppm). ^{19}F $\{^1H\}$ NMR spectra are reported in ppm from $CFCl_3$ and are uncorrected. ^1H NMR yields were determined with 1,1,2,2-tetrachloroethane. 2D experiments (COSY, HSQC, HMBC, NOESY) were used to assign spectra but are not included. Coupling constants are quoted to the nearest 0.1 Hz.

High resolution mass spectrometry (HRMS) was carried out using an Agilent 1100/1200 HPLC with a 6230 AJS ESI-TOF MS. GCMS was carried out using a Thermo Scientific DSQ II, using a BGB5 column. Elemental analysis was conducted by the microanalytical laboratory at the University of Vienna using a Perkin Elmer 2400 CHN Elemental Analyzer. Infrared (IR) spectra were collected using a Perkin-Elmer Spectrum65 FT IS Spectrometer with absorption maxima (ν_{max}) quoted in wavenumbers (cm^{-1}). Fluorescence quenching experiments were carried out on a Perkin Elmer LS-55 fluorescence spectrometer using a quartz cuvette (d = 1 cm). Spectra were measured from 400-700 nm, using excitation wavelength 390 nm.

Photoreaction setup:

Photocatalytic reactions were irradiated by a Kessil LED A160WE Tuna Blue aquarium lamp (40 W, spectral tuning: max actinic blue, intensity: max) at a distance of approximately 3 cm. The temperature of the reactions was maintained through the use of a desk fan positioned to the side of the reaction vessel to maintain a temperature below 30 ^{o}C.

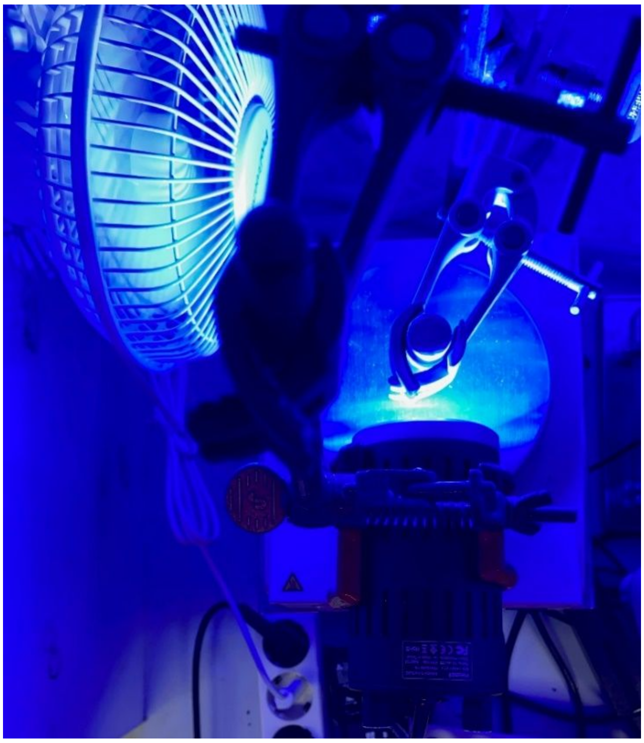

Reaction setup – top view

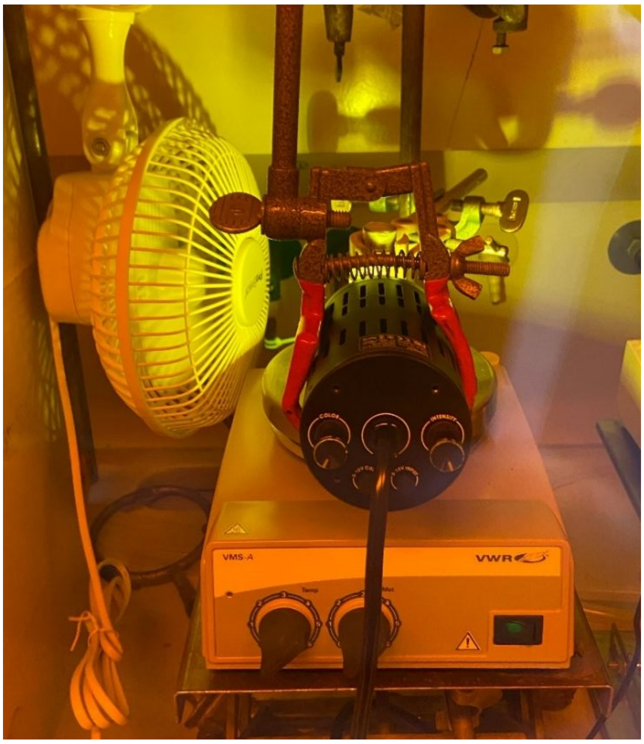

Reaction setup – front view

Although little is known about the effects of long-term exposure to high energy blue LEDs, such lamps should be used with appropriate caution. Thus, reactions were run in a designated photoreaction fumehood with a yellow screen filter.

General procedures

General Procedure A: Appel reaction

Alcohol (1 equiv), triphenylphosphine (1.2 equiv) and imidazole (1.3 equiv) was added to an oven-dried flask and dichloromethane added under argon. The reaction mixture was stirred in an ice bath and iodine (1.2 equiv) was added portionwise. The reaction was stirred, warming to room temperature. After the specified reaction time, the reaction mixture was filtered through a silica plug, washing with diethyl ether. The filtrate was concentrated *in vacuo* and purified by flash chromatography to afford the desired iodide.

General Procedure B: Wittig reaction

The phosphonium salt (1.05 equiv) was added to an oven-dried three necked flask and purged with argon. Dry THF was added to the solid and the mixture stirred while cooling to 0 ^{O}C. *n*BuLi (1.6 M in THF, 1.0 equiv) was added dropwise to the reaction. Once the addition was complete the reaction mixture was left to stir, warming to room temperature for 1 h. Subsequently, the reaction mixture was once again cooled to 0 ^{O}C and the ketone (1.0 equiv) in THF added dropwise. The reaction mixture was then stirred, warming to room temperature. The reaction was quenched by addition of a few millilitres of 1 M aq. HCl and brine. The aqueous layer was extracted by diethyl ether (2x) and the combined organic fractions dried over $MgSO_4$, filtered and concentrated *in vacuo*. The crude material was purified by flash chromatography to afford the desired olefins.

General Procedure C: Photocatalytic hydroalkylation

An oven-dried 8 mL screw-cap vial was charged with photocatalyst (1 mol%), alkene (1 equiv), alkyl iodide (2 equiv), triethylamine (4 equiv), water (10 equiv) and acetonitrile (0.1 M), and the reaction was closed with a cap containing a septum. The vial content was frozen in a liquid nitrogen bath and once frozed, a vacuum was applied. After one minute the reaction vial was refilled with argon (using a Schlenk line), removed from the liquid nitrogen bath and left to thaw under argon. Once thawed the reaction was removed from the Schlenk line and the cap wrapped in parafilm. The reaction vial was placed in front of the light and stirred for the specified time. After this time, the reaction vial was removed from the light, concentrated *in vacuo* and analysed by ^{1}H NMR using 1,1,2,2-tetrachloroethane (1 equiv) as an internal standard. The products were isolated from the crude material with flash chromatography, using the specified conditions.

Starting materials

SI-2b *iodocycloheptane*

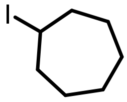

Prepared according to general procedure **A** using the corresponding alcohol and stirred for 24 h. The crude material was purified by flash chromatography (silica gel) eluting with 10% diethyl ether/petroleum ether to afford the title compound **SI-2b** isolated as a colourless oil (0.40 g, 1.8 mmol, 41%). Data consistent with literature.[1]

^{1}H NMR (400 MHz, $CDCl_3$) δ 4.48 (tt, J = 8.8, 4.3 Hz, 1H), 2.29 (dddd, J = 14.8, 7.6, 4.4, 2.9 Hz, 2H), 2.17 (dtd, J = 14.7, 9.1, 2.4 Hz, 2H), 1.66 – 1.55 (m, 6H), 1.50 – 1.39 (m, 2H). **^{13}C{^{1}H} NMR** (101 MHz, $CDCl_3$) δ 42.2, 36.5, 27.5, 27.2. **R_f** = 0.70 (petroleum ether, CAN).

SI-2c *tert-butyl 4-iodopiperidine-1-carboxylate*

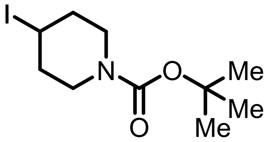

Prepared according to general procedure **A** using the corresponding alcohol and stirred for 18 h. The crude material was purified by flash chromatography (silica gel) eluting with 10-20% diethyl ether/petroleum ether to afford the title compound **SI-2b** isolated as a colourless oil (0.34 g, 1.1 mmol, 49%). Data consistent with literature.[2]

^{1}H NMR (400 MHz, $CDCl_3$) δ 4.44 (p, J = 6.0 Hz, 1H), 3.59 (dt, J = 13.3, 5.2 Hz, 2H), 3.28 (dt, J = 13.7, 5.8 Hz, 2H), 2.02 (q, J = 5.7 Hz, 4H), 1.46 (s, 9H). **^{13}C{^{1}H} NMR** (101 MHz, $CDCl_3$) δ 154.8, 79.9, 44.0, 37.5, 28.6, 28.5, 27.8. **R_f** = 0.27 (20% diethyl ether/petroleum ether, CAN).

SI-2d *tert-butyl 6-iodo-2-azaspiro[3.3]heptane-2-carboxylate*

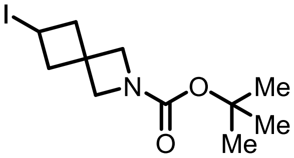

Prepared according to general procedure **A** using the corresponding alcohol and stirred for 22 h. The crude material was purified by flash chromatography (silica gel) eluting with 20% diethyl ether/petroleum ether to afford the title compound **SI-2d** as an amorphous white solid (127 mg, 0.47 mmol, 28%). Data consistent with literature.[3]

^{1}H NMR (400 MHz, $CDCl_3$) δ 4.29 (p, J = 7.8 Hz, 1H), 3.95 (s, 2H), 3.92 (s, 2H), 2.97 – 2.88 (m, 2H), 2.74 – 2.66 (m, 2H), 1.42 (s, 9H). **^{13}C{^{1}H} NMR** (101 MHz, $CDCl_3$) δ 156.2, 79.7, 47.2, 38.6, 28.5, 7.6. **R_f** = 0.09 (20% diethyl ether/petroleum ether, CAN).

SI-2e *4-iodotetrahydro-2H-pyran*

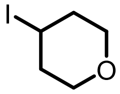

Prepared according to general procedure **A** using the corresponding alcohol and stirred for 2 d. The crude material was purified by flash chromatography (silica gel) eluting with 20% ethyl acetate/petroleum ether to afford the title compound **SI-2e** as a light yellow oil (0.57 g, 2.7 mmol, 90%). Data consistent with literature.[4]

^{1}H NMR (400 MHz, $CDCl_3$) δ 4.45 (ddd, *J* = 13.1, 7.6, 5.5 Hz, 1H), 3.82 (dt, *J* = 11.8, 4.4 Hz, 2H), 3.53 (ddd, *J* = 11.7, 7.1, 4.6 Hz, 2H), 2.23 – 2.10 (m, 4H). **^{13}C{^{1}H} NMR** (101 MHz, $CDCl_3$) δ 68.0, 38.9, 25.4. **R_f** = 0.75 (20% ethyl acetate/petroleum ether, CAN).

SI-2f *8-iodo-1,4-dioxaspiro[4.5]decane*

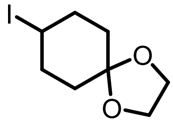

Prepared according to general procedure **A** using the corresponding alcohol and stirred for 19 h. The crude material was purified by flash chromatography (silica gel) eluting with 20-40% diethyl ether/petroleum ether to afford the title compound **SI-2f** as a colourless oil (0.96 g, 4.1 mmol, 81%). Data consistent with literature.[5]

^{1}H NMR (400 MHz, $CDCl_3$) δ 4.42 (br. s, 1H), 3.99 – 3.87 (m, 4H), 2.22 – 2.00 (m, 4H), 1.88 – 1.75 (m, 2H), 1.68 – 1.53 (m, 2H). **^{13}C{^{1}H} NMR** (101 MHz, $CDCl_3$) δ 107.6, 64.5, 64.4, 36.4, 34.9, 30.1. **R_f** = 0.24 (20% diethyl ether/petroleum ether, CAN).

SI-2g *(2-iodoethyl)benzene*

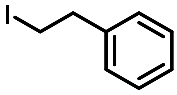

Prepared according to general procedure **A** using the corresponding alcohol and stirred for 13 h. The crude material was purified by flash chromatography (silica gel) eluting with 20% diethyl ether/hexane to afford the title compound **SI-2g** as a colourless oil (1.0 g, 4.3 mmol, 86%). Data consistent with literature.[6]

^{1}H NMR (400 MHz, $CDCl_3$) δ 7.37 – 7.27 (m, 3H), 7.23 – 7.16 (m, 2H), 3.36 (m, 2H), 3.19 (m, 2H). **^{13}C{^{1}H} NMR** (101 MHz, $CDCl_3$) δ 140.8, 128.8, 128.5, 127.0, 40.5, 5.7. **R_f** = 0.67 (hexane, UV).

SI-2h *ethyl 4-iodobutanoate*

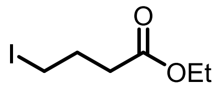

Ethyl 4-bromobutanoate (0.40 mL, 2.4 mmol), NaI (0.46 g, 3.1 mmol, 1.3 equiv) and acetone (5 mL) were added to a reaction vessel and refluxed for 24 h. After this time the reaction mixture was cooled to room temperature, the solids filtered and washed with acetone. The filtrate was concentrated *in vacuo* and the crude material was purified by flash chromatography (silica gel) eluting with 10% diethyl ether/petroleum ether to afford the title compound **SI-2h** isolated as a colourless oil (0.42 g, 1.7 mmol, 73%). Data consistent with literature.[7]

^{1}H NMR (400 MHz, $CDCl_3$) δ 4.14 (q, *J* = 7.2 Hz, 2H), 3.24 (t, *J* = 6.8 Hz, 2H), 2.44 (t, *J* = 7.2 Hz, 2H), 2.13 (p, *J* = 6.6 Hz, 2H), 1.26 (t, *J* = 7.2 Hz, 3H). **^{13}C{^{1}H} NMR** (101 MHz, $CDCl_3$) δ 172.5, 60.7, 35.0, 28.6, 14.4, 5.7. **R_f** = 0.52 (20% diethyl ether/petroleum ether, CAN).

SI-1b *4,4'-(ethene-1,1-diyl)bis((trifluoromethyl)benzene)*

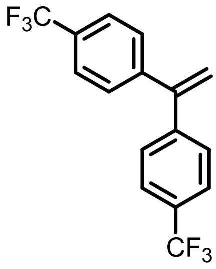

Prepared according to general procedure **B** with 4,4'-bis(trifluoromethyl)benzophenone and methyltriphenylphosphonium bromide, and stirred for 18 h. The crude material was purified by flash chromatography eluting with 5% ethyl acetate/petroleum ether to afford the title compound (0.39 g, 1.2 mmol, 42%) as a colourless oil. Data consistent with literature.[8]

^{1}H NMR (400 MHz, $CDCl_3$) δ 7.66 – 7.55 (m, 4H), 7.53 – 7.42 (m, 4H), 5.60 (s, 2H). **^{13}C{^{1}H} NMR** (101 MHz, $CDCl_3$) δ 147.9, 141.6, 131.6 (d, *J* = 1.5 Hz), 125.0 (p, *J* = 3.8 Hz), 117.26. **^{19}F{^{1}H} NMR** (376 MHz, $CDCl_3$) δ -62.67. **R_f** = 0.74 (10% ethyl acetate/petroleum ether, $KMnO_4$).

SI-1c *5-methylene-10,11-dihydro-5H-dibenzo[a,d][7]annulene*

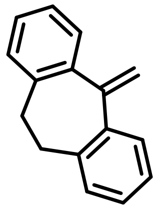

Prepared according to general procedure **B** with dibenzosuberone and methyltriphenylphosphonium bromide, and stirred for 18 h. The crude material was purified by flash chromatography eluting with 5% ethyl acetate/petroleum ether to afford the title compound (0.45 g, 2.2 mmol, 72%) as a colourless oil that solidified on standing. Data consistent with literature.[9]

^{1}H NMR (400 MHz, $CDCl_3$) δ 7.39 – 7.34 (m, 2H), 7.24 – 7.16 (m, 4H), 7.13 (dd, *J* = 7.1, 1.8 Hz, 2H), 5.43 (d, *J* = 1.6 Hz, 2H), 3.16 (s, 4H). **^{13}C{^{1}H} NMR** (101 MHz, $CDCl_3$) δ 152.0, 141.3, 138.5, 129.0, 128.3, 127.8, 126.3, 117.6, 33.4. **R_f** = 0.77 (10% ethyl acetate/petroleum ether, $KMnO_4$).

Hydroalkylation products

3a *(3-Methylbutane-1,1-diyl)dibenzene*

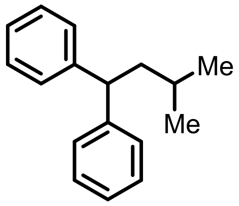

Prepared according to general procedure **C** using (Ir[dF(CF_3)ppy]$_2$(dtbpy))PF_6, 1,1-diphenylethylene, 2-iodopropane and stirred for 16 h. The reaction mixture was concentrated *in vacuo* and the crude material filtered through silica plug eluting with petroleum ether (20 mL). The title compound **3a** was isolated as a light yellow oil (19 mg, 0.085 mmol, 85%). Data consistent with literature.[10]

A large scale reaction (1.0 mmol) was run in a 25 mL RBF, according to general procedure **C** using 0.5 mol% Ir[dF(CF_3)ppy]$_2$(dtbpy))PF_6, and stirred for 16 h, affording compound **3a** as a light yellow oil (103 mg, 0.46 mmol, 46%).

^{1}H NMR (400 MHz, CDCl3) δ 7.32 – 7.23 (m, 8H), 7.21 – 7.13 (m, 2H), 4.02 (t, J = 8.0 Hz, 1H), 1.93 (dd, J = 8.0, 6.9 Hz, 2H), 1.46 (hept, J = 6.7 Hz, 1H), 0.93 (d, J = 6.6 Hz, 6H). **^{13}C{^{1}H} NMR** (101 MHz, $CDCl_3$) δ 145.4, 128.5, 128.0, 126.1, 49.0, 45.2, 25.7, 22.8. **GCMS** (EI) m/z calculated for $C_{17}H_{20}$ 224.16; found: 224.18. **IR** v_{max}/cm^{-1} (film): 2957, 2926, 2248, 1494, 1450, 1384, 906, 701. **R_f** = 0.35 (petroleum ether, UV).

3b *(2-Cyclohexylethane-1,1-diyl)dibenzene*

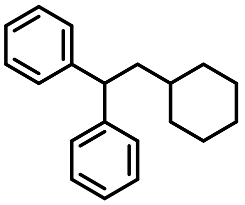

Prepared according to general procedure **C** using (Ir[dF(CF_3)ppy]$_2$(dtbpy))PF_6, 1,1-diphenylethylene, iodocyclohexane and stirred for 16 h. The reaction mixture was concentrated *in vacuo* and the crude material filtered through silica plug eluting with 0-20% diethyl ether/petroleum ether. The title compound **3b** was isolated as a light-yellow oil (21 mg, 0.080 mmol, 80%). Data consistent with literature.[10]

Analogous reaction using bromocyclohexane afforded the title product in 20% yield (determined by ^{1}H-NMR using 1,1,2,2-tetrachloroethane).

^{1}H NMR (400 MHz, $CDCl_3$) δ 7.32 – 7.23 (m, 8H), 7.20 – 7.14 (m, 2H), 4.08 (t, *J* = 8.0 Hz, 1H), 1.94 (dd, *J* = 8.0, 6.7 Hz, 2H), 1.83 – 1.73 (m, 2H), 1.65 (m, 3H), 1.24 – 1.08 (m, 4H), 1.04 – 0.90 (m, 2H). **^{13}C{^{1}H} NMR** (101 MHz, $CDCl_3$) δ 145.6, 128.5, 128.0, 126.1, 48.1, 43.8, 35.0, 33.6, 26.8, 26.3. **GCMS** (EI) m/z calculated for $C_{20}H_{24}$ 264.19; found: 264.20. **IR** v_{max}/cm^{-1} (film): 2921, 2850, 1599, 1494, 1449, 907, 732, 701. **R_f** = 0.62 (petroleum ether, UV).

3c *(2,2-diphenylethyl)cycloheptane*

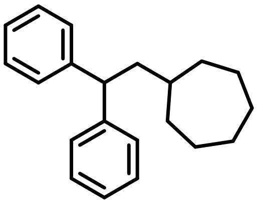

Prepared according to general procedure **C** using (Ir[dF(CF_3)ppy]$_2$(dtbpy))PF_6, 1,1-diphenylethylene, iodocycloheptane **SI-2b** and stirred for 16 h. The reaction mixture was concentrated *in vacuo* and the crude material filtered through silica plug eluting with 0-10% diethyl ether/petroleum ether. The title compound **3c** was isolated as a light yellow oil (25 mg, 0.09 mmol, 90%).

^{1}H NMR (400 MHz, $CDCl_3$) δ 7.31 – 7.21 (m, 8H), 7.20 – 7.12 (m, 2H), 4.03 (t, *J* = 7.9 Hz, 1H), 1.95 (dd, *J* = 7.9, 6.7 Hz, 2H), 1.79 – 1.70 (m, 2H), 1.66 – 1.55 (m, 2H), 1.47 (dddd, *J* = 13.2, 9.8, 6.8, 2.6 Hz, 4H), 1.41 – 1.30 (m, 3H), 1.30 – 1.20 (m, 2H). **^{13}C{^{1}H} NMR** (101 MHz, $CDCl_3$) δ 145.6, 128.5, 128.1, 126.1, 48.8, 44.1, 36.4, 34.7, 28.9, 26.2. **GCMS** (EI) m/z calculated for $C_{21}H_{26}$ 278.20; found: 278.25. **Elemental analysis** Anal. Calcd for $C_{21}H_{26}$: C, 90.59; H, 9.41; N, 0.00 Found: C, 89.84; H, 10.12; N, 0.06. **IR** ν_{max}/cm^{-1} (film): 2921, 2852, 1494, 1450, 907, 732, 702. **R$_f$** = 0.40 (petroleum ether, UV).

3d *tert-butyl 4-(2,2-diphenylethyl)piperidine-1-carboxylate*

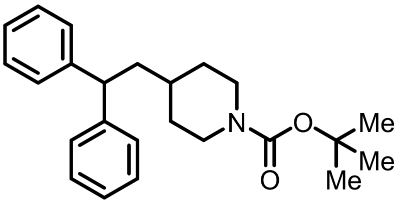

Prepared according to general procedure **C** using (Ir[dF(CF_3)ppy]$_2$(dtbpy))PF_6, 1,1-diphenylethylene, *tert*-butyl-4-iodopiperidine-1-carboxylate **SI-2c** and stirred for 16 h. The reaction mixture was concentrated *in vacuo* and the crude material filtered through silica plug eluting with 0-20% diethyl ether/petroleum ether. The title compound **3d** was isolated as a yellow oil (31 mg, 0.085 mmol, 85%). Data consistent with literature.[11]

^{1}H NMR (400 MHz, $CDCl_3$) δ 7.32 – 7.19 (m, 8H), 7.22 – 7.13 (m, 2H), 4.05 (t, *J* = 8.0 Hz, 1H), 3.36 (t, *J* = 5.1 Hz, 2H), 2.57 (t, *J* = 12.8 Hz, 2H), 1.98 (dd, *J* = 8.0, 6.7 Hz, 2H), 1.69 (d, *J* = 12.9 Hz, 2H), 1.45 (d, *J* = 6.8 Hz, 12H). **^{13}C{^{1}H} NMR** (101 MHz, $CDCl_3$) δ 155.0, 145.0, 128.7, 127.9, 126.3, 79.4, 48.0, 42.8, 33.6, 32.4, 28.6, 24.7. **GCMS** (EI) m/z calculated for $C_{24}H_{31}NO_2$ 365.24; found: 365.26 (M-Boc). **IR** ν_{max}/cm^{-1} (film): 2977, 2927, 2247, 1678 (C=O), 1426, 1165, 908, 730. **R$_f$** = 0.21 (10% diethyl ether/petroleum ether, UV).

3e *tert-butyl 3-(2,2-diphenylethyl)azetidine-1-carboxylate*

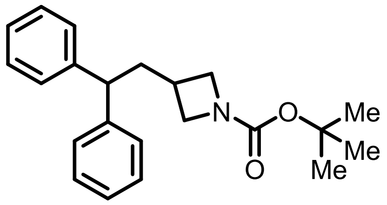

Prepared according to general procedure **C** using (Ir[dF(CF_3)ppy]$_2$(dtbpy))PF_6, 1,1-diphenylethylene, *tert*-butyl 3-iodoazetidine-1-carboxylate and stirred for 16 h. The reaction mixture was concentrated *in vacuo* and the crude material filtered through silica plug eluting with 0-20% diethyl ether/petroleum ether. The title compound **3e** was isolated as a colourless oil (21 mg, 0.062 mmol, 62%).

^{1}H NMR (400 MHz, $CDCl_3$) δ 7.32 – 7.25 (m, 4H), 7.23 – 7.15 (m, 6H), 3.89 (t, *J* = 8.2 Hz, 2H), 3.82 (t, *J* = 7.7 Hz, 1H), 3.53 (dd, *J* = 8.5, 5.5 Hz, 2H), 2.49 – 2.37 (m, 1H), 2.33 (t, *J* = 7.3 Hz, 2H), 1.42 (s, 9H). **^{13}C{^{1}H} NMR** (101 MHz, $CDCl_3$) δ 156. 5, 144.3, 128.7, 127.9, 126.6, 79.3, 49.4, 40.4, 28.6, 27.6, 15.5. **HRMS** (ESI) m/z: [M-Boc+2H]$^+$ Calcd for $C_{17}H_{20}N$ 238.1590; Found 238.1678. **GCMS** (EI) m/z calculated for $C_{22}H_{27}NO_2$ 337.20; found: 281.20 (M-*tert*-butyl). **IR** ν_{max}/cm^{-1} (film): 2970, 2878, 2247, 1689 (C=O), 1407, 1139, 908, 730, 701. **R$_f$** = 0.24 (10% diethyl ether/petroleum ether, UV).

3f *tert-butyl 6-(2,2-diphenylethyl)-2-azaspiro[3.3]heptane-2-carboxylate*

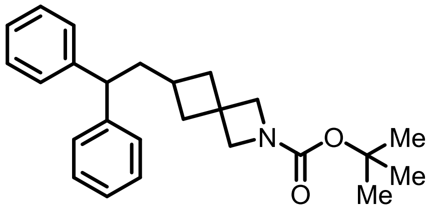

Prepared according to general procedure **C** using (Ir[dF(CF_3)ppy]$_2$(dtbpy))PF_6, 1,1-diphenylethylene, tert-butyl 6-iodo-2-azaspiro[3.3]heptane-2-carboxylate **SI-2d** and stirred for 16 h. The reaction mixture was concentrated *in vacuo* and the crude material filtered through silica plug eluting with 10% diethyl ether/petroleum ether. The title compound **3f** was isolated as a light-yellow oil (21 mg, 0.056 mmol, 56%).

^{1}H NMR (400 MHz, $CDCl_3$) δ 7.38 – 7.23 (m, 5H), 7.22 – 7.13 (m, 5H), 3.88 – 3.73 (m, 4H), 2.20 – 2.08 (m, 3H), 2.00 (h, *J* = 7.7 Hz, 1H), 1.85 – 1.71 (m, 2H), 1.42 (d, *J* = 8.5 Hz, 9H). **^{13}C{^{1}H} NMR** (101 MHz, $CDCl_3$) δ 156.4, 145.0, 128.6, 127.9, 126.3, 79.3, 49.3, 42.9, 39.2, 34.4, 33.2, 28.5, 28.2, 16.2. **HRMS** (ESI) m/z: [M-Boc+2H]$^+$ Calcd for $C_{20}H_{24}N$ 278.1903; Found 278.1909. **GCMS** (EI) m/z calculated for $C_{25}H_{31}NO_2$ 377.24; found: 277.26 (M-Boc). **IR** ν_{max}/cm^{-1} (film): 2929, 2871, 2248, 1686 (C=O), 1412, 1172, 907, 730. **R$_f$** = 0.33 (petroleum ether, UV).

3g *4-(2,2-diphenylethyl)tetrahydro-2H-pyran*

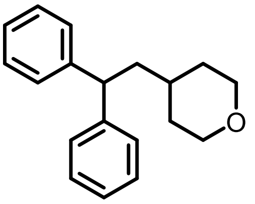

Prepared according to general procedure **C** using (Ir[dF(CF_3)ppy]$_2$(dtbpy))PF_6, 1,1-diphenylethylene, 4-iodotetrahydro-2H-pyran **SI-2e** and stirred for 16 h. The reaction mixture was concentrated *in vacuo* and the crude material filtered through silica plug eluting with 0-20% diethyl ether/petroleum ether. The title compound **3g** was isolated as a light-yellow oil (25 mg, 0.094 mmol, 94%). Data consistent with literature.[12]

^{1}H NMR (400 MHz, $CDCl_3$) δ 7.30 – 7.22 (m, 8H), 7.20 – 7.15 (m, 2H), 4.06 (t, *J* = 8.0 Hz, 1H), 3.96 – 3.83 (m, 2H), 3.26 (td, *J* = 11.0, 1.5 Hz, 2H), 2.00 (dd, *J* = 8.0, 6.3 Hz, 2H), 1.68 – 1.60 (m, 2H), 1.47 – 1.21 (m, 3H). **^{13}C{^{1}H} NMR** (101 MHz, $CDCl_3$) δ 145.0, 128.6, 127.9, 126.3, 68.0, 47.7, 43.2, 33.3, 32.6. **GCMS** (EI) m/z calculated for $C_{19}H_{22}O$ 266.17; found: 266.20. **Elemental analysis** Anal. Calcd for $C_{19}H_{22}O$: C, 85.67; H, 8.32; N, 0.00 Found: C, 83.91; H, 8.42; N, 0.08. **IR** ν_{max}/cm^{-1} (film): 2923, 2842, 2246, 1599, 1494, 1449, 907, 730. **R$_f$** = 0.58 (petroleum ether, UV).

3h *8-(2,2-diphenylethyl)-1,4-dioxaspiro[4.5]decane*

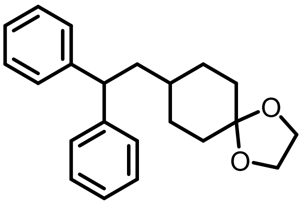

Prepared according to general procedure **C** using (Ir[dF(CF_3)ppy]$_2$(dtbpy))PF_6, 1,1-diphenylethylene, 8-iodo-1,4-dioxaspiro[4.5]decane **SI-2f** and stirred for 16 h. The reaction mixture was concentrated *in vacuo* and the crude material filtered through silica plug eluting with 0-20% diethyl ether/petroleum ether. The title compound **3h** was isolated as a colourless oil (21 mg, 0.065 mmol, 65%).

^{1}H NMR (400 MHz, $CDCl_3$) δ 7.31 – 7.21 (m, 8H), 7.20 – 7.13 (m, 2H), 4.04 (t, *J* = 8.0 Hz, 1H), 3.92 (s, 4H), 1.98 (dd, *J* = 8.0, 6.3 Hz, 2H), 1.77 (dt, *J* = 9.7, 2.6 Hz, 2H), 1.73 – 1.66 (m, 2H), 1.42 (td, *J* = 12.7, 3.7 Hz, 2H), 1.35 – 1.18 (m, 3H). **^{13}C{^{1}H} NMR** (101 MHz, $CDCl_3$) δ 145.3, 128.6, 128.0, 126.2, 109.2, 64.3, 64.3, 48.6, 42.5, 34.4, 33.8, 30.4. **HRMS** (ESI) m/z: $[M+H]^+$ Calcd for $C_{22}H_{27}O_2$ 323.2006; Found 323.2010. **IR** ν_{max}/cm^{-1} (film): 3026, 2925, 2883, 2247, 1493, 1449, 1105, 909, 731, 700. **R$_f$** = 0.25 (10% diethyl ether/petroleum ether, UV).

3i *3-(2,2-diphenylethyl)oxetane*

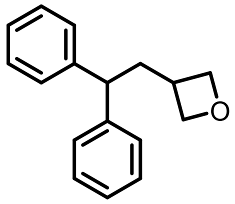

Prepared according to general procedure **C** using (Ir[dF(CF_3)ppy]$_2$(dtbpy))PF_6, 1,1-diphenylethylene, 2-iodooxetane and stirred for 16 h. The reaction mixture was concentrated *in vacuo* and the crude material filtered through silica plug eluting with 0-20% diethyl ether/petroleum ether. The title compound **3i** was isolated as a light-yellow oil (16 mg, 0.068 mmol, 68%).

^{1}H NMR (400 MHz, $CDCl_3$) δ 7.37 – 7.29 (m, 4H), 7.29 – 7.19 (m, 6H), 4.66 (dd, *J* = 7.9, 5.9 Hz, 2H), 4.36 (t, *J* = 6.3 Hz, 2H), 3.84 (t, *J* = 7.8 Hz, 1H), 3.00 (hept, *J* = 7.8 Hz, 1H), 2.49 (t, *J* = 7.8 Hz, 2H). **^{13}C{^{1}H} NMR** (101 MHz, $CDCl_3$) δ 144.3, 128.7, 127.9, 126.6, 77.6, 49.5, 39.8, 34.2. **HRMS** (ESI) m/z: $[M+H]^+$ Calcd for $C_{17}H_{19}O$ 239.1431; Found 239.1490. **GCMS** (EI) m/z calculated for $C_{17}H_{18}O$ 238.14; found: 238.22. **IR** v_{max}/cm^{-1} (film): 3027, 2962, 2870, 2247, 1600, 1494, 1450, 974, 907, 729, 701. **R$_f$** = 0.45 (petroleum ether, UV).

3j *(3,3-Dimethylbutane-1,1-diyl)dibenzene*

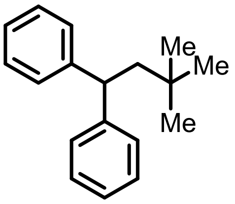

Prepared according to general procedure **C** using (Ir[dF(CF_3)ppy]$_2$(dtbpy))PF_6, 1,1-diphenylethylene, 2-iodo-2-methylpropane and stirred for 16 h. The reaction mixture was concentrated *in vacuo* and the crude material filtered through silica plug eluting with petroleum ether. The title compound **3j** was isolated as a light yellow oil (20 mg, 0.084 mmol, 84%). Data consistent with literature.[13]

^{1}H NMR (400 MHz, $CDCl_3$) δ 7.35 – 7.23 (m, 8H), 7.18 – 7.11 (m, 2H), 4.08 (t, *J* = 6.7 Hz, 1H), 2.13 (dd, *J* = 6.7, 1.9 Hz, 2H), 0.86 (d, *J* = 1.9 Hz, 9H). **^{13}C{^{1}H} NMR** (101 MHz, $CDCl_3$) δ 146.9, 128.5, 127.9, 126.0, 49.6, 48.5, 31.7, 30.4. **GCMS** (EI) m/z calculated for $C_{16}H_{18}$ 238.17; found: 238.20. **IR** v_{max}/cm^{-1} (film): 2955, 2865, 1493, 1365, 1084, 1030, 907, 731, 700. **R$_f$** = 0.67 (petroleum ether, UV).

3k *butane-1,1,4-triyltribenzene*

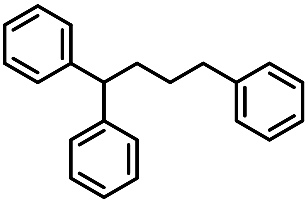

Prepared according to general procedure **C** using (Ir[dF(CF_3)ppy]$_2$(dtbpy))PF_6, 1,1-diphenylethylene, (2-iodoethyl)benzene **SI-2g** and stirred for 16 h. The reaction mixture was concentrated *in vacuo* and the crude material filtered through silica plug eluting with petroleum ether. The title compound **3k** was isolated as a colourless oil (20 mg, 95% purity, 0.066 mmol, 66%). Data consistent with literature.[14]

^{1}H NMR (400 MHz, $CDCl_3$) δ 7.29 – 7.10 (m, 15H), 3.91 (t, J = 7.8 Hz, 1H), 2.64 (t, J = 7.7 Hz, 2H), 2.16 – 1.99 (m, 2H), 1.67 – 1.55 (m, 2H). **^{13}C{^{1}H} NMR** (101 MHz, $CDCl_3$) δ 145.2, 142.5, 128.5, 128.4, 128.0, 126.2, 125.8, 51.4, 36.0, 35.4, 29.9. **GCMS** (EI) m/z calculated for $C_{22}H_{22}$ 286.17; found: 286.19. **IR** ν_{max}/cm^{-1} (film): 3026, 2935, 2859, 1495, 1452, 907, 730. **R$_f$** = 0.33 (petroleum ether, UV).

3l *(4,4,4-trifluorobutane-1,1-diyl)dibenzene*

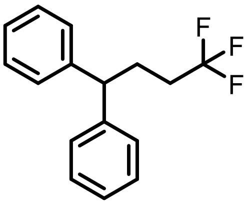

Prepared according to general procedure **C** using (Ir[dF(CF_3)ppy]$_2$(dtbpy))PF_6, 1,1-diphenylethylene, 1,1,1-trifluoro-2-iodoethane and stirred for 16 h. The reaction mixture was concentrated *in vacuo* and the crude material filtered through silica plug eluting with 0-20% diethyl ether/petroleum ether. The title compound **3l** was isolated as a light-yellow oil (21 mg, 0.079 mmol, 79%). Data consistent with literature.[15]

^{1}H NMR (400 MHz, $CDCl_3$) δ 7.39 – 7.16 (m, 10H), 3.92 (t, J = 8.0 Hz, 1H), 2.36 – 2.26 (m, 2H), 2.13 – 1.92 (m, 2H). **^{13}C{^{1}H} NMR** (101 MHz, $CDCl_3$) δ 143.7, 128.9, 127.8, 126.8, 50.4, 32.6 (q, $^1J_{C\text{-}F}$ = 28.6 Hz), 27.9, 27.9. **^{19}F{^{1}H} NMR** (376 MHz, $CDCl_3$) δ -66.18. **GCMS** (EI) m/z calculated for $C_{16}H_{15}F_3$ 264.11; found: 264.11. **IR** ν_{max}/cm^{-1} (film): 3029, 1495, 1451, 1258, 1138, 908, 732, 702. **R$_f$** = 0.29 (petroleum ether, UV).

3m *ethyl 6,6-diphenylhexanoate*

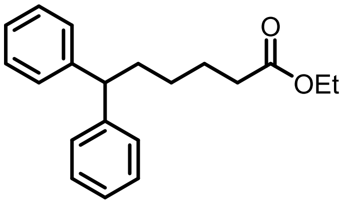

Prepared according to general procedure **C** using (Ir[dF(CF_3)ppy]$_2$(dtbpy))PF_6, 1,1-diphenylethylene, ethyl 4-iodobutanoate **SI-2h** and stirred for 16 h. The reaction mixture was concentrated *in vacuo* and the crude material filtered through silica plug eluting with 0-20% diethyl ether/petroleum ether. The title compound **3m** was isolated as a light-yellow oil (21 mg, 0.079 mmol, 79%).

^{1}H NMR (400 MHz, $CDCl_3$) δ 7.30 – 7.21 (m, 8H), 7.19 – 7.14 (m, 2H), 4.09 (q, *J* = 7.1 Hz, 2H), 3.89 (t, *J* = 7.8 Hz, 1H), 2.25 (t, *J* = 7.6 Hz, 2H), 2.11 – 1.99 (m, 2H), 1.66 (p, *J* = 7.6 Hz, 2H), 1.34 – 1.25 (m, 2H), 1.22 (t, *J* = 7.1 Hz, 3H). **^{13}C{^{1}H} NMR** (101 MHz, $CDCl_3$) δ 173.8, 145.2, 128.6, 128.0, 126.2, 60.3, 51.3, 35.5, 34.4, 27.7, 25.1, 14.4. **HRMS** (ESI) m/z: $[M+H]^+$ Calcd for $C_{20}H_{25}O_2$ 297.1849; Found 297.1852. **IR** ν_{max}/cm^{-1} (film): 3062, 2936, 1726 (C=O), 1494, 1451, 1095, 907, 729. **R$_f$** = 0.44 (10% diethyl ether/petroleum ether, UV).

3n *butane-1,1-diyldibenzene*

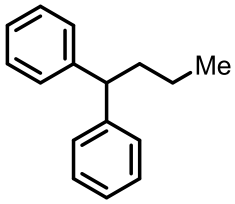

Prepared according to general procedure **C** using (Ir[dF(CF_3)ppy]$_2$(dtbpy))PF_6, 1,1-diphenylethylene, iodoethane and stirred for 16 h. The reaction mixture was concentrated *in vacuo* and the crude material filtered through silica plug eluting with 0-20% diethyl ether/petroleum ether. The title compound **3n** was isolated as a light yellow oil (16 mg, 0.076 mmol, 76%). Data consistent with literature.[16]

^{1}H NMR (400 MHz, $CDCl_3$) δ 7.32 – 7.20 (m, 8H), 7.16 (ddt, *J* = 7.4, 6.0, 2.2 Hz, 2H), 3.91 (t, *J* = 7.8 Hz, 1H), 2.08 – 1.96 (m, 2H), 1.36 – 1.21 (m, 2H), 0.92 (t, *J* = 7.4 Hz, 3H). **^{13}C{^{1}H} NMR** (101 MHz, $CDCl_3$) δ 145.5, 128.5, 128.0, 126.1, 51.2, 38.1, 21.3, 14.2. **GCMS** (EI) calculated for $C_{16}H_{18}$ 210.14; m/z: 210.16. **IR** ν_{max}/cm^{-1} (film): 3026, 2958, 2931, 2871, 1600, 1493, 1450, 1031, 907, 730. **R$_f$** = 0.31 (petroleum ether, UV).

3o *hex-5-ene-1,1-diyldibenzene*

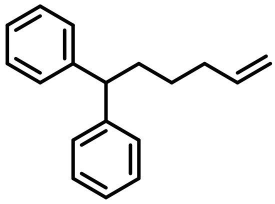

Prepared according to general procedure **C** using (Ir[dF(CF_3)ppy]$_2$(dtbpy))PF_6, 1,1-diphenylethylene, (iodomethyl)cyclopropane and stirred for 16 h. The reaction mixture was concentrated *in vacuo* and the crude material filtered through silica plug eluting with petroleum ether. The title compound **3o** was isolated as a light-yellow oil (38 mg, 0.16 mmol, 80%). Data consistent with literature.[17]

^{1}H NMR (400 MHz, $CDCl_3$) δ 7.29 – 7.20 (m, 8H), 7.18 – 7.12 (m, 2H), 5.75 (ddt, *J* = 17.0, 10.2, 6.7 Hz, 1H), 4.97 (dq, *J* = 17.2, 1.7 Hz, 1H), 4.92 (ddt, *J* = 10.1, 2.3, 1.2 Hz, 1H), 3.88 (t, *J* = 7.8 Hz, 1H), 2.06 (dtd, *J* = 10.7, 7.5, 5.5 Hz, 4H), 1.36 (tt, *J* = 10.7, 6.3 Hz, 2H). **^{13}C{^{1}H} NMR** (101 MHz, $CDCl_3$) δ 145.3, 138.8, 128.5, 128.0, 126.2, 114.7, 51.4, 35.3, 33.97, 27.5. **GCMS** (EI) m/z calculated for $C_{18}H_{20}$ 236.16; found: 236.18. **IR** ν_{max}/cm^{-1} (film): 3061, 3026, 2930, 2860, 1494, 1450, 913, 700. **R$_f$** = 0.37 (petroleum ether, $KMnO_4$).

3p *4,4'-(3-methylbutane-1,1-diyl)bis((trifluoromethyl)benzene)*

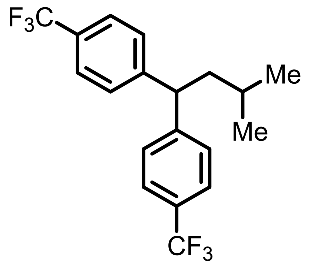

Prepared according to general procedure **C** using (Ir[dF(CF_3)ppy]$_2$(dtbpy))PF_6, 4,4'-(ethene-1,1-diyl)bis((trifluoromethyl)benzene) **SI-1b**, 2-iodopropane and stirred for 16 h. The reaction mixture was concentrated *in vacuo* and the crude material filtered through silica plug eluting with petroleum ether. The title compound **3p** was isolated as a light-yellow oil (58 mg, 0.16 mmol, 80%).

^{1}H NMR (400 MHz, $CDCl_3$) δ 7.58 – 7.35 (m, 8H), 4.14 (t, *J* = 8.0 Hz, 1H), 1.99 – 1.89 (m, 2H), 1.41 (dp, *J* = 13.3, 6.7 Hz, 1H), 0.94 (d, *J* = 6.6 Hz, 6H). **^{13}C{^{1}H} NMR** (101 MHz, $CDCl_3$) δ 145.4, 131.36, 131.35, 129.25, 124.61 (q, *J* = 3.8 Hz), 123.54 (q, *J* = 3.8 Hz), 48.7, 44.8, 25.6, 22. 7. **^{19}F{^{1}H} NMR** (376 MHz, $CDCl_3$) δ -62.53. **GCMS** (EI) m/z calculated for $C_{19}H_{18}F_6$ 360.13; found: 360.20. **Elemental analysis** Anal. Calcd for $C_{19}H_{18}F_6$: C, 63.33; H, 5.04; N, 0.00 Found: C, 59.79; H, 4.85; N, 0.73. **IR** ν_{max}/cm^{-1} (film): 2959, 2930, 2872, 1448, 1326, 1163, 1122, 707. **R$_f$** = 0.56 (petroleum ether, UV).

3q *5-isobutyl-10,11-dihydro-5H-dibenzo[a,d][7]annulene*

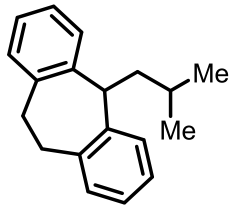

Prepared according to general procedure **C** using (Ir[dF(CF_3)ppy]$_2$(dtbpy))PF_6, 5-methylene-10,11-dihydro-5H-dibenzo[a,d][7]annulene **SI-1c**, 2-iodopropane and stirred for 16 h. The reaction mixture was concentrated *in vacuo* and the crude material flushed through a silica plug eluting with petroleum ether and then purified by flash chromatography eluting with petroleum ether. The title compound **3q** was isolated as a colourless oil (16 mg, 0.03 mmol, 32%).

^{1}H NMR (400 MHz, $CDCl_3$) δ 7.18 (ddt, *J* = 7.2, 4.4, 2.5 Hz, 2H), 7.15 – 7.07 (m, 6H), 4.12 (br. s, 1H), 3.36 (br. s, 2H), 3.05 (br. s, 2H), 2.03 – 1.90 (m, 2H), 1.51 – 1.39 (m, 1H), 0.94 (d, *J* = 6.6 Hz, 6H). **^{13}C{^{1}H} NMR** (101 MHz, $CDCl_3$) δ 142.4, 139.5, 130.4, 129.0, 128.3, 127.8, 126.4, 126.1, 33.3, 26.0, 22.9. **GCMS** (EI) m/z calculated for $C_{19}H_{22}$ 250.17; found: 250.19. **Elemental analysis** Anal. Calcd for $C_{19}H_{22}$: C, 91.14; H, 8.86; N, 0.00 Found: C, 87.68; H, 9.74; N, 0.16. **IR** ν_{max}/cm^{-1} (film): 2953, 2927, 2867, 1493, 1466, 1383, 1365, 908, 734. **R$_f$** = 0.20 (petroleum ether, CAN).

3q-b *5-isobutyl-5-isopropyl-10,11-dihydro-5H-dibenzo[a,d][7]annulene*

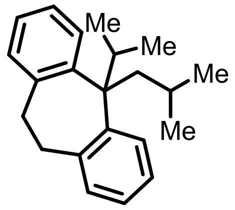

3q-b isolated as a side product from the reaction of 5-methylene-10,11-dihydro-5H-dibenzo[a,d][7]annulene **SI-1c**, purified by flash chromatography eluting with petroleum ether. The title compound **3q-b** was isolated as a colourless oil (6 mg, 0.02 mmol, 10%).

^{1}H NMR (400 MHz, $CDCl_3$) δ 7.57 (dd, *J* = 8.3, 1.3 Hz, 2H), 7.18 (ddd, *J* = 8.3, 7.0, 1.9 Hz, 2H), 7.09 (td, *J* = 7.3, 1.3 Hz, 2H), 7.04 (dd, *J* = 7.5, 1.9 Hz, 2H), 3.08 (ddd, *J* = 15.0, 9.5, 2.8 Hz, 2H), 2.93 (ddd, *J* = 14.1, 9.3, 2.5 Hz, 2H), 2.56 (dq, *J* = 13.1, 6.6 Hz, 1H), 2.26 (d, *J* = 5.6 Hz, 2H), 1.24 – 1.17 (m, 1H), 0.63 (d, *J* = 6.8 Hz, 6H), 0.48 (d, *J* = 6.7 Hz, 6H). **^{13}C{^{1}H} NMR** (101 MHz, $CDCl_3$) δ 145.3, 131.5, 129.8, 125.6, 125.1, 59.5, 51.4, 42.7, 38.9, 26.1, 24.4, 19.3. **GCMS** (EI) m/z calculated for $C_{22}H_{28}$ 292.22; found: 292.22. **IR** ν_{max}/cm^{-1} (film): 2955, 2868, 1490, 1466, 1446, 1384, 1365, 908, 732. **R$_f$** = 0.40 (petroleum ether, CAN).

3r *4-isopentylpyridine*

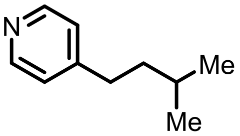

Prepared according to general procedure **C** using (Ir[dF(CF_3)ppy]$_2$(dtbpy))PF_6, 4-vinylpyridine, 2-iodopropane and stirred for 16 h. The reaction mixture was concentrated *in vacuo* and the crude material filtered through silica plug eluting with 5% methanol/ethyl acetate to afford the title compound **3r** as a yellow oil (23 mg, 0.15 mmol, 77%). Data consistent with literature.[18]

^{1}H NMR (400 MHz, $CDCl_3$) δ 8.47 (dt, *J* = 4.5, 1.3 Hz, 2H), 7.12 (ddd, *J* = 4.5, 1.6, 0.8 Hz, 2H), 2.74 – 2.46 (m, 2H), 1.58 (dq, *J* = 11.7, 5.9 Hz, 1H), 1.56 – 1.45 (m, 2H), 0.94 (dd, *J* = 6.4, 0.9 Hz, 6H). **^{13}C{^{1}H} NMR** (101 MHz, $CDCl_3$) δ 152.5, 149.5, 124.1, 39.7, 33.3, 27.8, 22.6. **HRMS** (ESI) m/z: [M+H]$^+$ Calcd for $C_{10}H_{16}N$ 150.1277; Found 150.1280. **IR** v_{max}/cm^{-1} (film): 2956, 2928, 2870, 1602. 1466, 1416, 1367, 1218, 918, 847, 731. **R$_f$** = 0.43 (ethyl acetate, $KMnO_4$).

3s *4-(3-methyl-1-(3,5,5,8,8-pentamethyl-5,6,7,8-tetrahydronaphthalen-2-yl)butyl)benzoic acid*

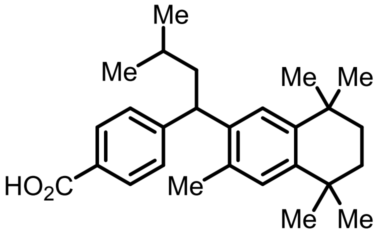

Prepared according to general procedure **C** (with 5 equiv. of triethylamine) using (Ir[dF(CF_3)ppy]$_2$(dtbpy))PF_6, bexarotene, 2-iodopropane and stirred for 16 h. The reaction mixture was concentrated *in vacuo*, HCl (5 equiv. in dioxane) added and the crude material filtered through silica plug eluting with 0-20% diethyl ether/petroleum ether. The title compound **3s** was isolated as a light-yellow oil (23 mg, 0.059 mmol, 59%).

^{1}H NMR (400 MHz, $CDCl_3$) δ 8.05 – 7.96 (m, 2H), 7.31 – 7.27 (m, 2H), 7.24 (s, 1H), 7.00 (s, 1H), 4.20 (t, *J* = 7.8 Hz, 1H), 2.19 (s, 3H), 1.94 – 1.83 (m, 2H), 1.72 – 1.60 (m, 4H), 1.50 (hept, *J* = 6.5 Hz, 1H), 1.30 (s, 3H), 1.26 (s, 6H), 1.23 (s, 3H), 0.96 (d, *J* = 6.6 Hz, 3H), 0.90 (d, *J* = 6.6 Hz, 3H). **^{13}C{^{1}H} NMR** (101 MHz, $CDCl_3$) δ 172.0, 152.0, 142.7, 142.4, 138.7, 133.2, 130.4, 128.6, 128.5, 126.9, 124.8, 45.7, 44.9, 35.4, 35.3, 34.2, 33.9, 32.2, 32.1, 32.0, 31.9, 25.7, 23.2, 22.6, 19.6. **HRMS** (ESI) m/z: [M-H]$^-$ Calcd for $C_{27}H_{35}O_2$ 391.2642; Found 391.2643. **IR** v_{max}/cm^{-1} (film): 2957, 2926, 1688 (C=O), 1422, 1285, 908, 733. **R$_f$** = 0.18 (20% diethyl ether/petroleum ether, $KMnO_4$).

Mechanistic studies

a. Deuteration studies

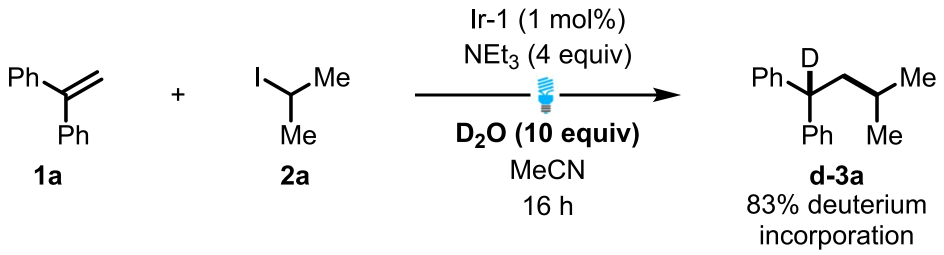

Prepared according to general procedure **C** using D_2O, $(Ir[dF(CF_3)ppy]_2(dtbpy))PF_6$, 1,1-diphenylethylene, 2-iodopropane and stirred for 16 h. The reaction mixture was concentrated *in vacuo* and filtered through silica with petroleum ether to remove the triethylamine, and the product analysed by ^{1}H-NMR demonstrating deuteration of 83:17 D:H (67%).

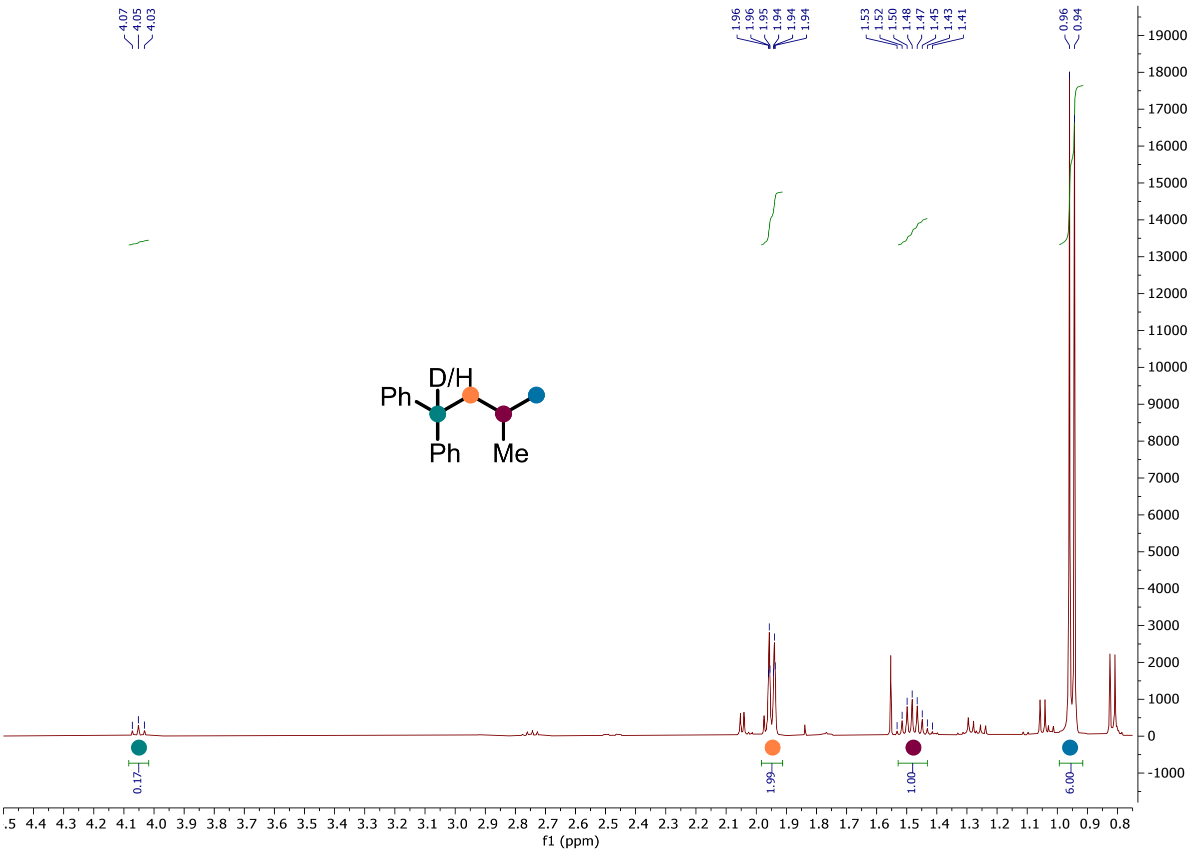

83% deuterated product

17% protonated product

b. NMR reaction

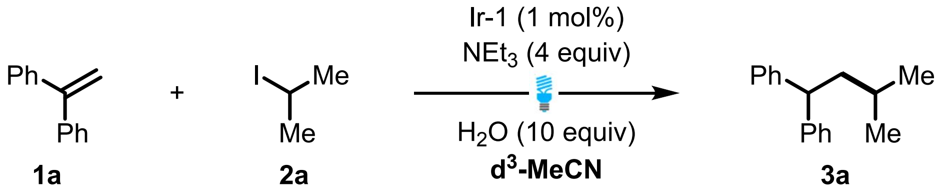

Prepared according to general procedure **C** using (Ir[dF(CF$_3$)ppy]$_2$(dtbpy))PF$_6$, deuterated acetonitrile (degassed by bubbling through argon for 0.5 h) and model substrates: 1,1-diphenylethylene and 2-iodopropane. 0.5 mL of the reaction mixture was transferred to an NMR tube, briefly flushed with argon, capped and the top wrapped in parafilm. The reaction was then analysed by 1H-NMR and subsequently at set intervals after designated periods of irradiation by blue light.

Stacked spectra:

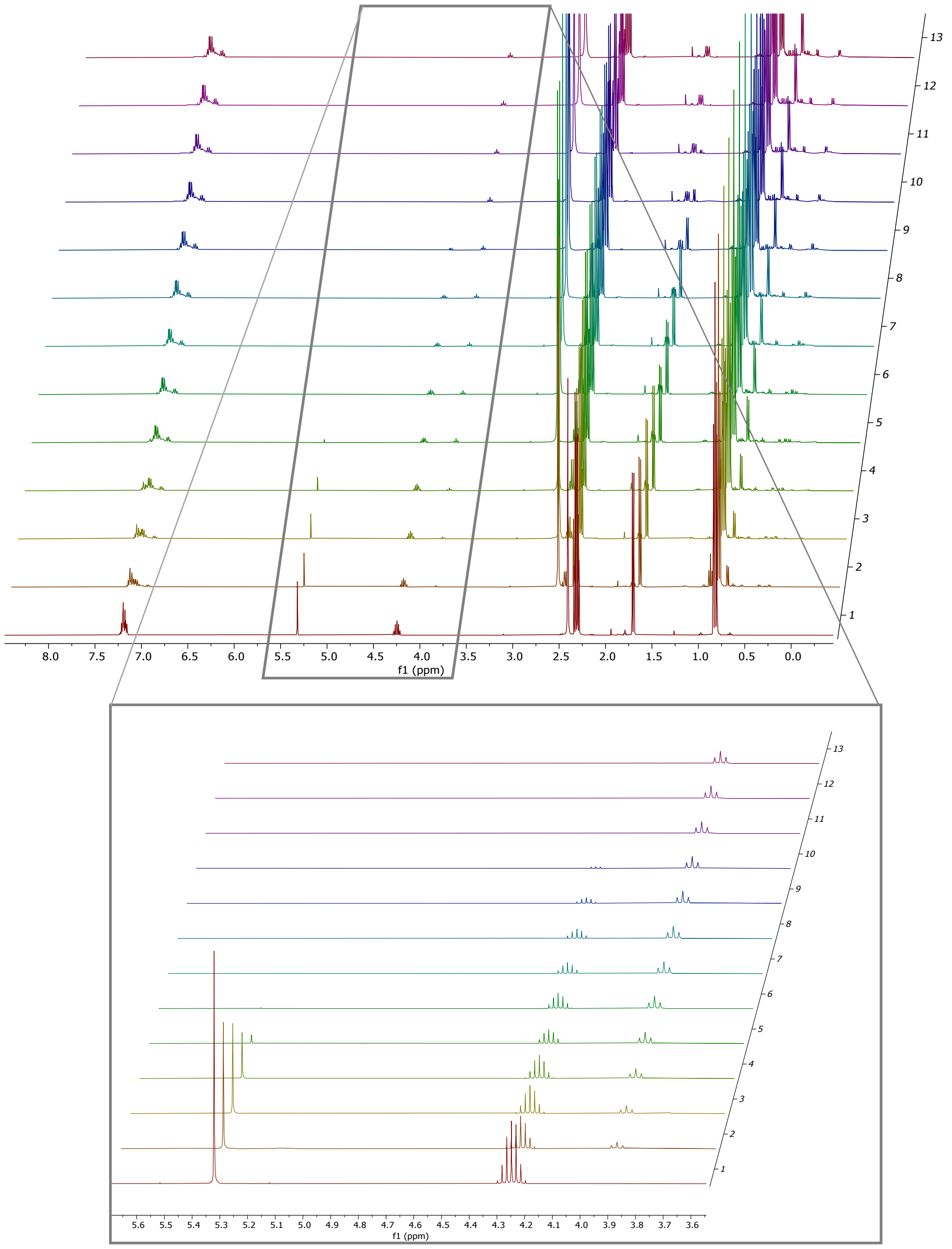

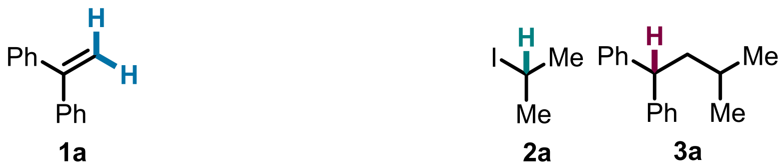

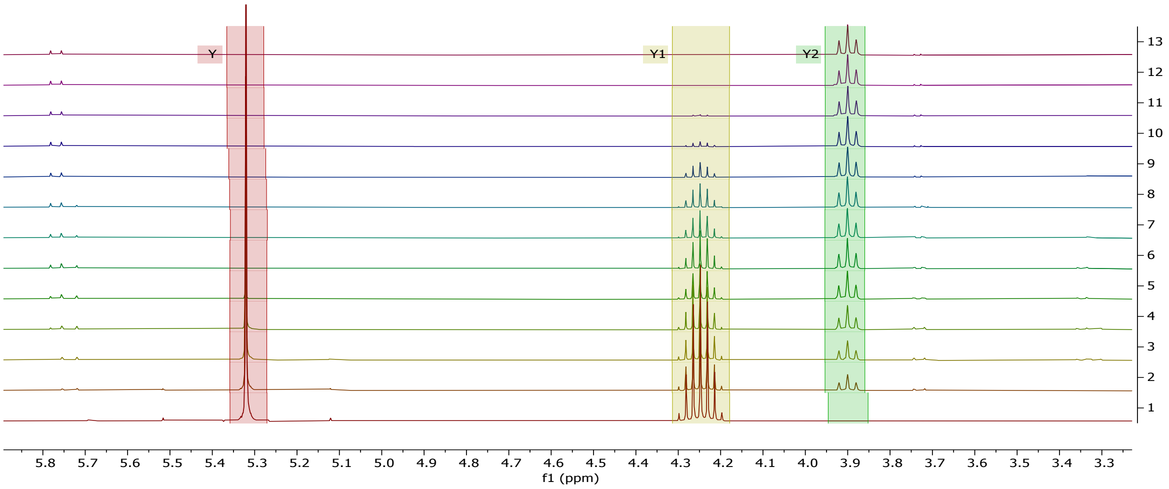

Integration values for compounds **1a**, **2a** and **3a**:

Spectrum #	Cumulative irradiation time (h)	1a / AU	2a / AU	3a / AU
1	0	63371.1	51150.8	0
2	0.25	33869.5	24311.6	5403.8
3	0.5	22960.2	20352.4	6458.22
4	1.0	12471.8	15816.8	8698.13
5	1.5	2851.33	14693.9	10716.9
6	2.0	253.95	11015.0	10647.9
7	2.5	0	8798.56	11735.3
8	3.0	0	7049.50	11617.0
9	4.0	0	4695.42	10630.5
10	5.0	0	1628.44	11006.8
11	6.0	0	537.49	11929.4
12	7.0	0	0	12210.9
13	8.0	0	0	11183.3

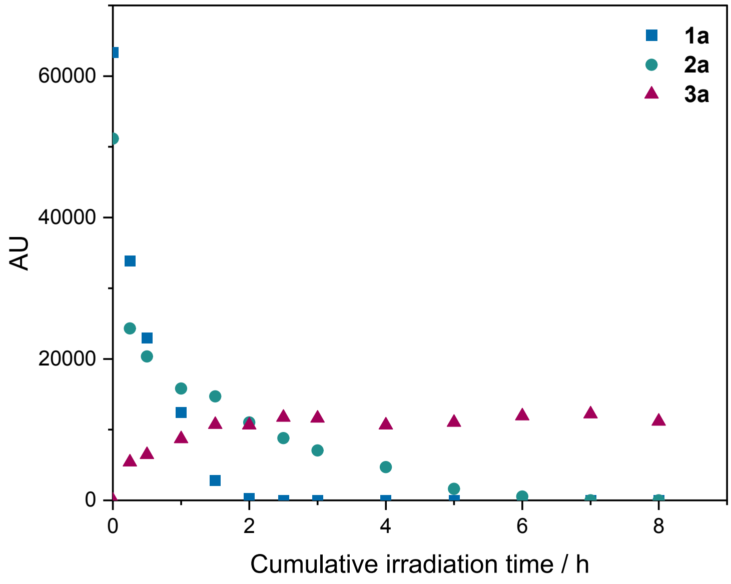

c. TEMPO reaction

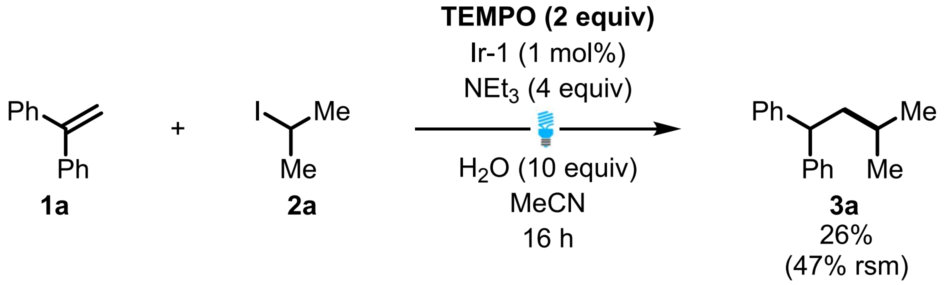

Prepared according to general procedure **C** with the inclusion of TEMPO (2 equiv) using $(Ir[dF(CF_3)ppy]_2(dtbpy))PF_6$, 1,1-diphenylethylene, 2-iodopropane and stirred for 16 h. The reaction mixture was analysed by GCMS.

GCMS analysis of *<u>TEMPO reaction</u>*

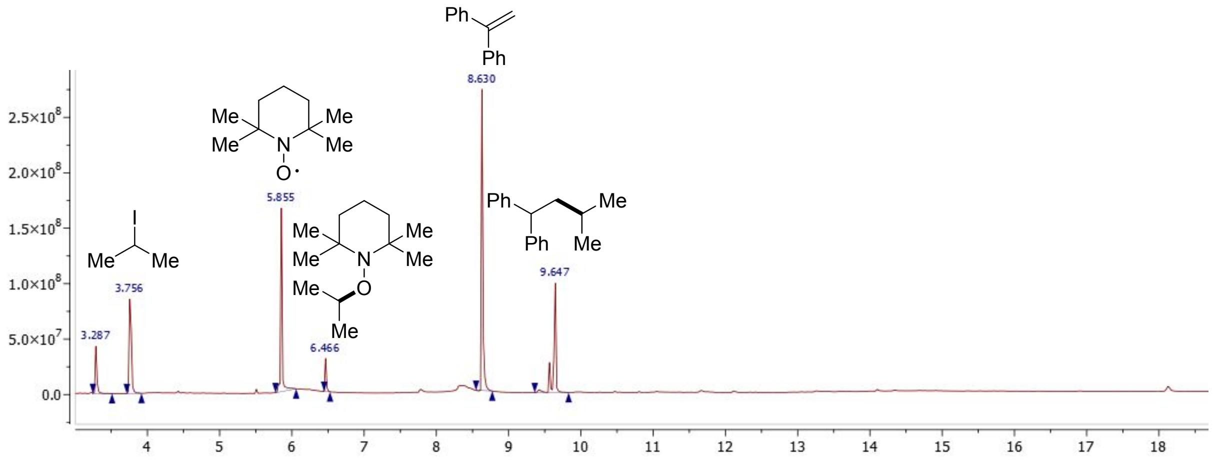

GCMS analysis of *<u>standard reaction</u>*

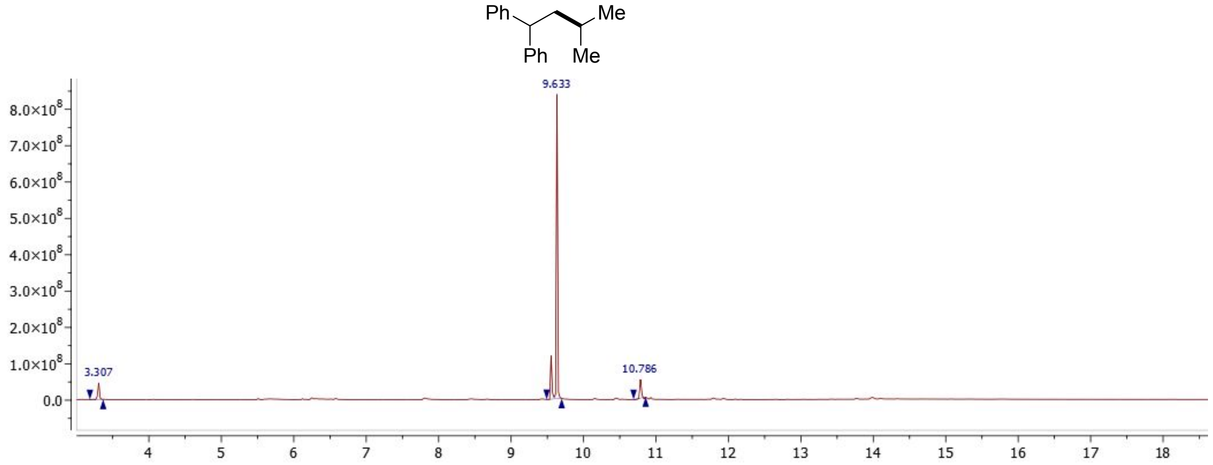

[1]*H-NMR analysis* showed mostly remaining starting material, with some conversion to the hydroalkylation product. 1,1,2,2-tetrachloroethane used as internal standard, showing 26% product and 47% starting material remaining.

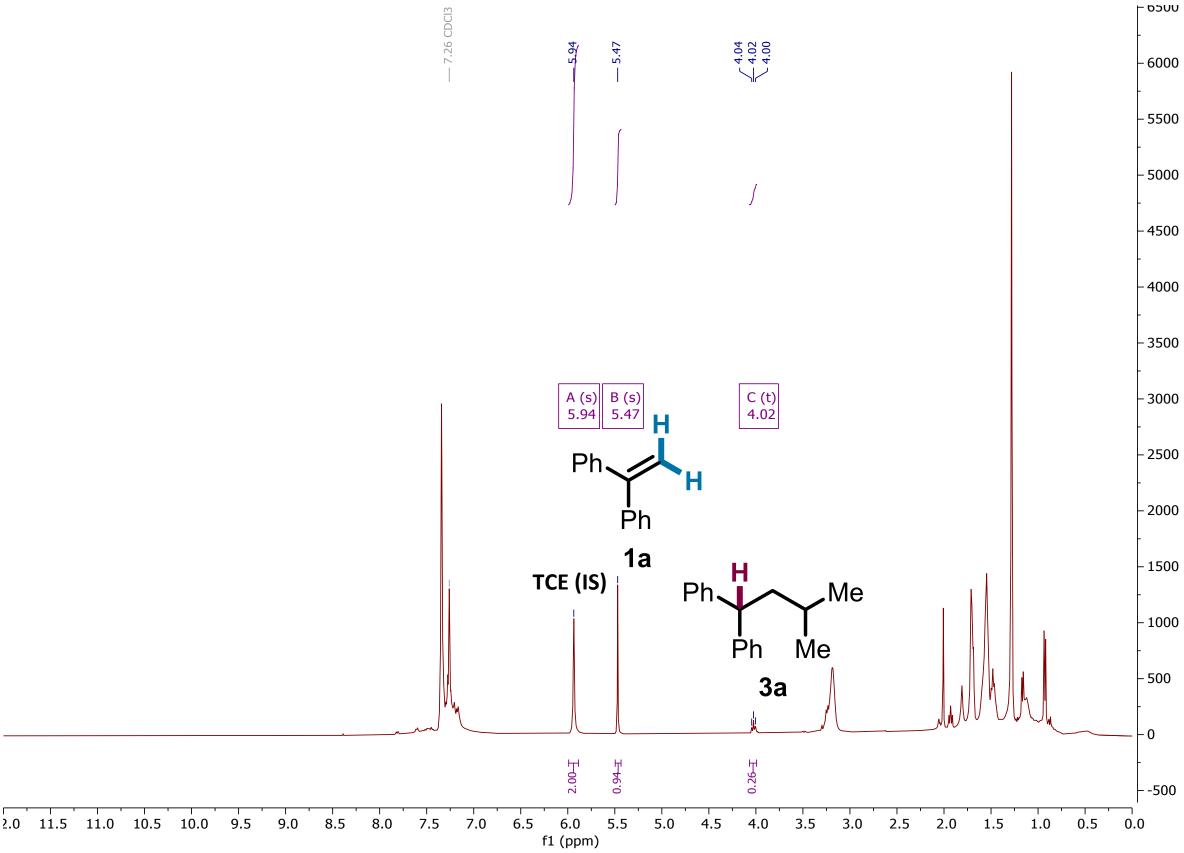

26% product : 47% starting material

d. Fluorescence quenching experiments

Stern Volmer quenching studies were done with the key components of the hydroalkylation reaction: amine, alkene **1a** and alkyl iodide **2a**. The iridium photocatalyst [Ir[(dF(CF_3)ppy)]$_2$(dtbbpy)]PF_6 ($3x10^{-5}$ M in degassed acetonitrile) was excited at 390 nm and the emission spectrum recorded at λ_{max} = 485 nm. 0.2 M Stock solutions of triethylamine, 1,1-diphenylethylene and 2-iodopropane were prepared and 12 µL added to the photocatalyst (cuvette V = 3 mL) sequentially. Quenching of the photocatalyst was observed with both the amine and the alkene, no such effect was seen for the alkyl iodide indicating no interaction between it and the catalyst excited state.

Stern-Volmer plot:

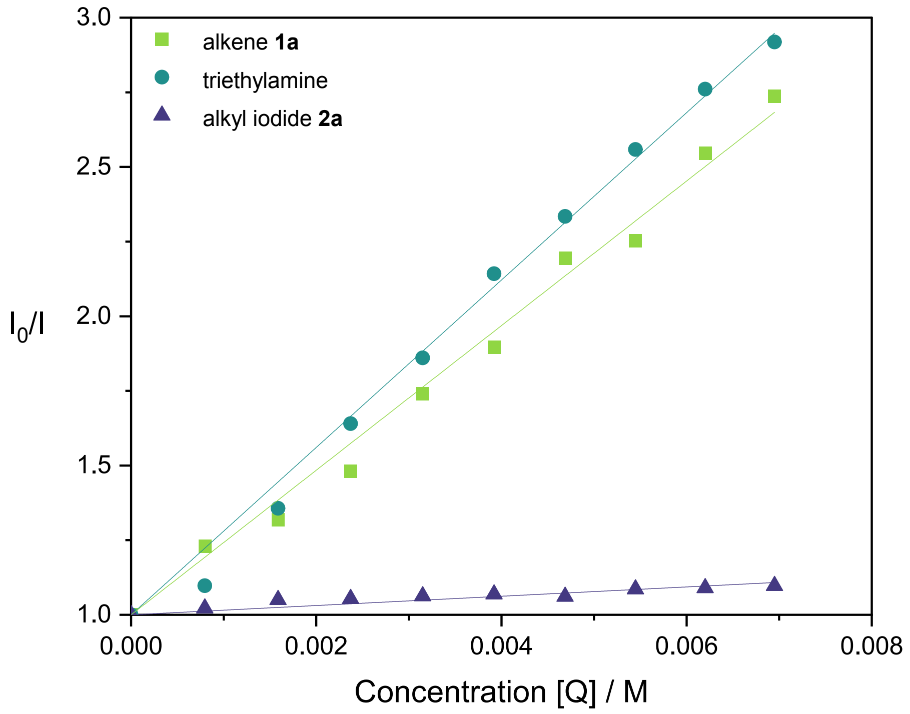

Using the Stern-Volmer relationship: $\frac{I_o}{I} = \mathbf{1} + \boldsymbol{K_q \tau_0 [Q]}$ the Stern-Volmer quenching constant (K_{SV}) can be determined from the slope of the graph above. Using the relationship $\boldsymbol{K_{SV} = K_q \tau_0}$, the quenching constant (**k_q**) for each quencher can be calculated.

Lifetime of photocatalyst [Ir[(dF(CF_3)ppy)]$_2$(dtbbpy)]PF_6 = 2.3 µs[19]

Component	Stern-Volmer constant K_{SV} / M^{-1}	Trendline R^2	K_q / M^{-1} s^{-1}
1,1-diphenylethylene **1a**	242	0.99	$1.1x10^8$
triethylamine	280	0.99	$1.2x10^8$
2-iodopropane **2a**	16	0.99	$7.0x10^6$

Fluorescence quenching spectra:

Amine

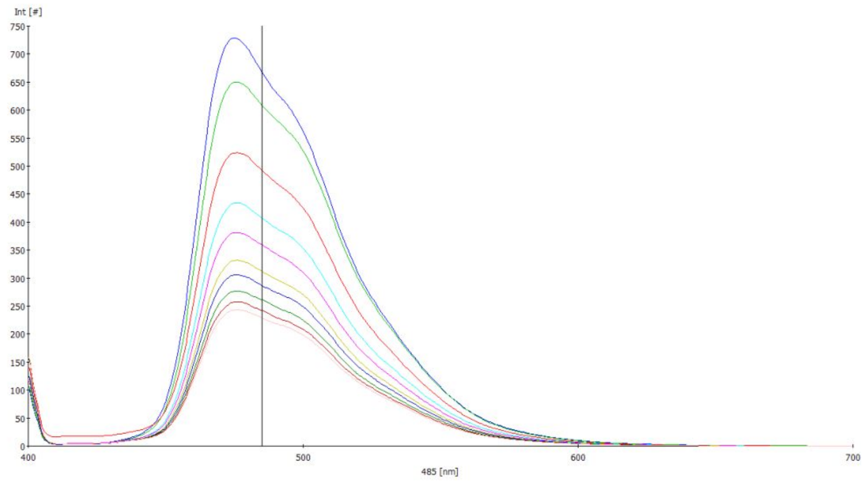

Alkene

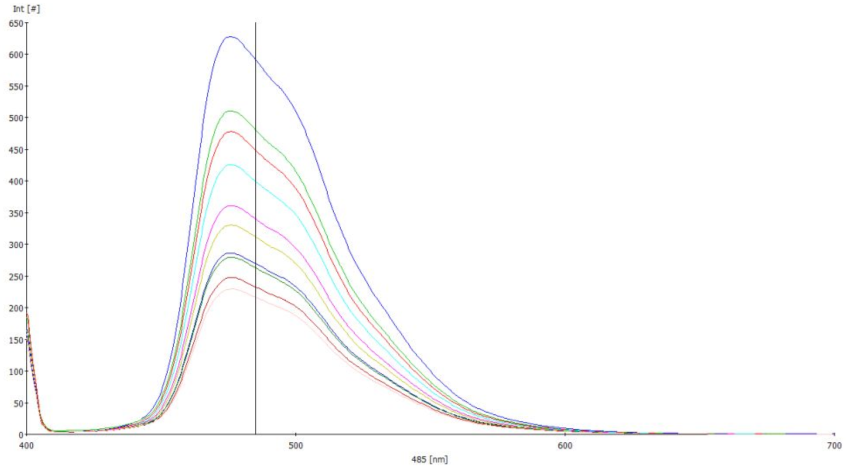

Alkyl iodide

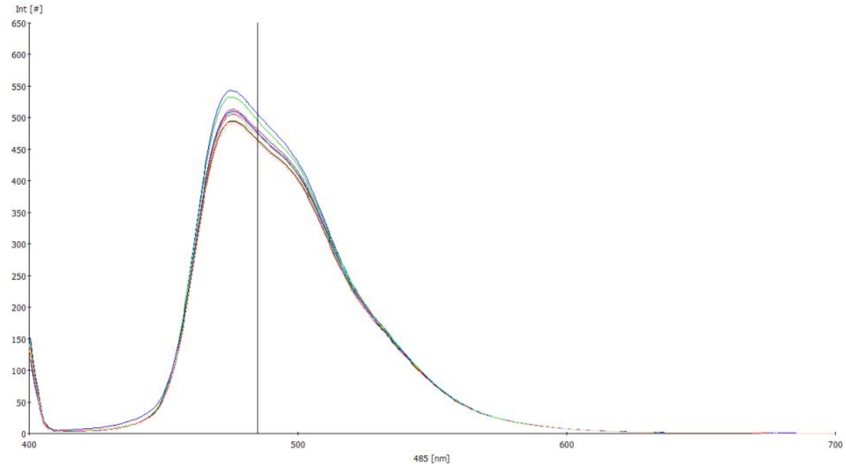

Spectra

^{1}H NMR (400 MHz, $CDCl_3$) spectrum of **3a**

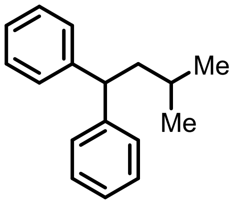

3a

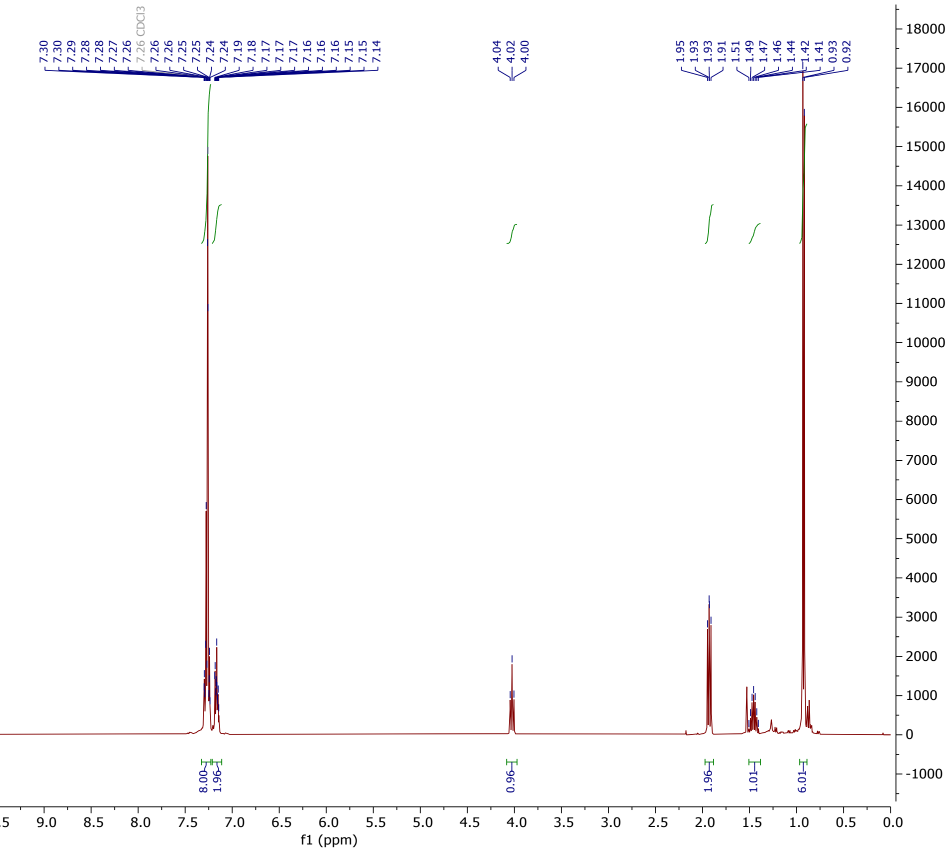

$^{13}C\{^{1}H\}$ NMR (101 MHz, $CDCl_3$) spectrum of **3a**

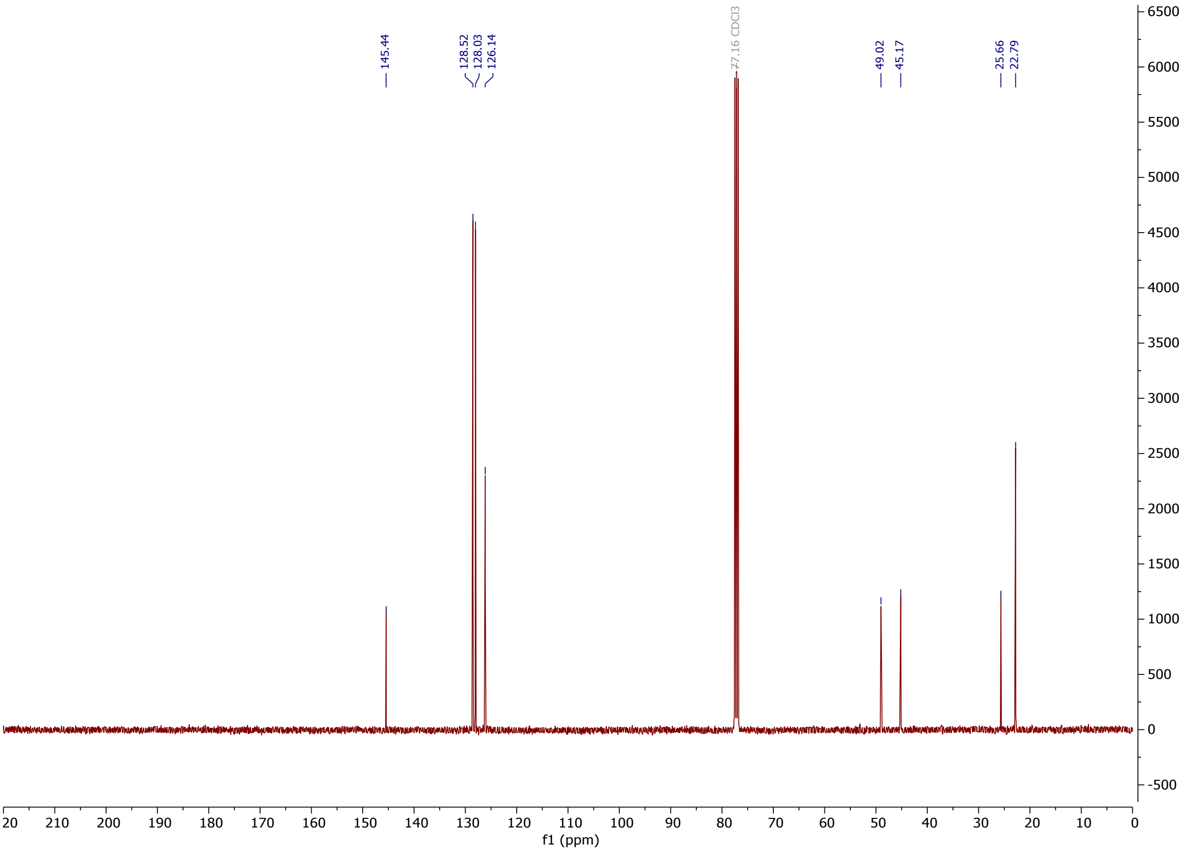

^{1}H NMR (400 MHz, $CDCl_3$) spectrum of **3b**

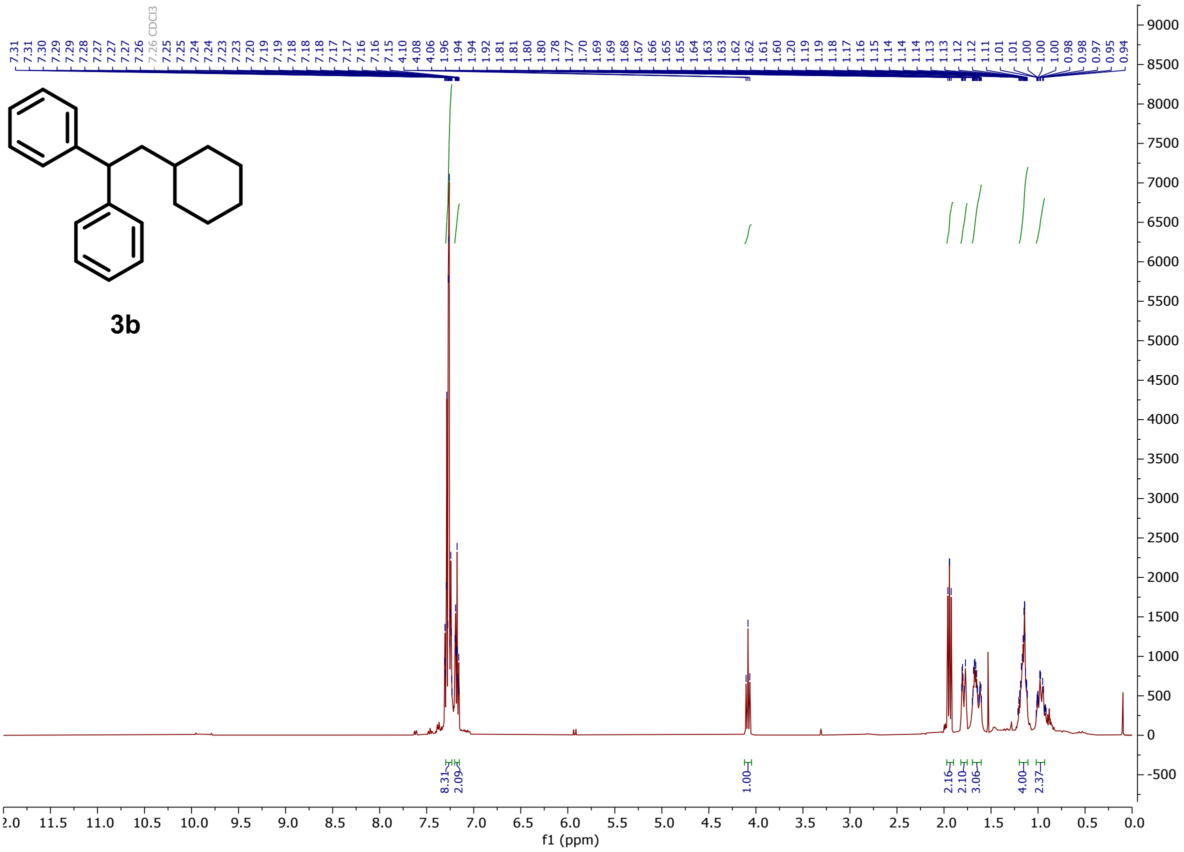

$^{13}C\{^{1}H\}$ NMR (101 MHz, $CDCl_3$) spectrum of **3b**

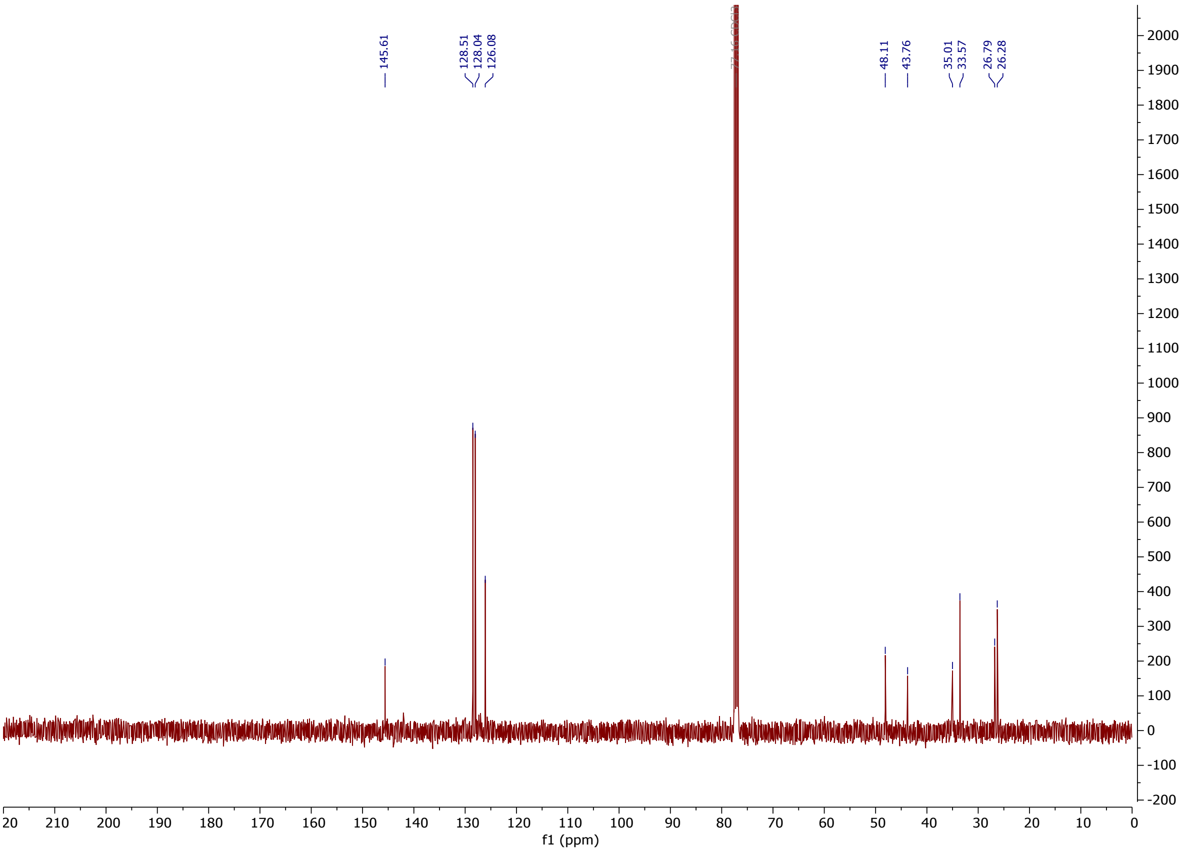

^{1}H NMR (400 MHz, $CDCl_3$) spectrum of **3c**

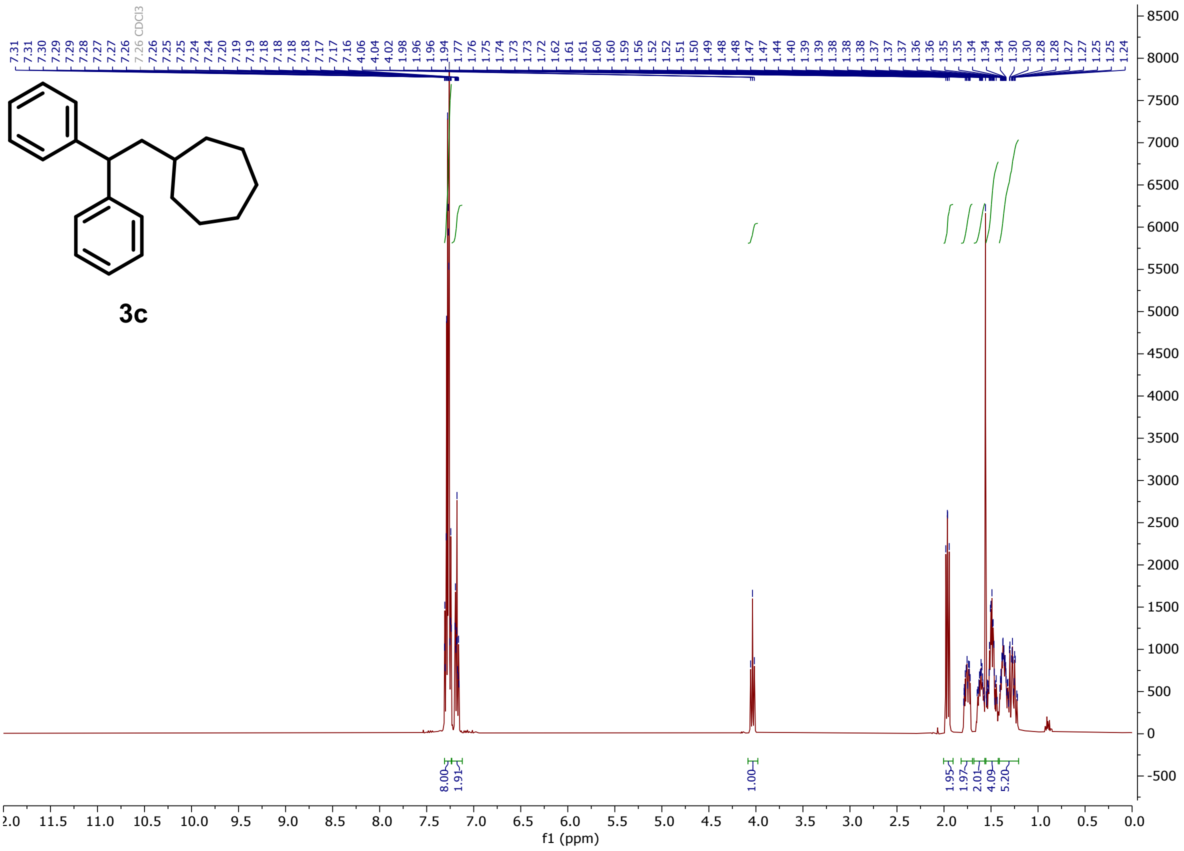

^{13}C{^{1}H} NMR (101 MHz, $CDCl_3$) spectrum of **3c**

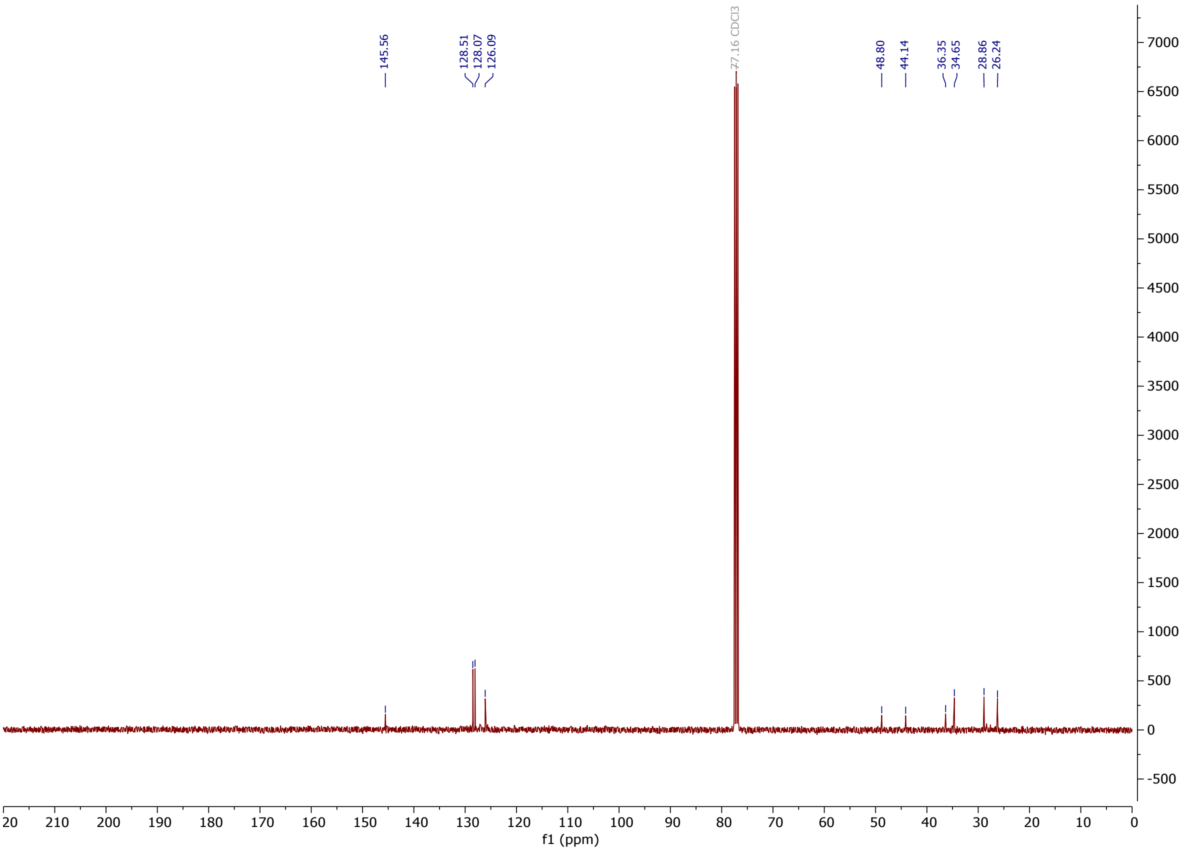

^{1}H NMR (400 MHz, CDCl$_3$) spectrum of **3d**

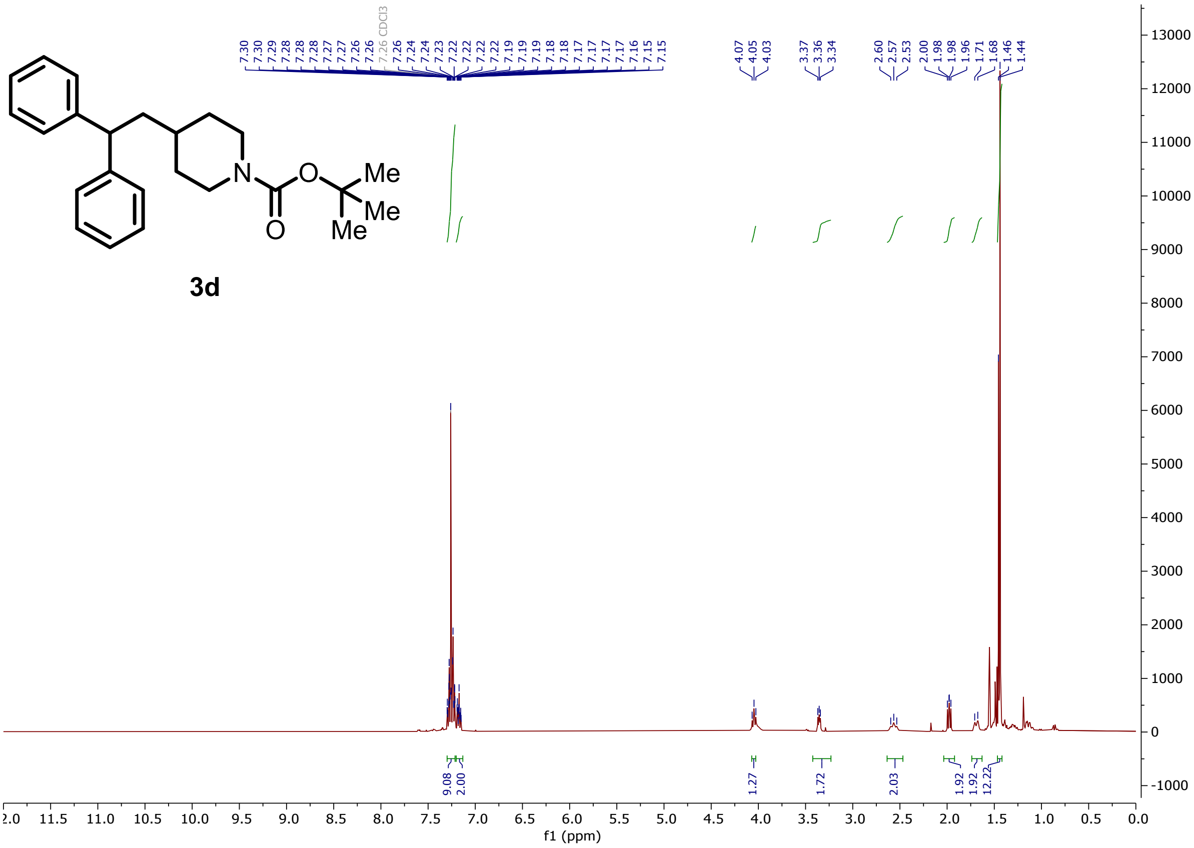

^{13}C{^{1}H} NMR (101 MHz, CDCl$_3$) spectrum of **3d**

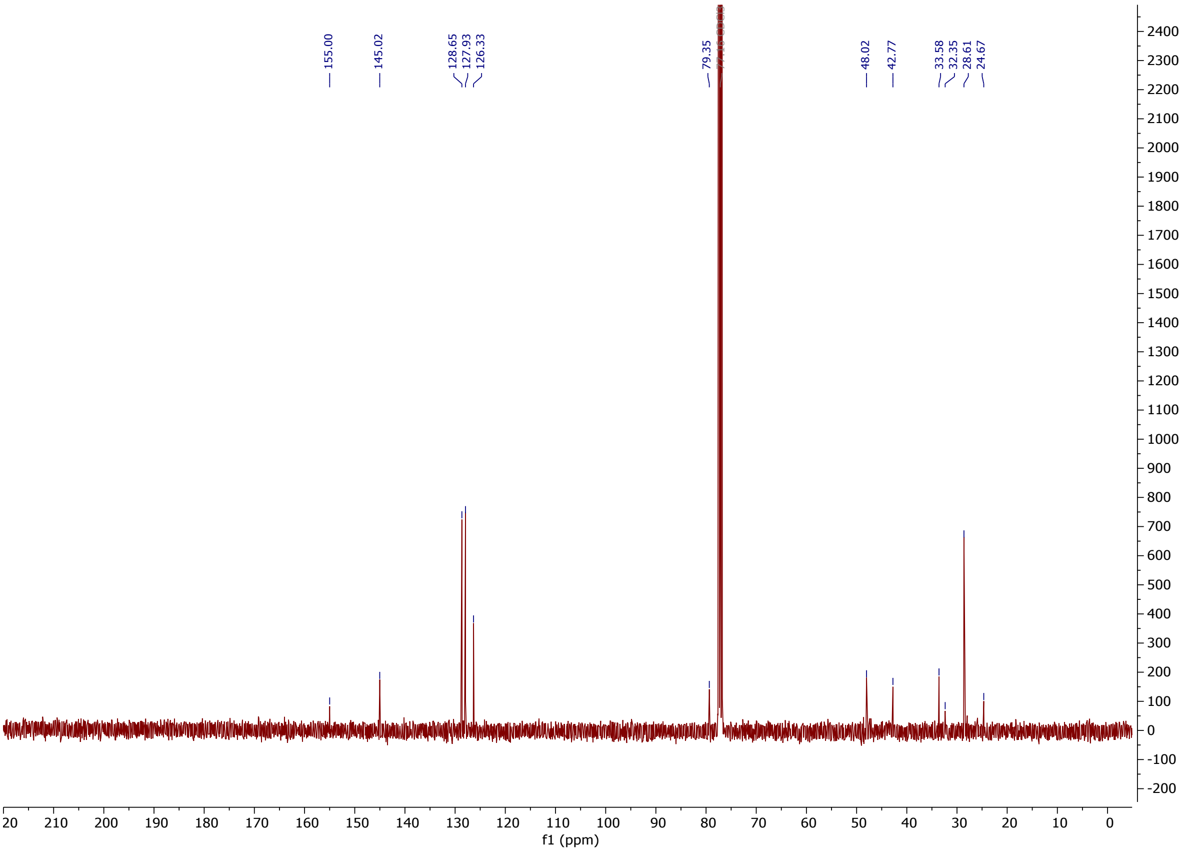

^1H NMR (400 MHz, $CDCl_3$) spectrum of **3e**

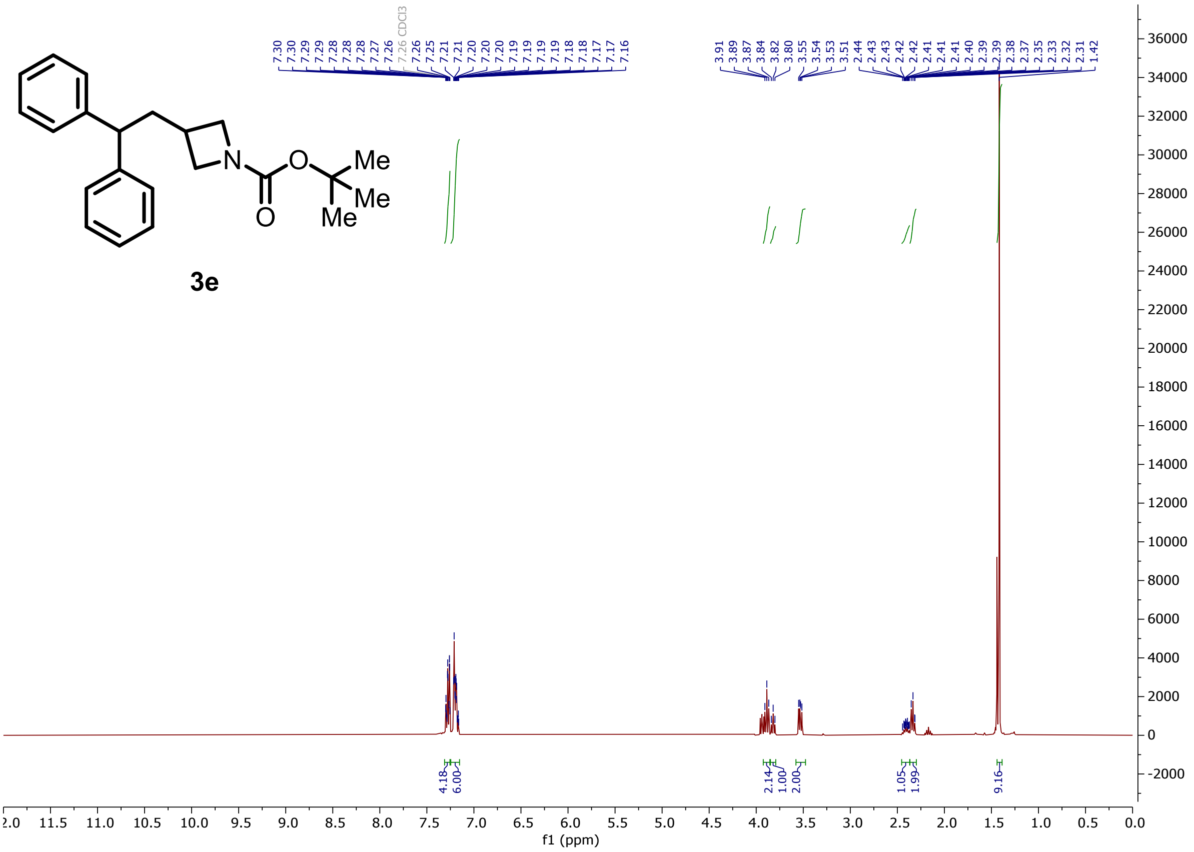

$^{13}C\{^1H\}$ NMR (101 MHz, $CDCl_3$) spectrum of **3e**

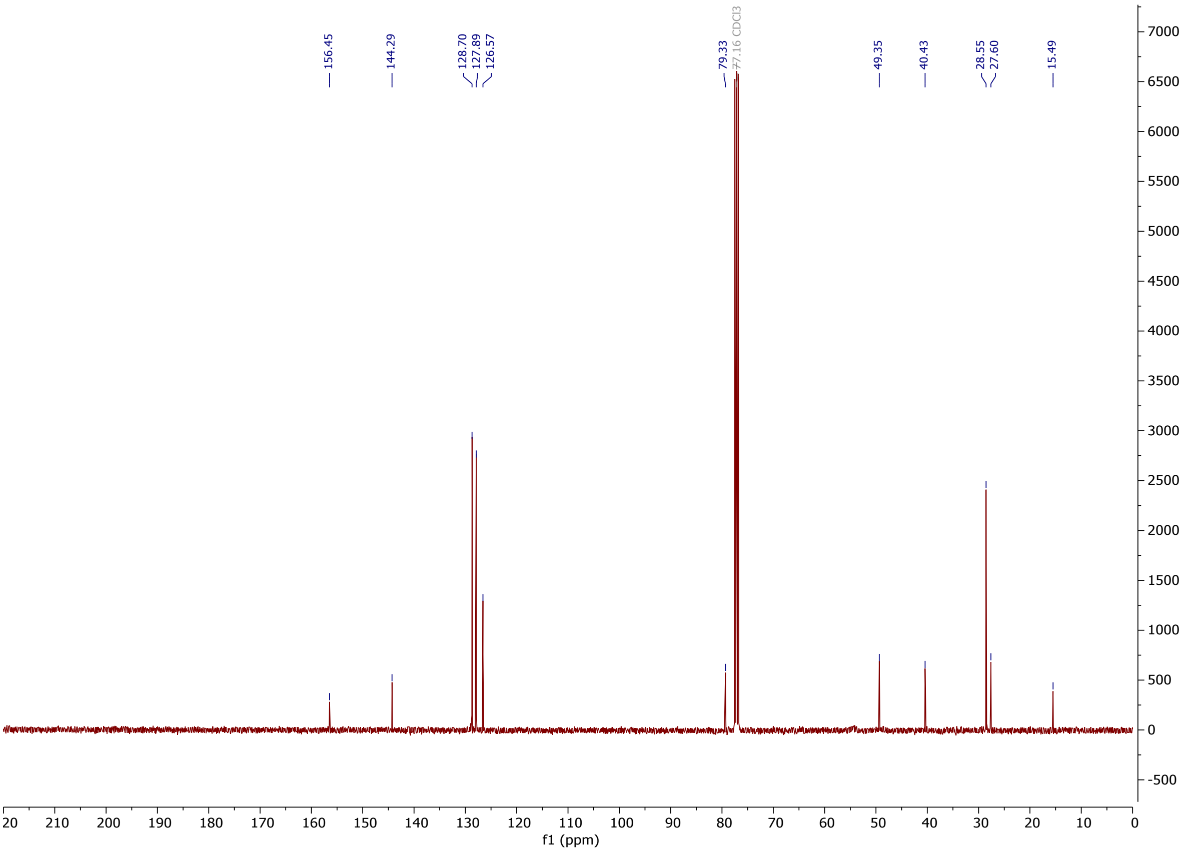

^1H NMR (400 MHz, $CDCl_3$) spectrum of **3f**

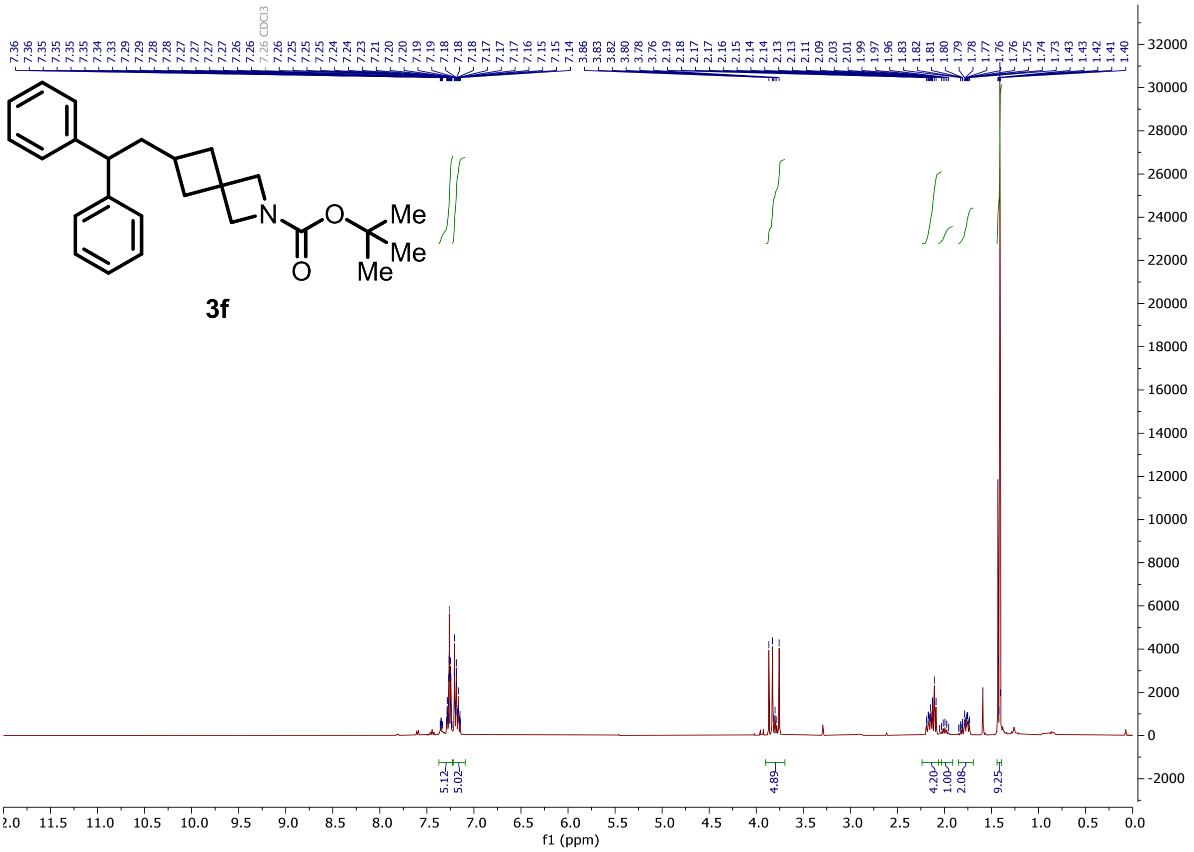

$^{13}C\{^1H\}$ NMR (101 MHz, $CDCl_3$) spectrum of **3f**

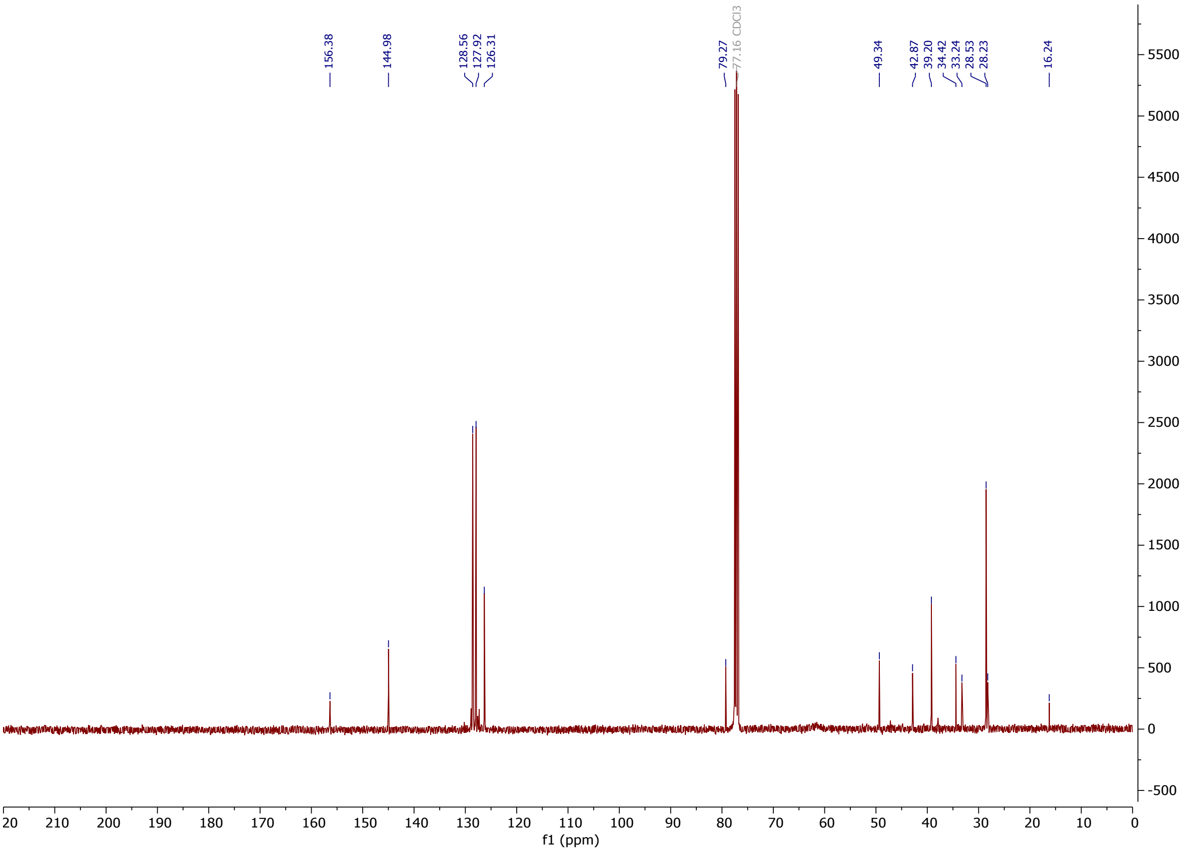

^{1}H NMR (400 MHz, $CDCl_3$) spectrum of **3g**

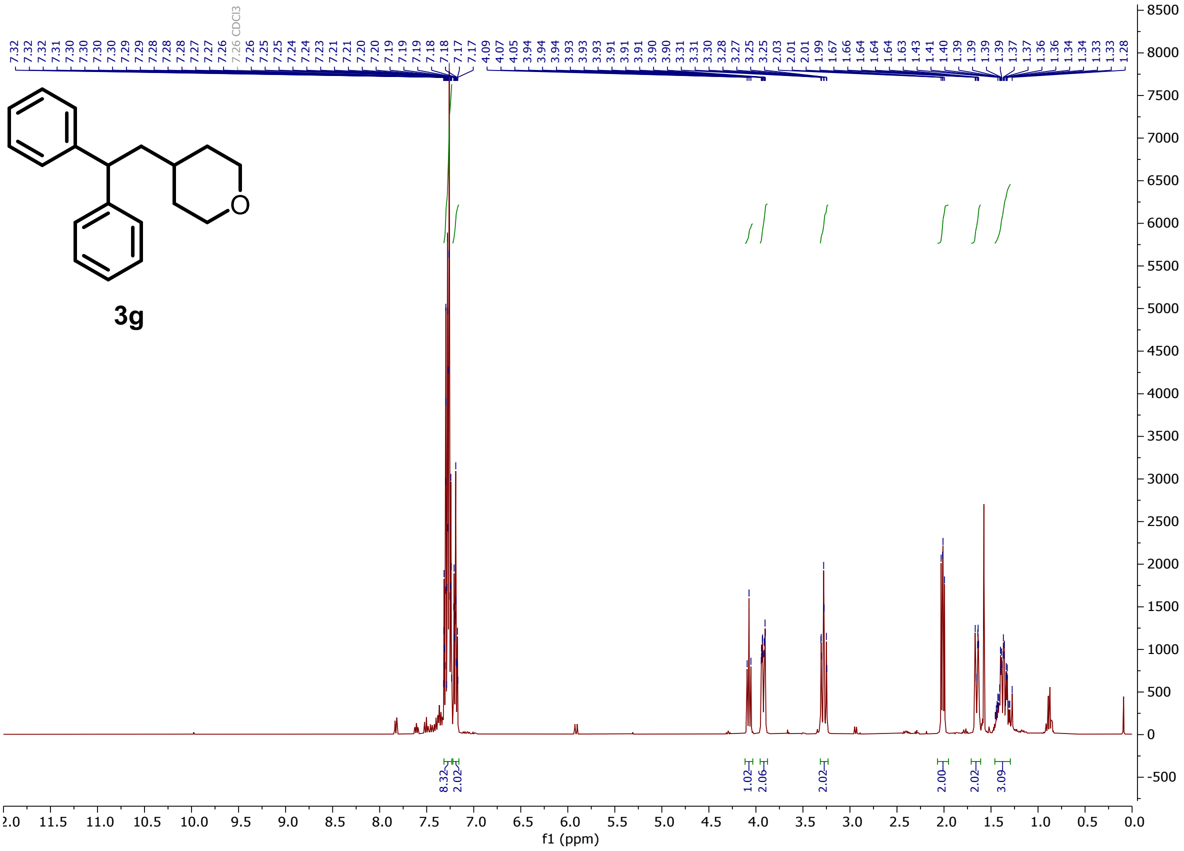

$^{13}C\{^{1}H\}$ NMR (101 MHz, $CDCl_3$) spectrum of **3g**

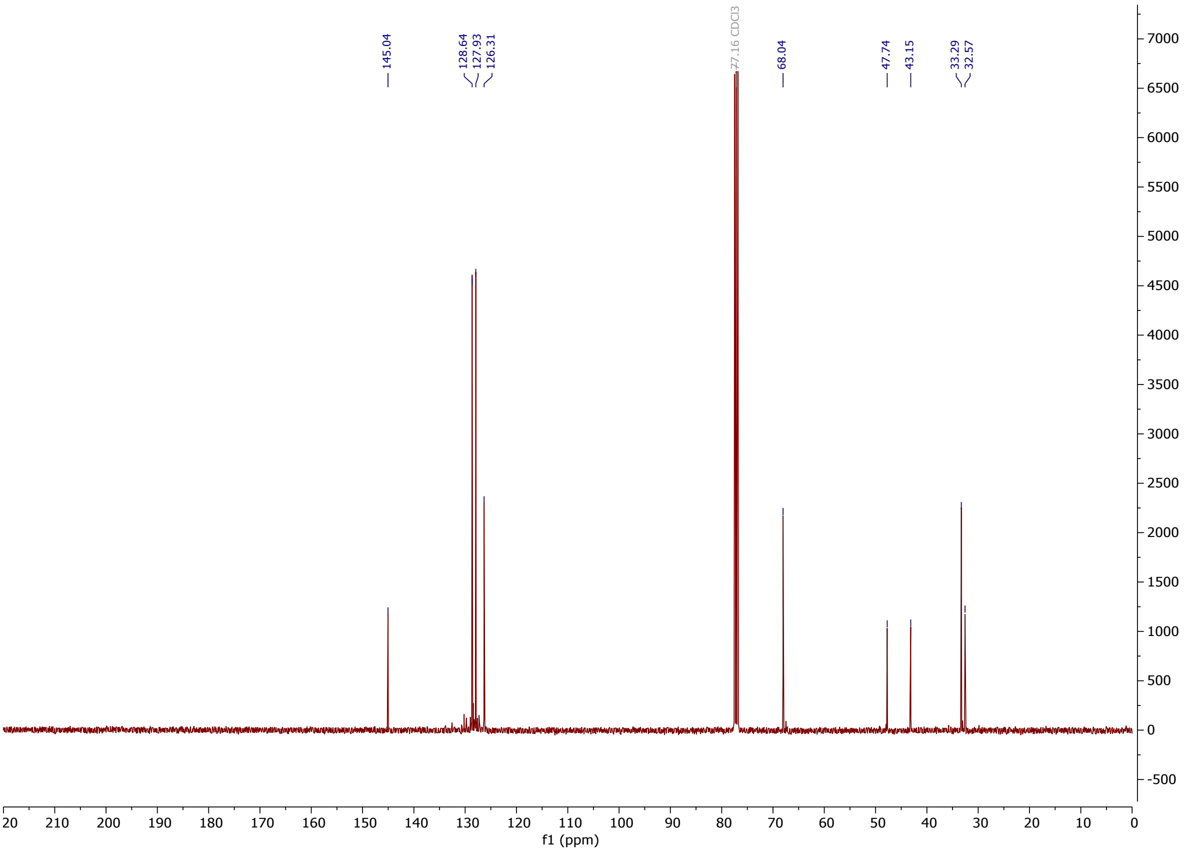

^{1}H NMR (400 MHz, $CDCl_3$) spectrum of **3h**

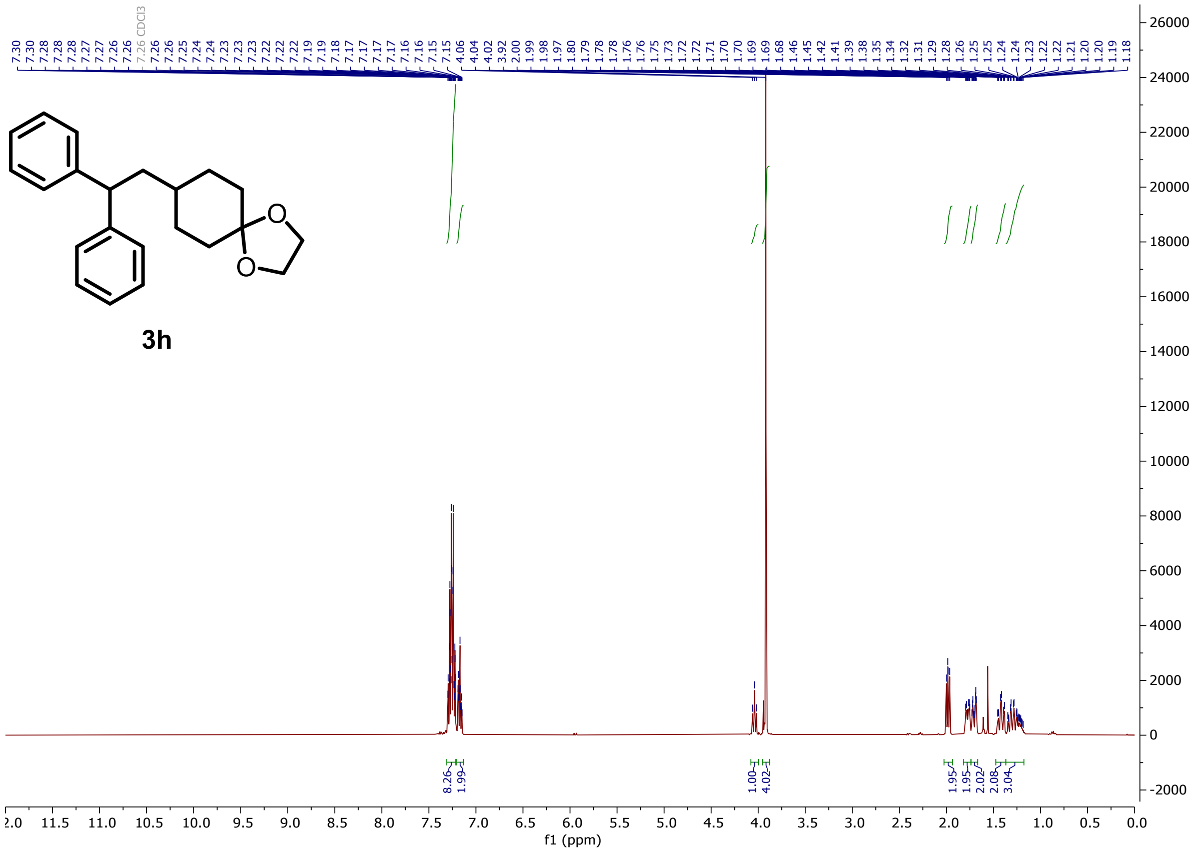

$^{13}C\{^{1}H\}$ NMR (101 MHz, $CDCl_3$) spectrum of **3h**

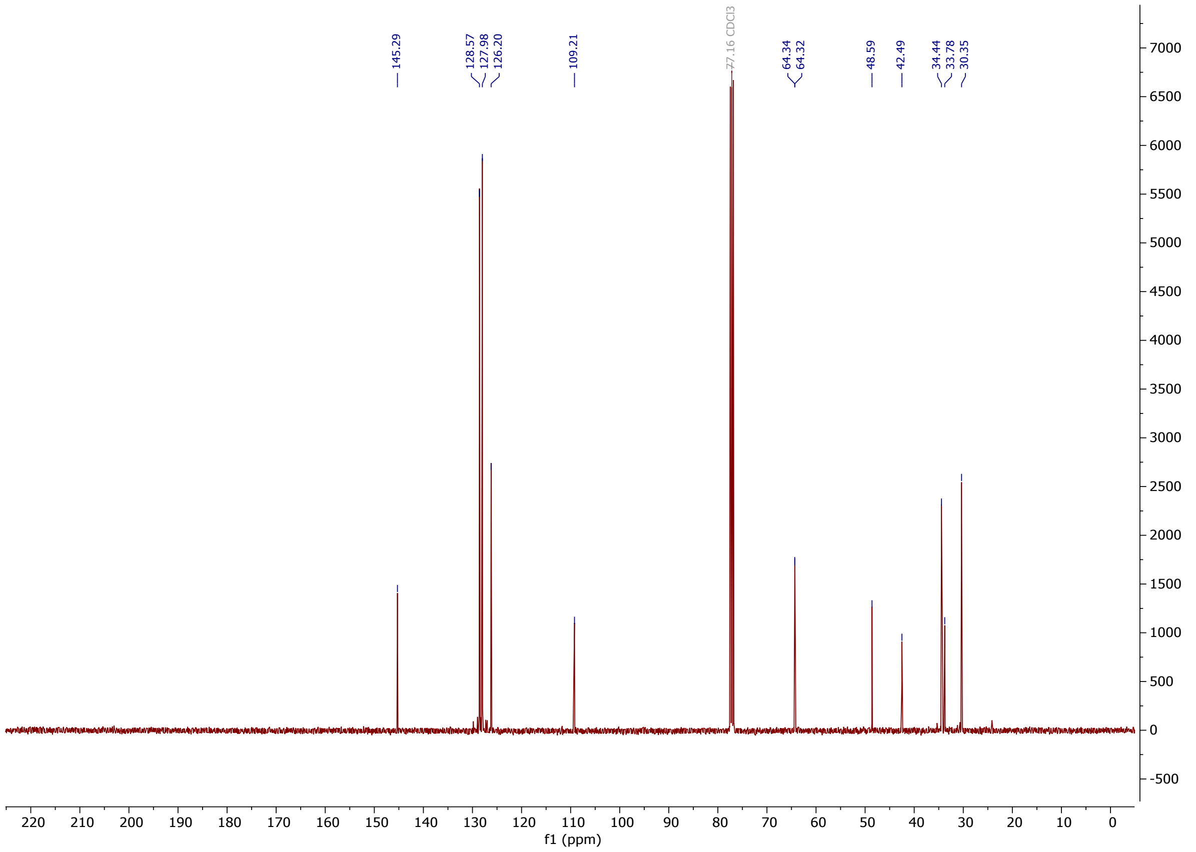

^{1}H NMR (400 MHz, $CDCl_3$) spectrum of **3i**

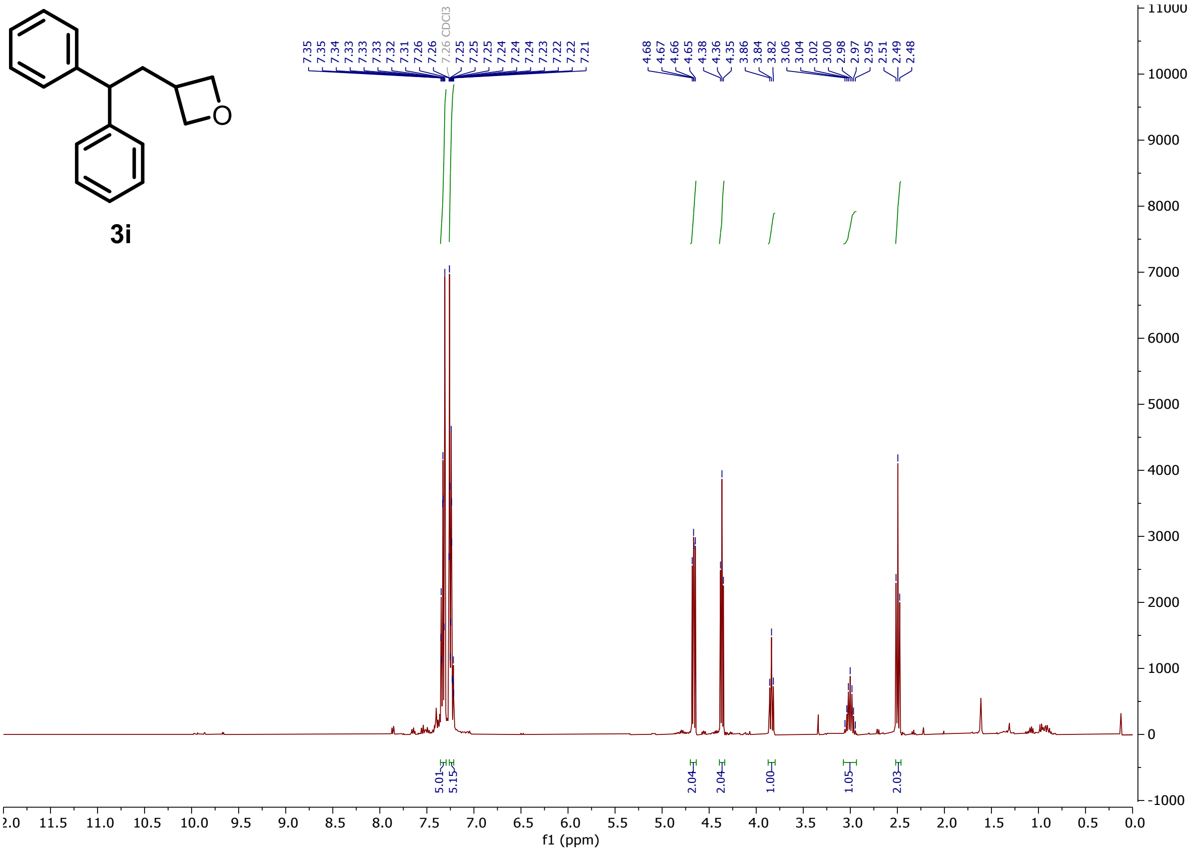

$^{13}C\{^{1}H\}$ NMR (101 MHz, $CDCl_3$) spectrum of **3i**

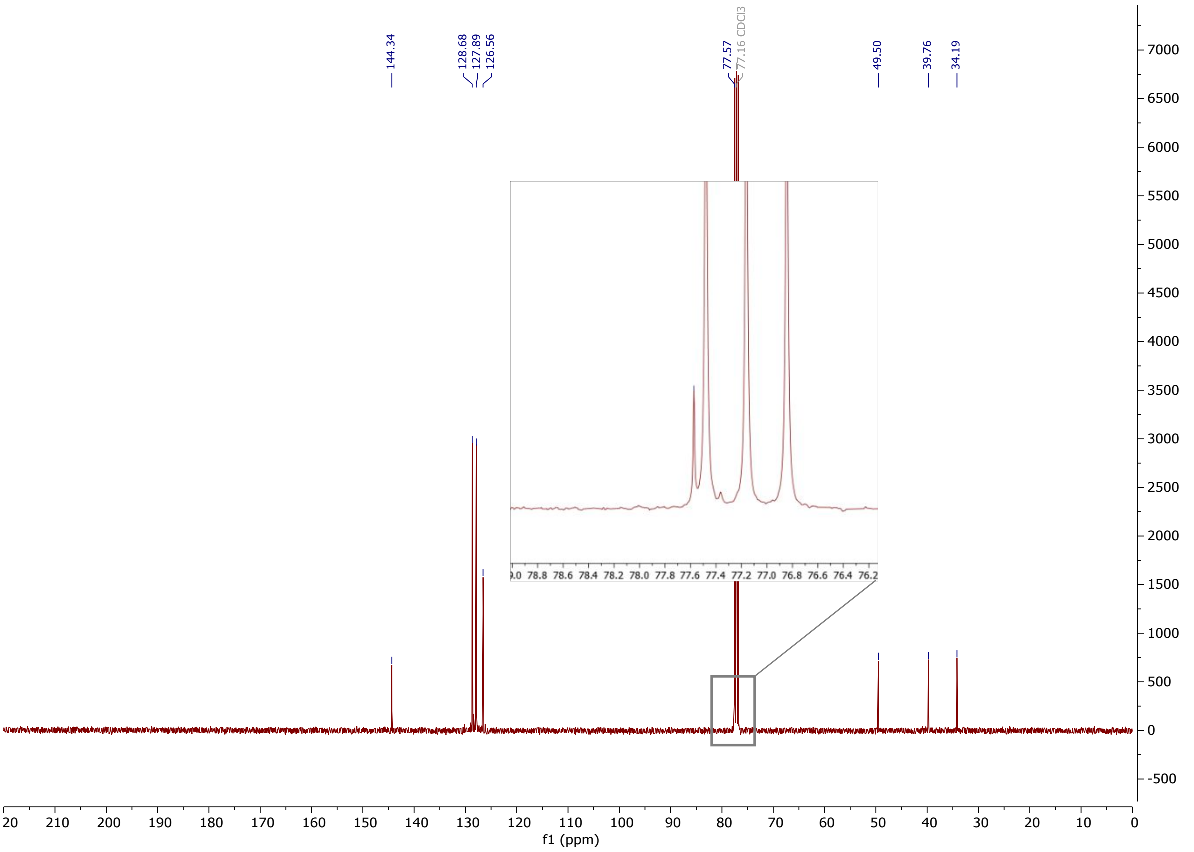

^1H NMR (400 MHz, $CDCl_3$) spectrum of **3j**

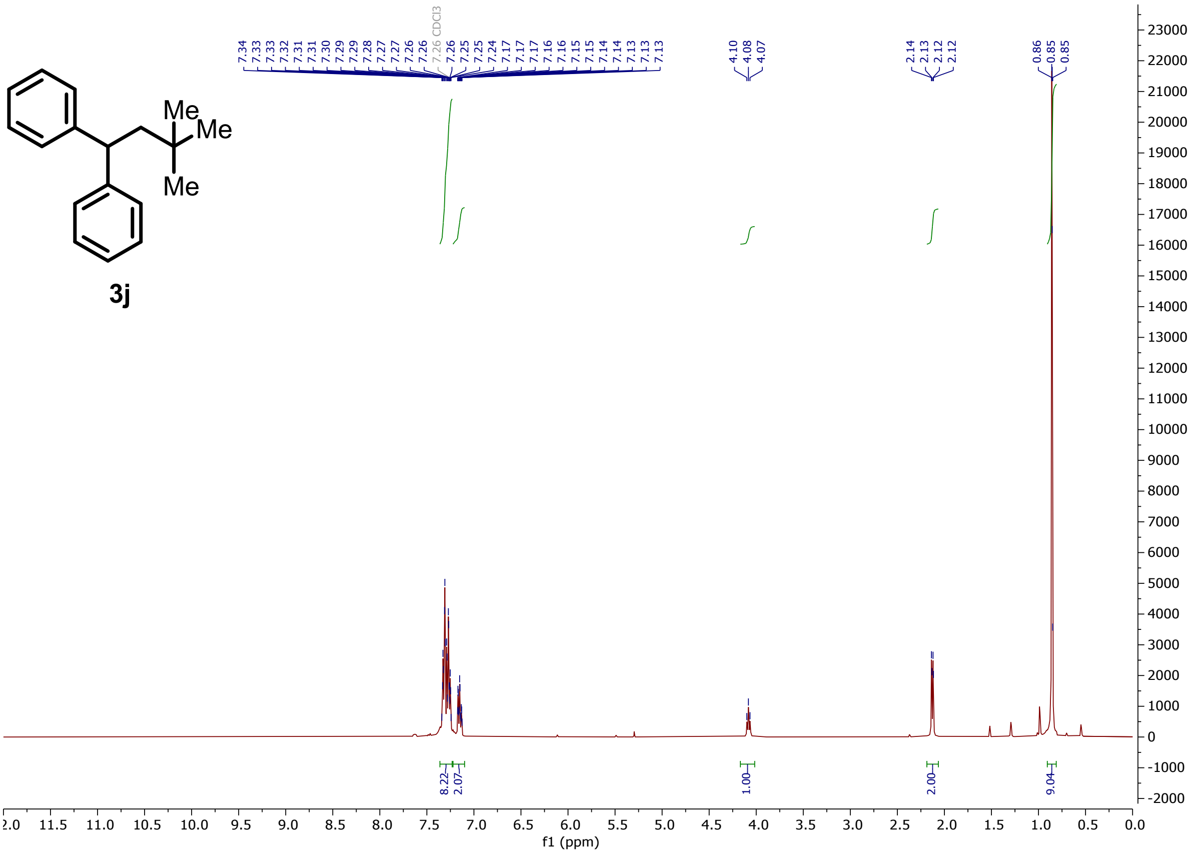

$^{13}C\{^1H\}$ NMR (101 MHz, $CDCl_3$) spectrum of **3j**

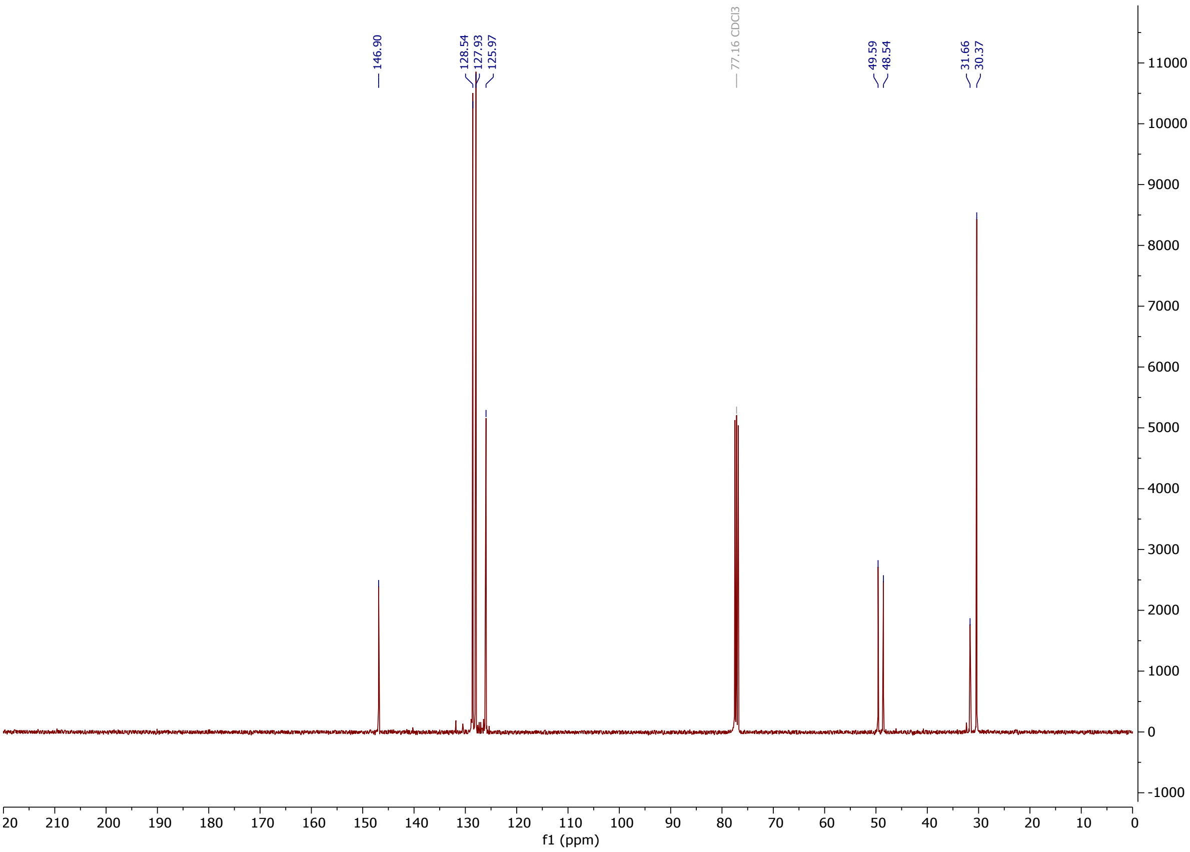

^{1}H NMR (400 MHz, $CDCl_3$) spectrum of **3k**

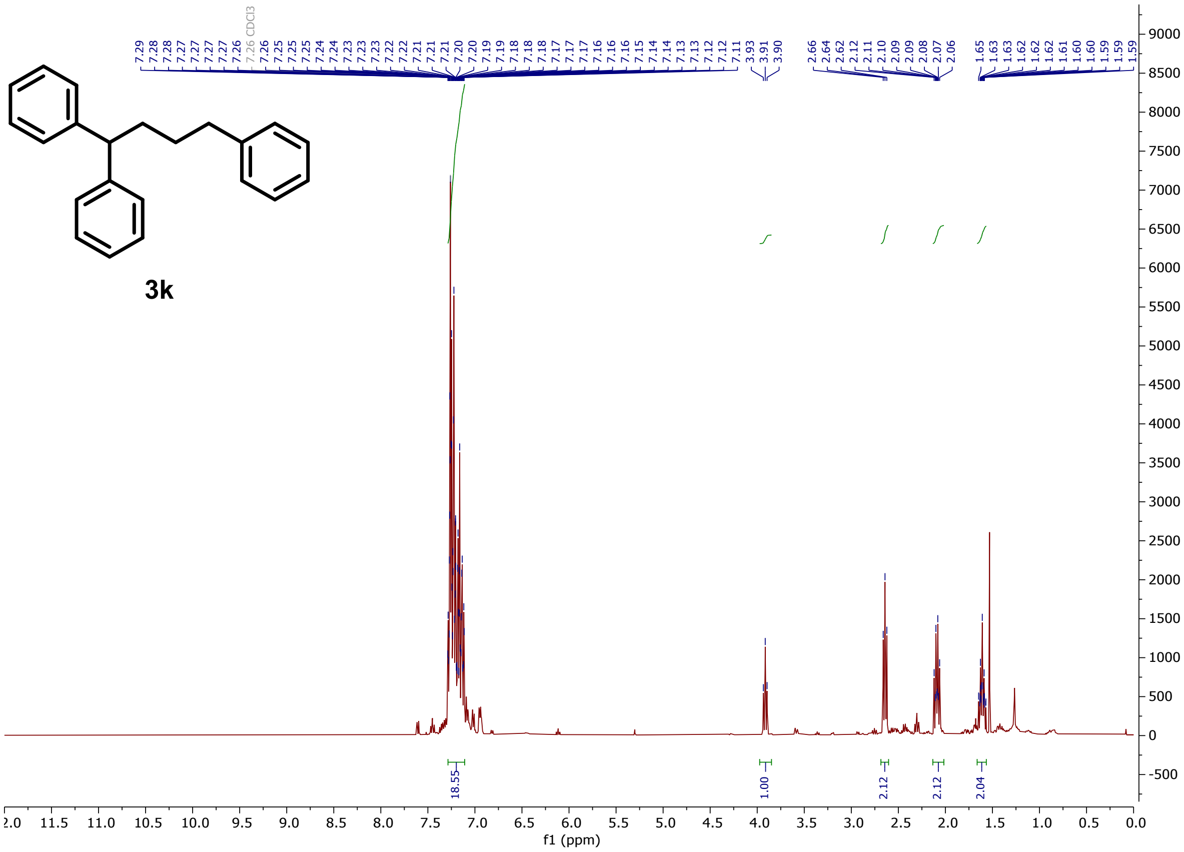

$^{13}C\{^{1}H\}$ NMR (101 MHz, $CDCl_3$) spectrum of **3k**

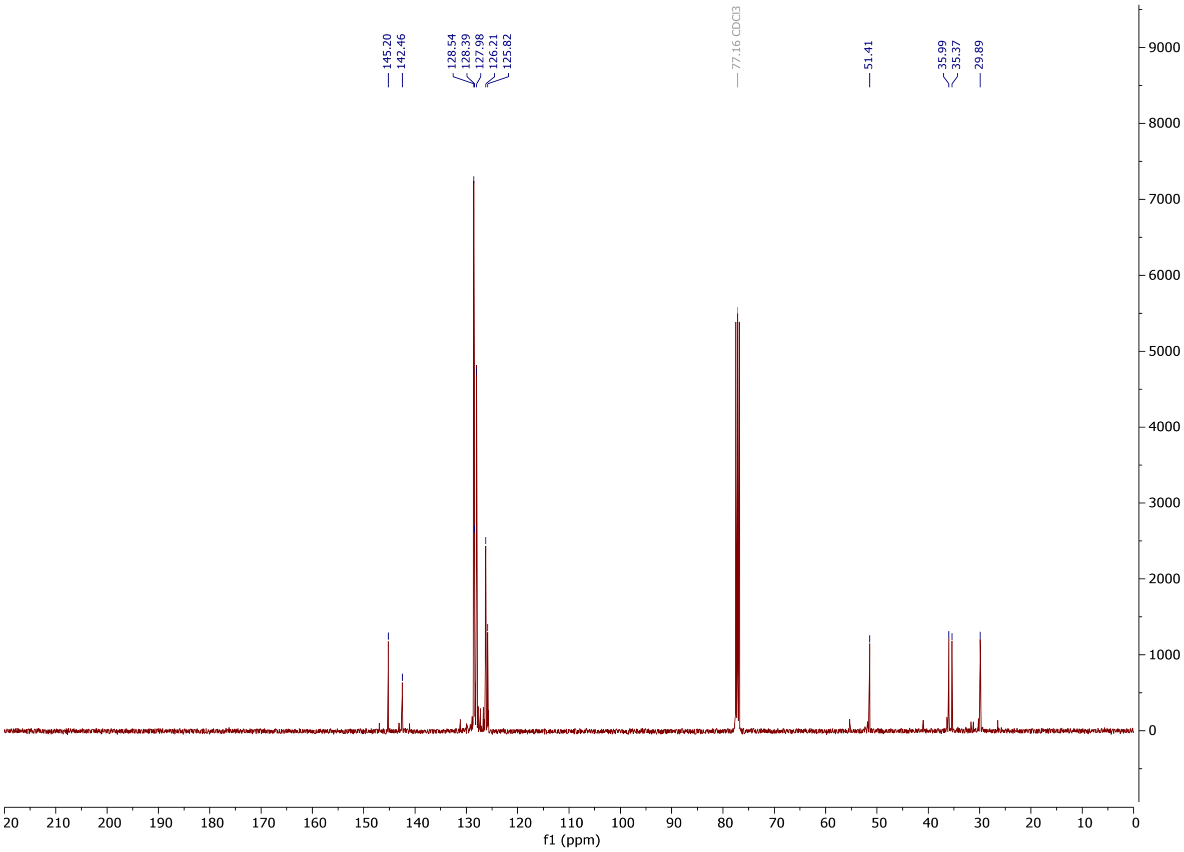

^{1}H NMR (400 MHz, $CDCl_3$) spectrum of **3l**

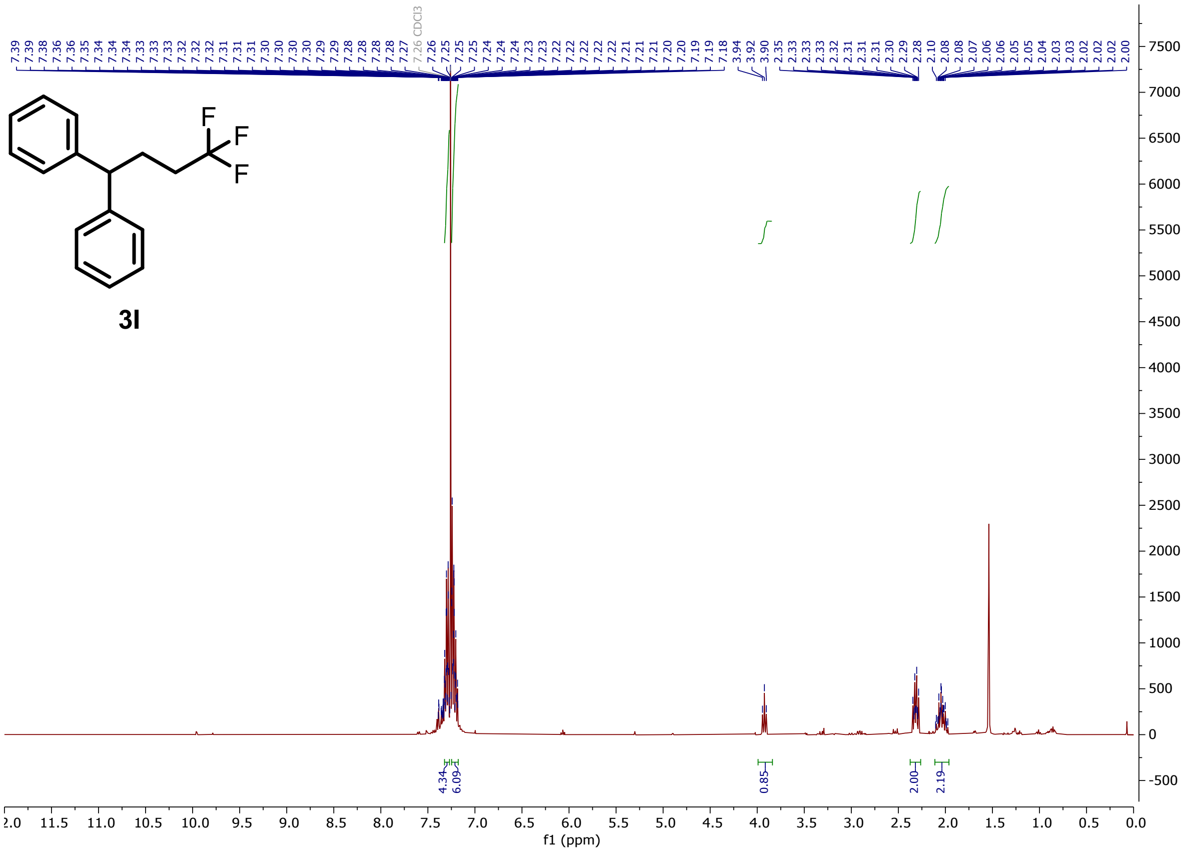

$^{13}C\{^{1}H\}$ NMR (101 MHz, $CDCl_3$) spectrum of **3l**

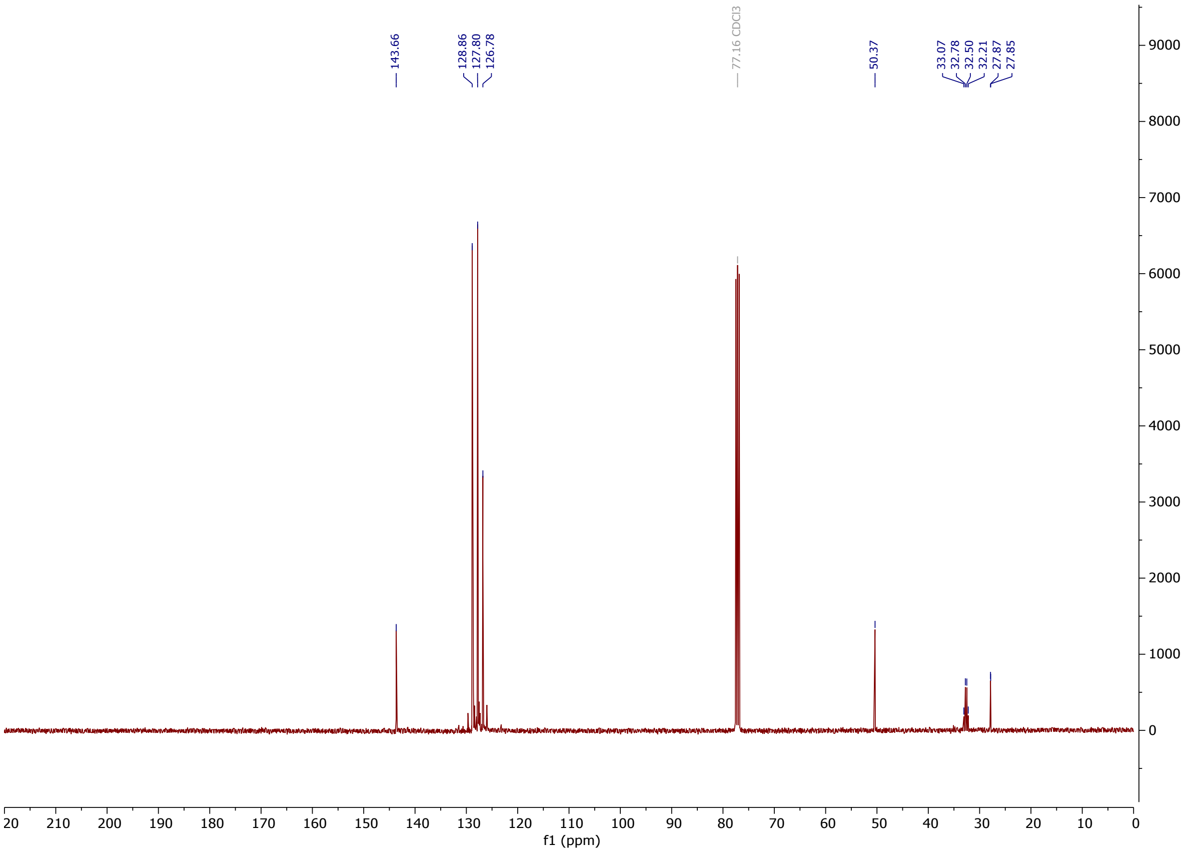

$^{19}F\{^{1}H\}$ NMR (376 MHz, $CDCl_3$) spectrum of **3l**

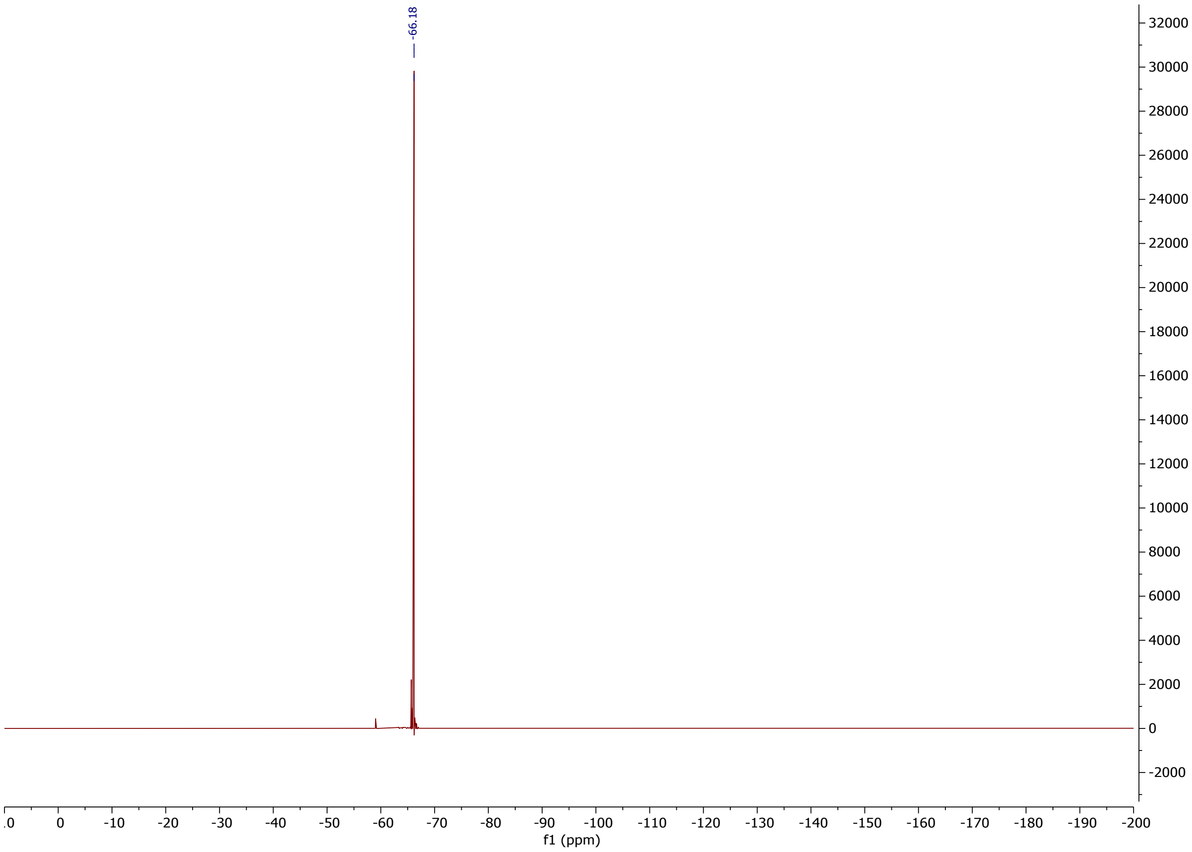

^{1}H NMR (400 MHz, $CDCl_3$) spectrum of **3m**

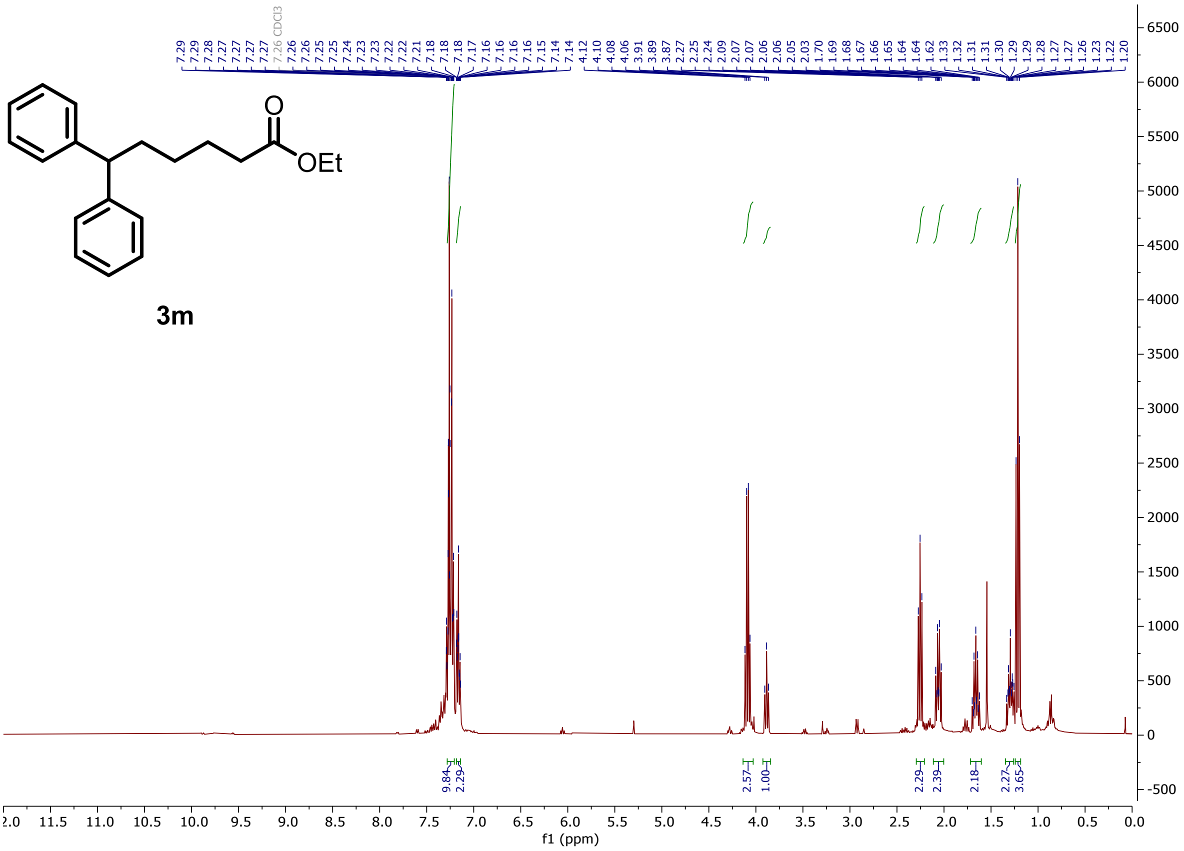

$^{13}C\{^{1}H\}$ NMR (101 MHz, $CDCl_3$) spectrum of **3m**

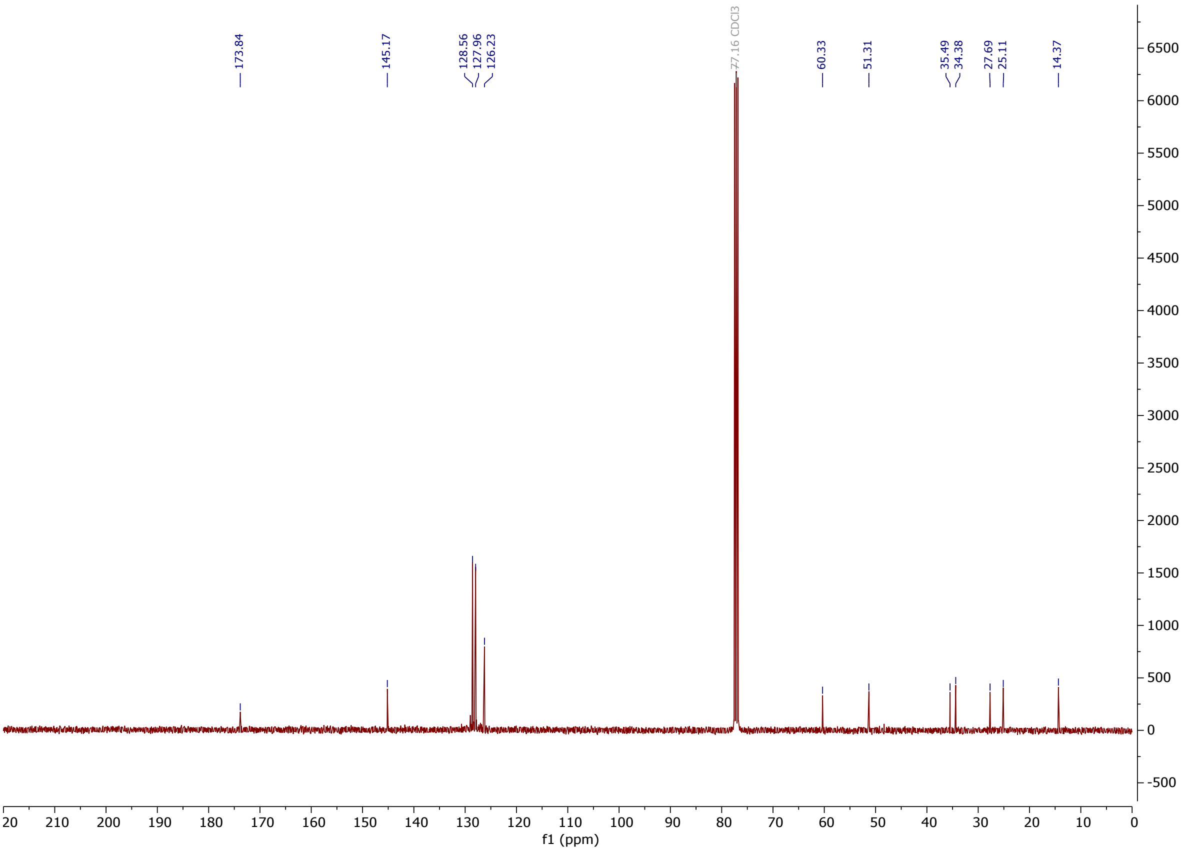

^{1}H NMR (400 MHz, $CDCl_3$) spectrum of **3n**

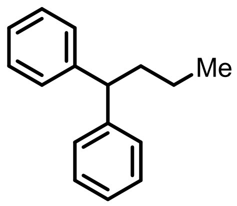

3n

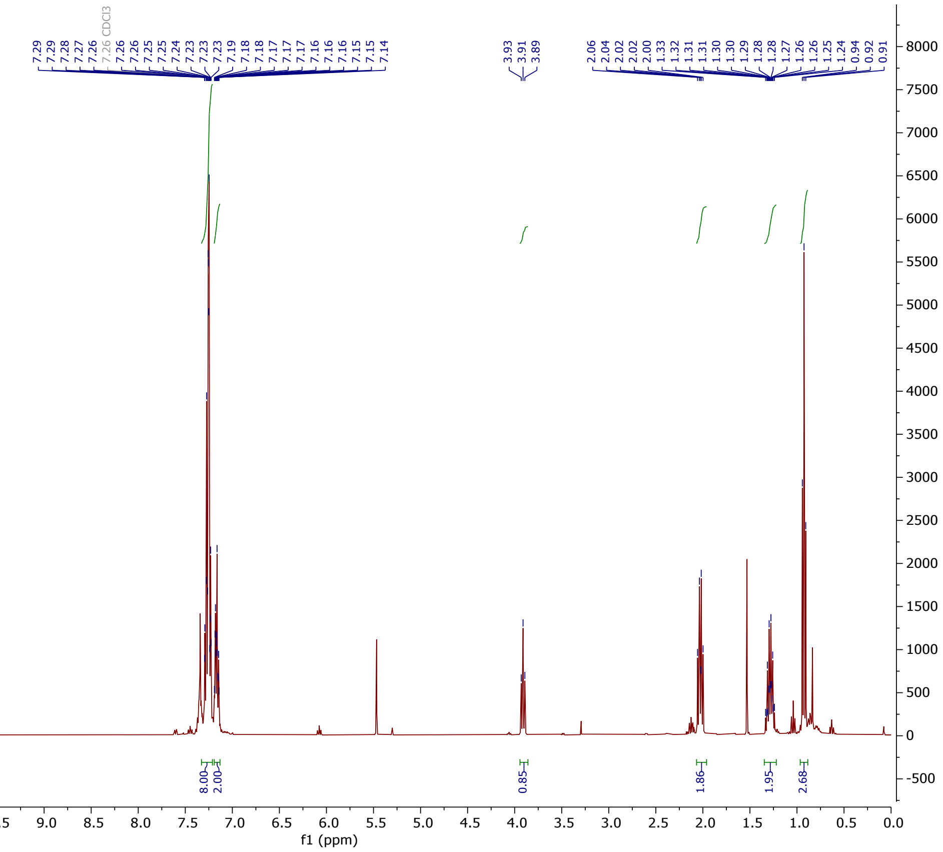

$^{13}C\{^{1}H\}$ NMR (101 MHz, $CDCl_3$) spectrum of **3n**

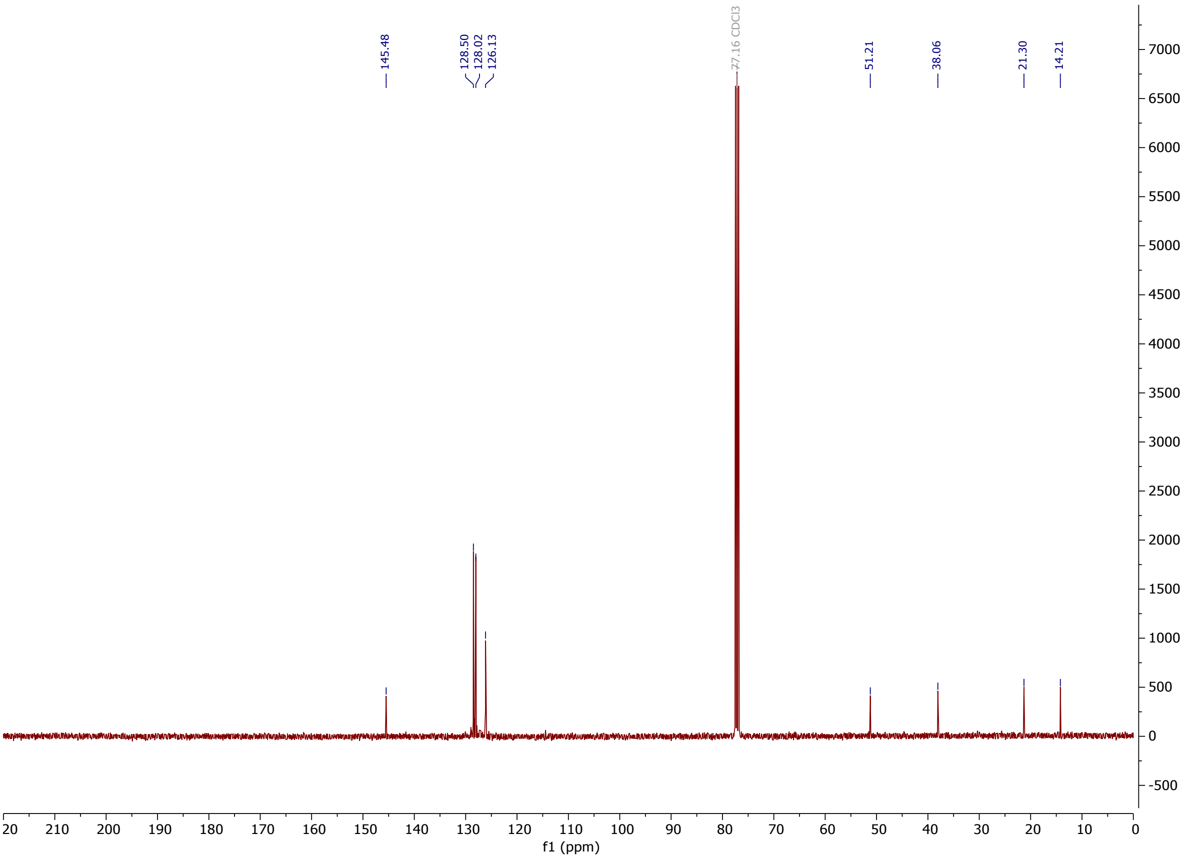

^{1}H NMR (400 MHz, $CDCl_3$) spectrum of **3o**

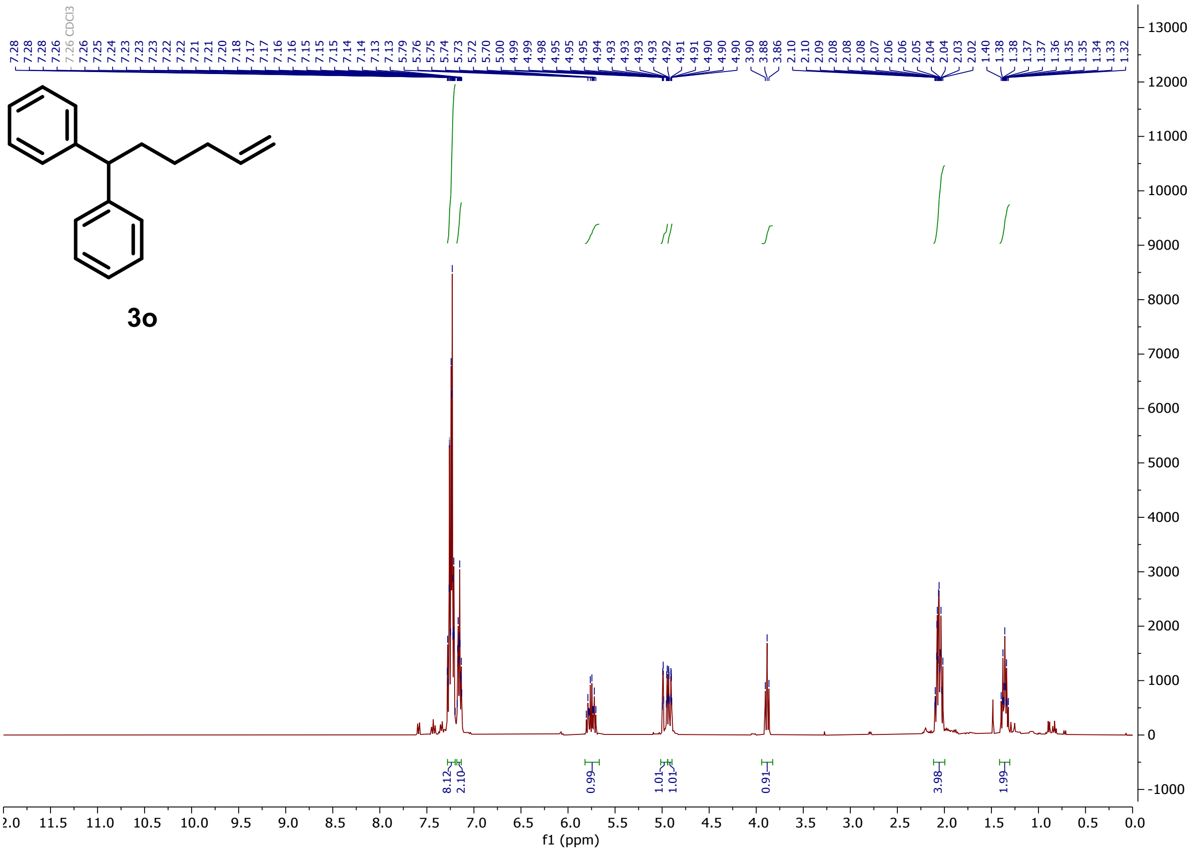

^{13}C{^{1}H} NMR (101 MHz, $CDCl_3$) spectrum of **3o**

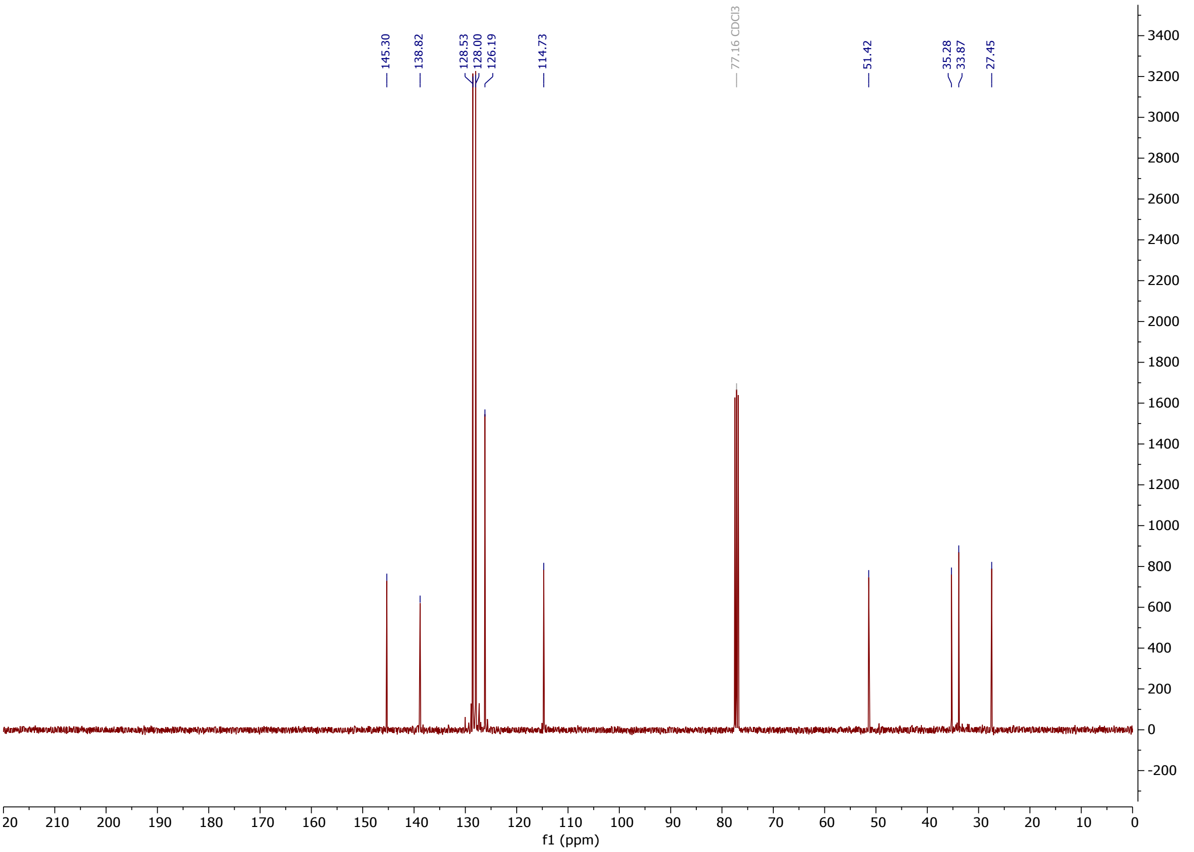

^{1}H NMR (400 MHz, $CDCl_3$) spectrum of **3p**

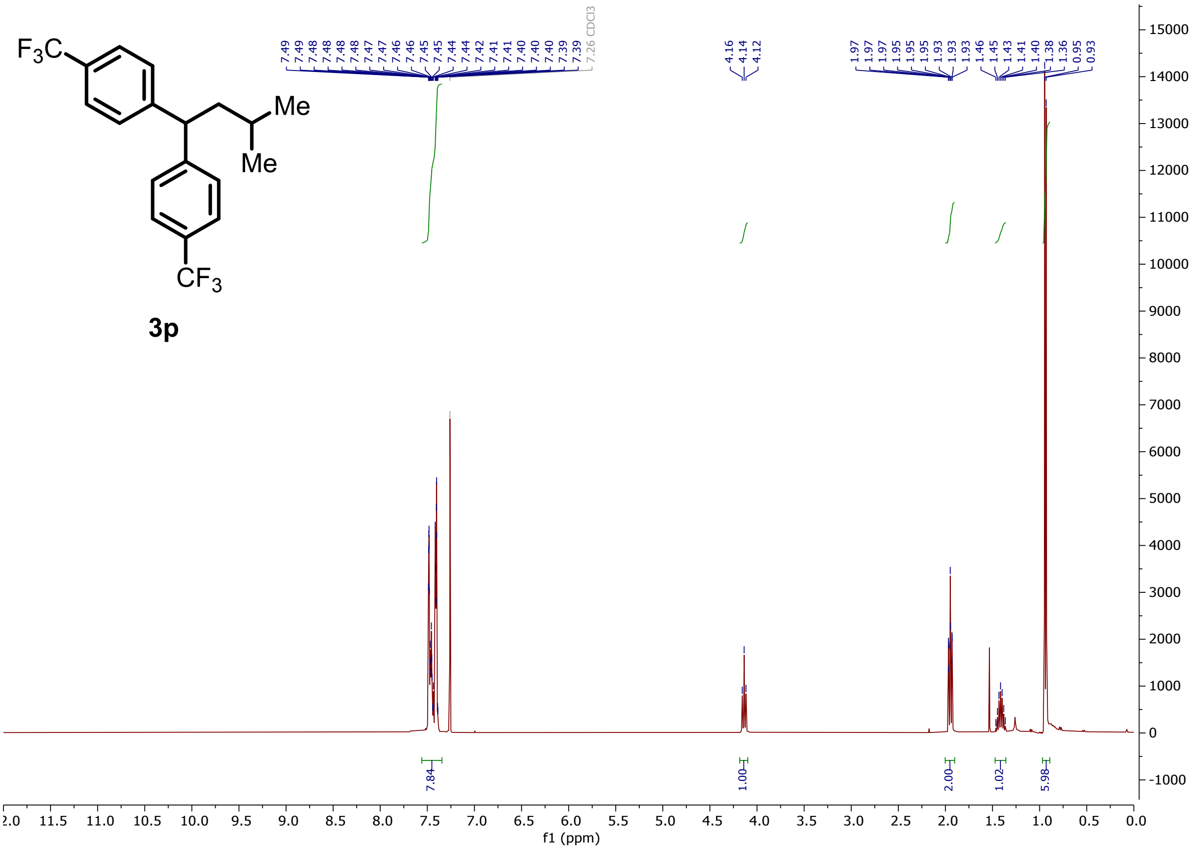

$^{13}C\{^{1}H\}$ NMR (101 MHz, $CDCl_3$) spectrum of **3p**

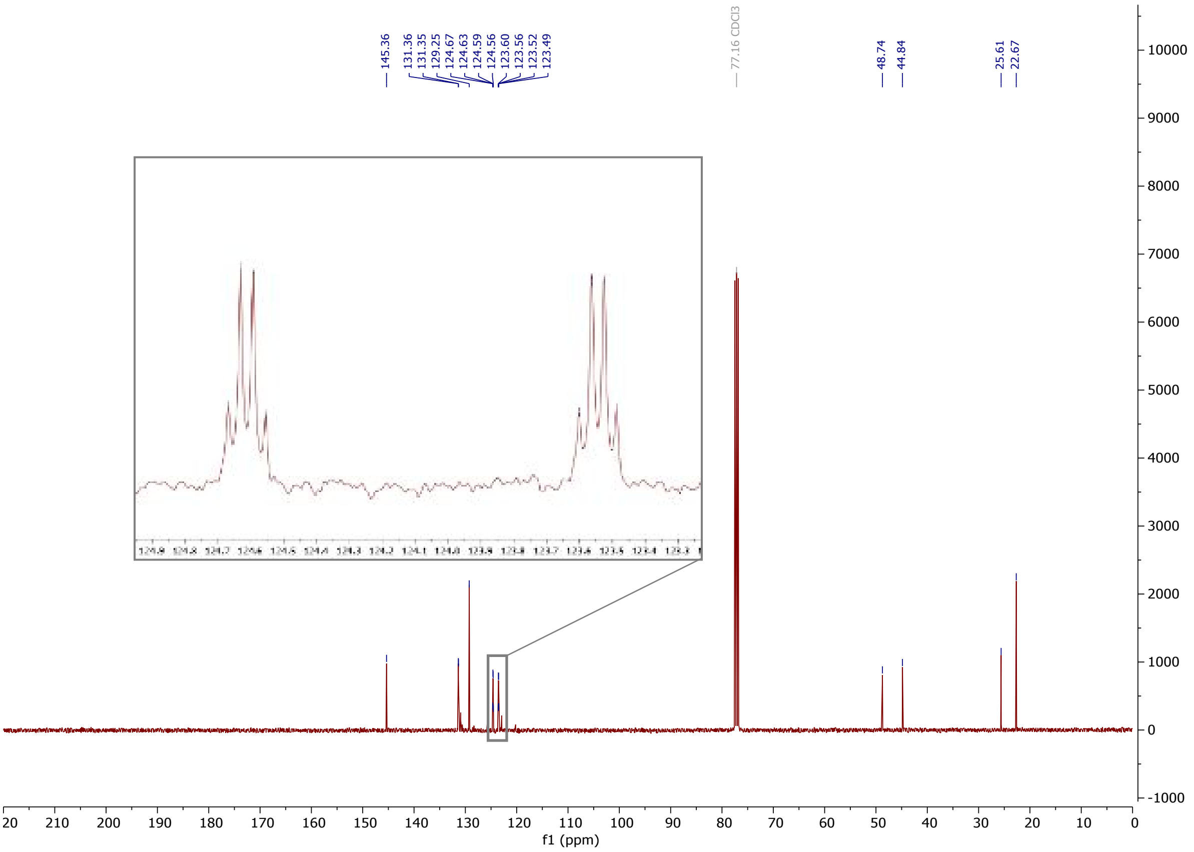

$^{19}F\{^{1}H\}$ NMR (376 MHz, $CDCl_3$) spectrum of **3p**

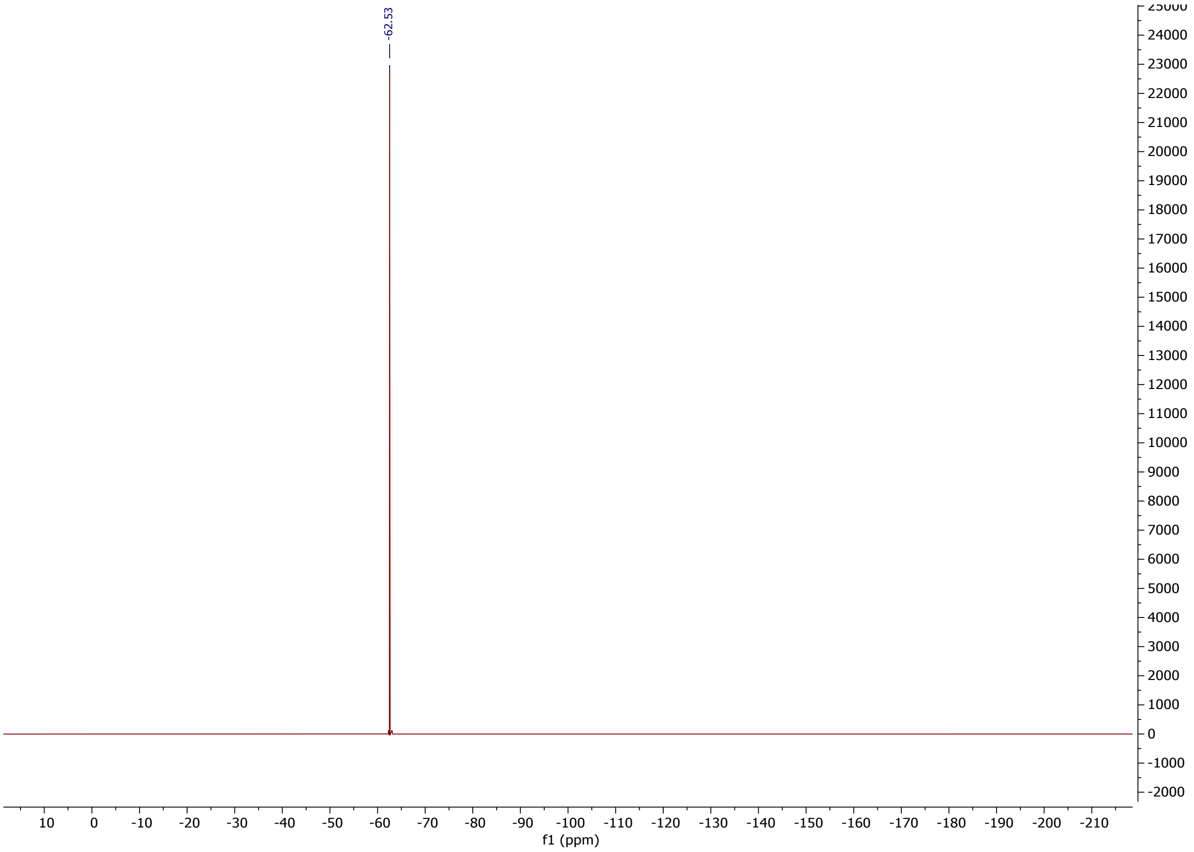

^{1}H NMR (400 MHz, $CDCl_3$) spectrum of **3q**

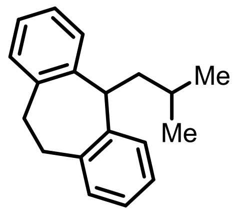

3q

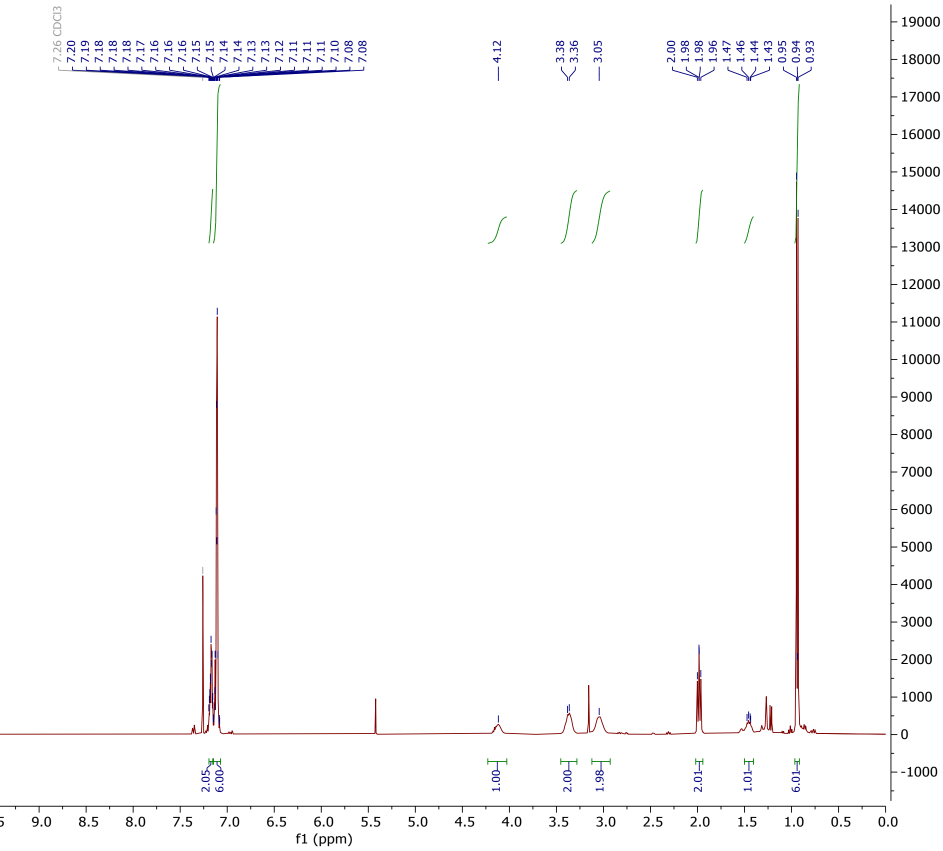

$^{13}C\{^{1}H\}$ NMR (101 MHz, $CDCl_3$) spectrum of **3q**

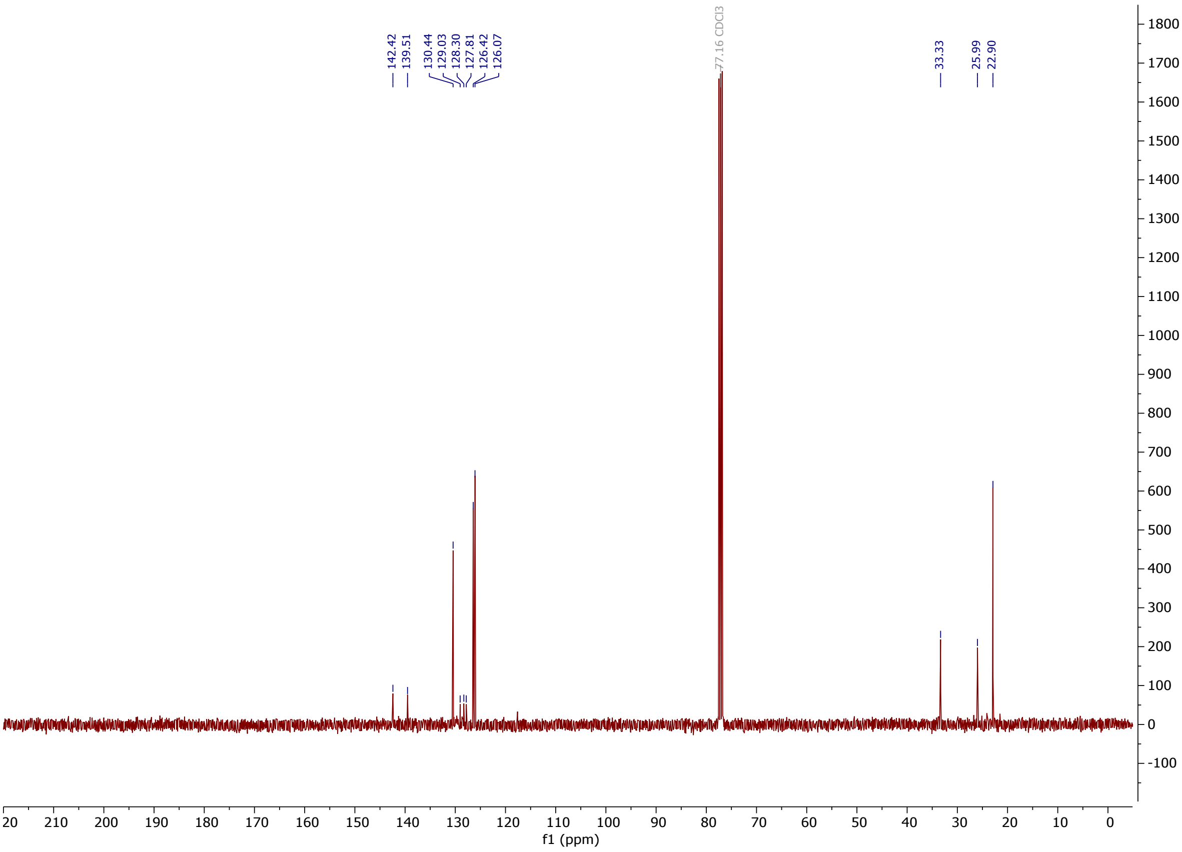

^{1}H NMR (400 MHz, $CDCl_3$) spectrum of **3q-b**

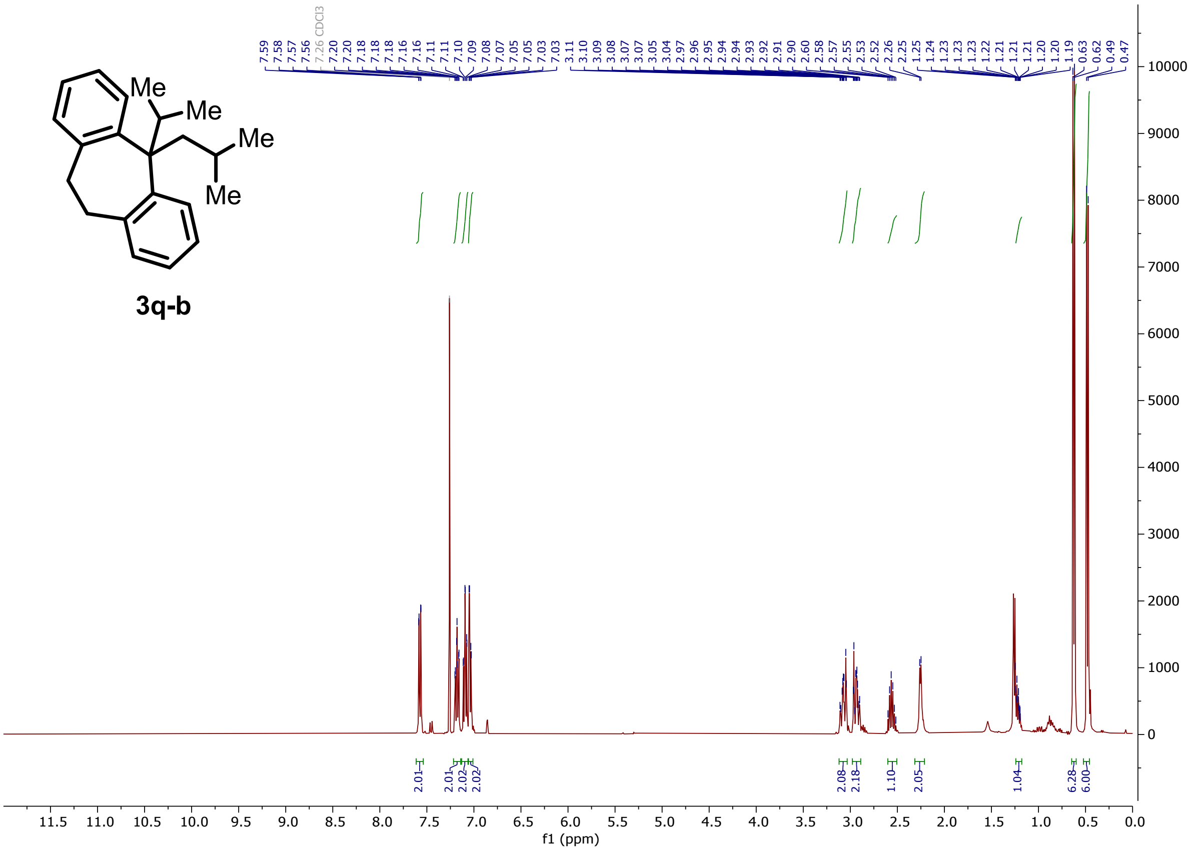

^{13}C{^{1}H} NMR (101 MHz, $CDCl_3$) spectrum of **3q-b**

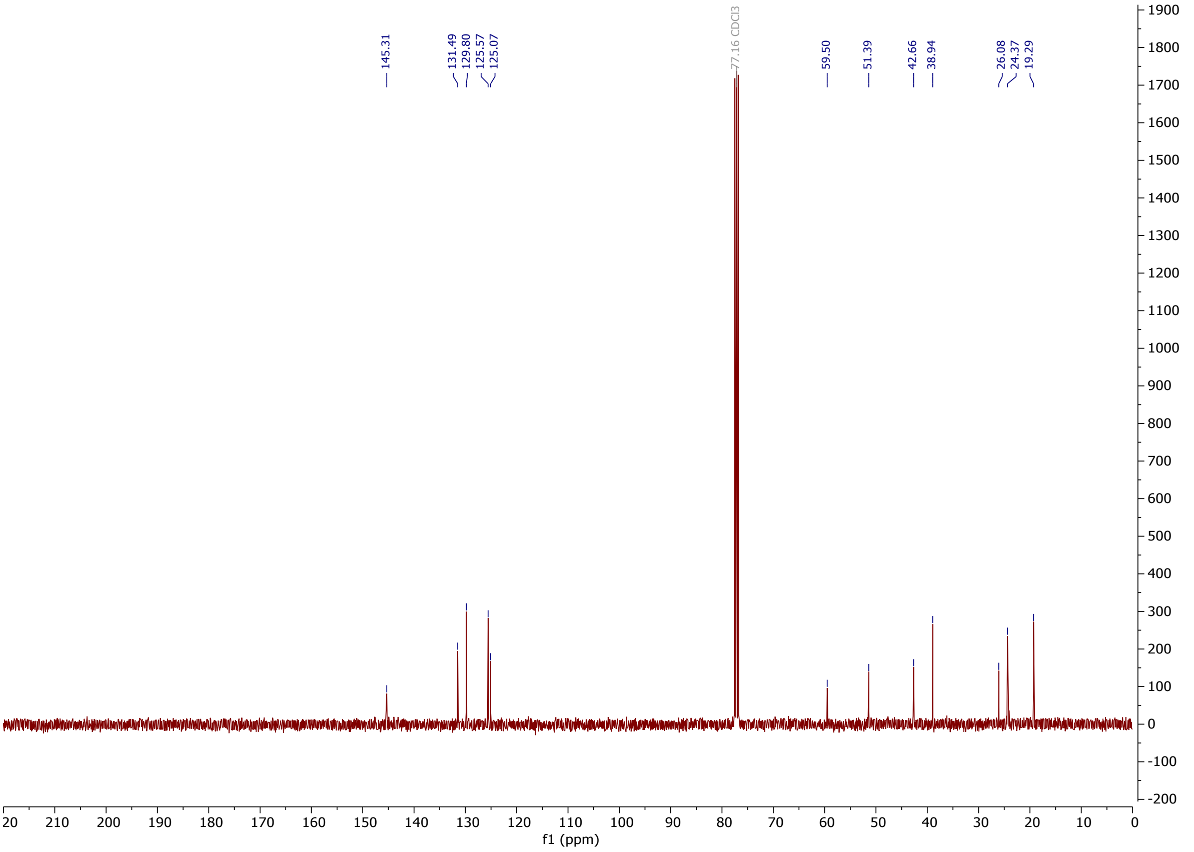

^{1}H NMR (400 MHz, $CDCl_3$) spectrum of **3r**

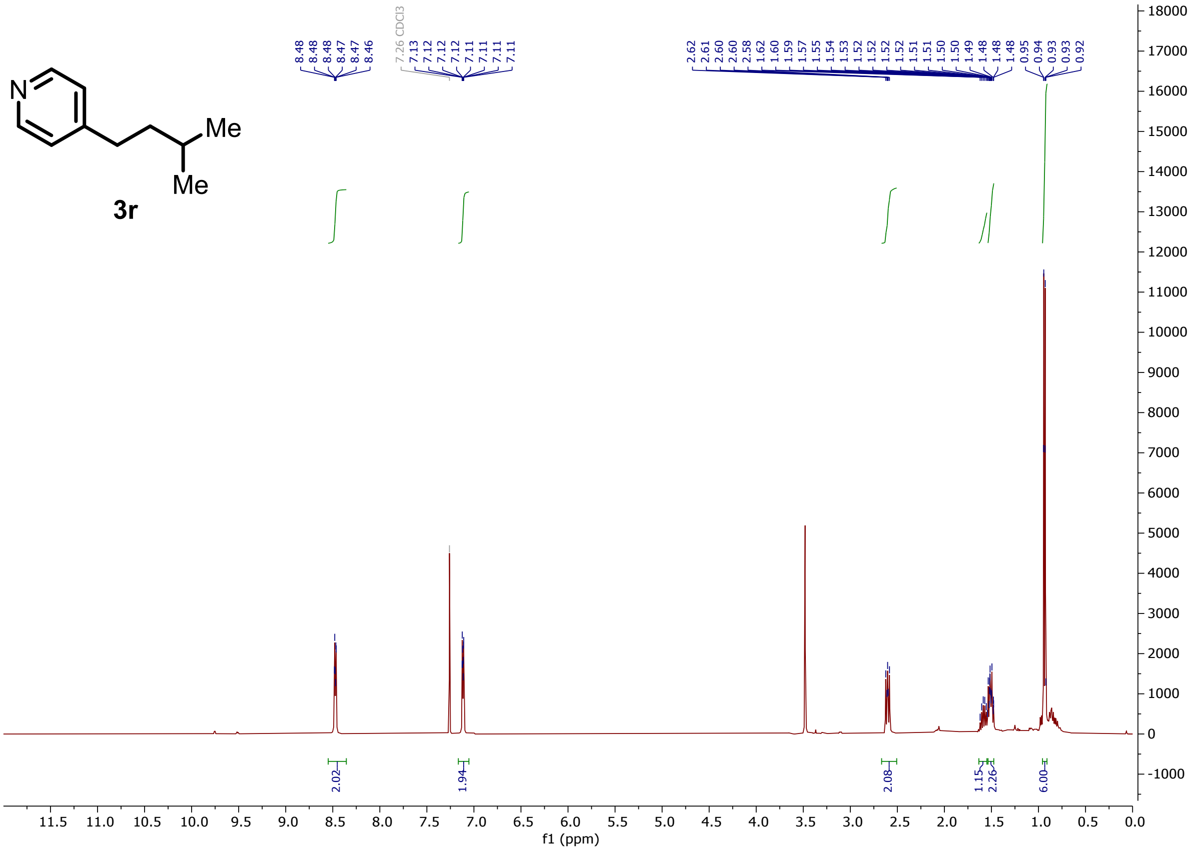

^{13}C{^{1}H} NMR (101 MHz, $CDCl_3$) spectrum of **3r**

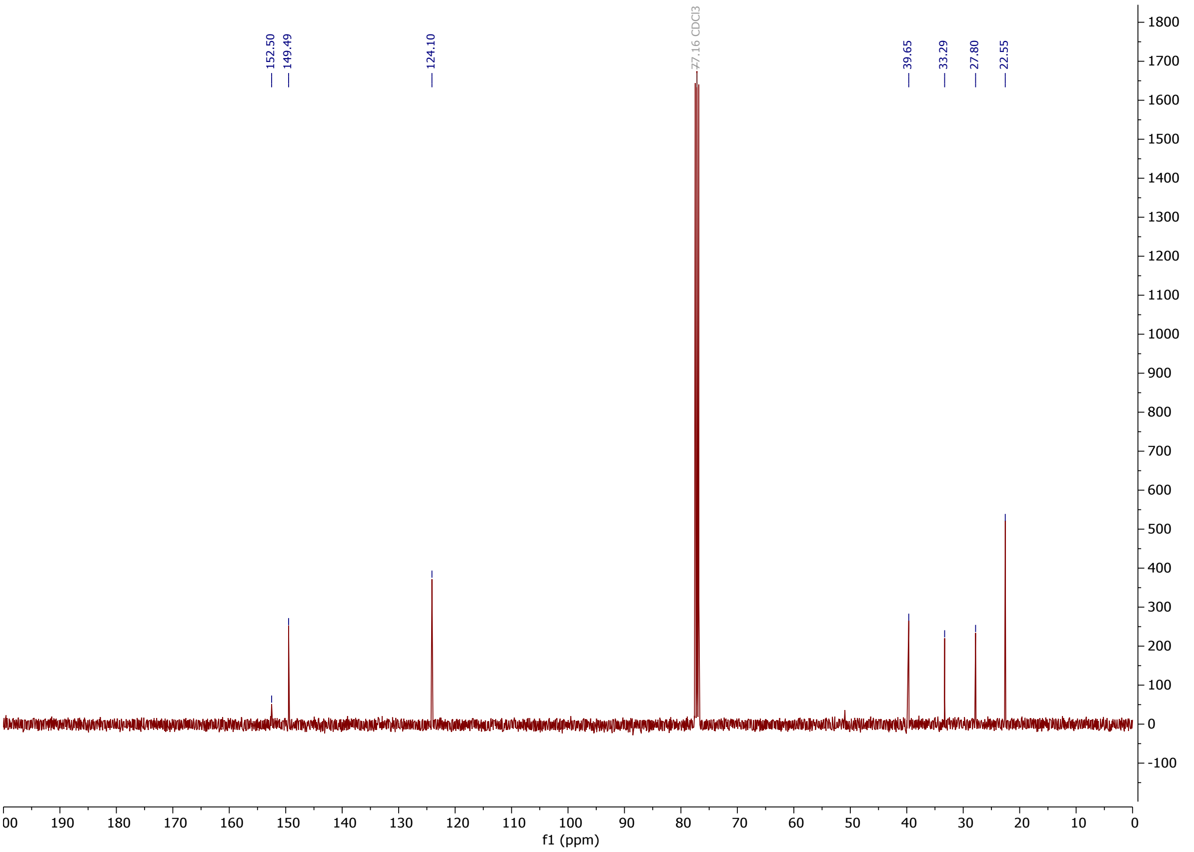

^{1}H NMR (400 MHz, $CDCl_3$) spectrum of **3s**

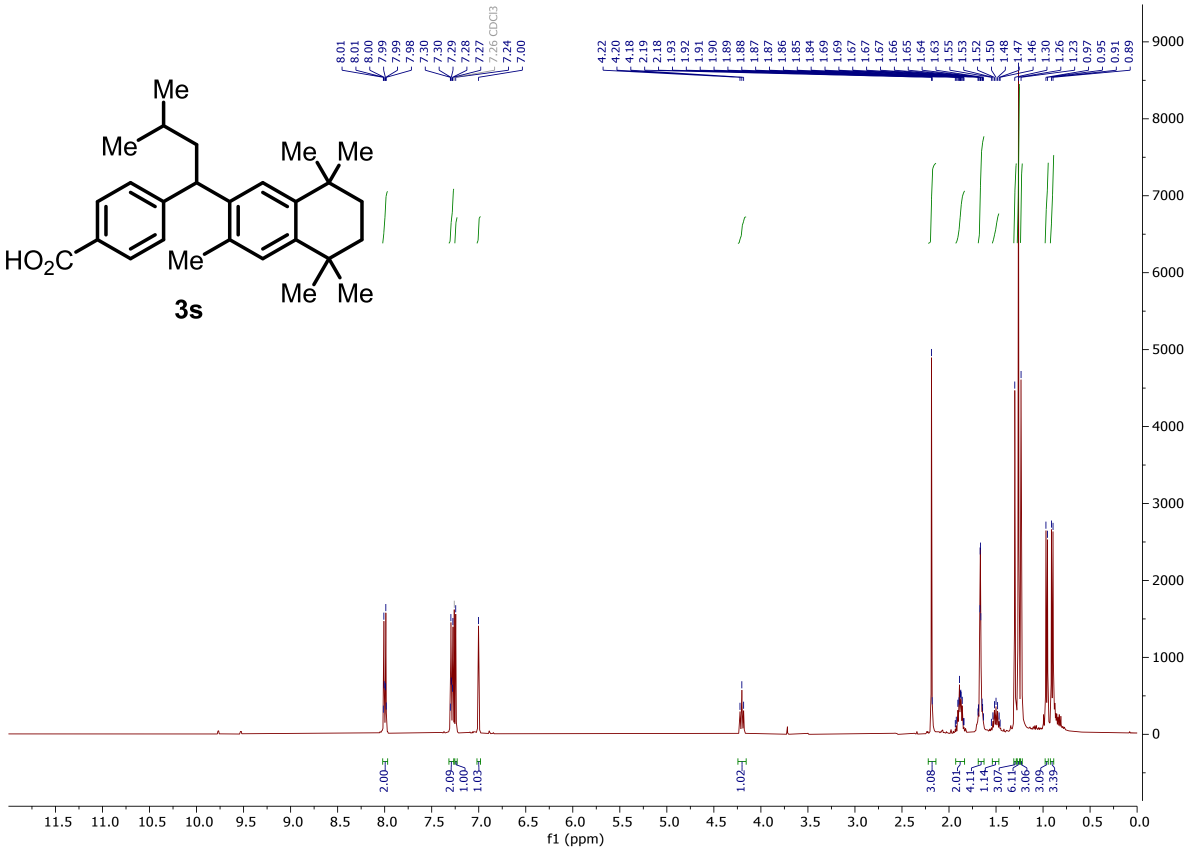

^{13}C{^{1}H} NMR (101 MHz, $CDCl_3$) spectrum of **3s**

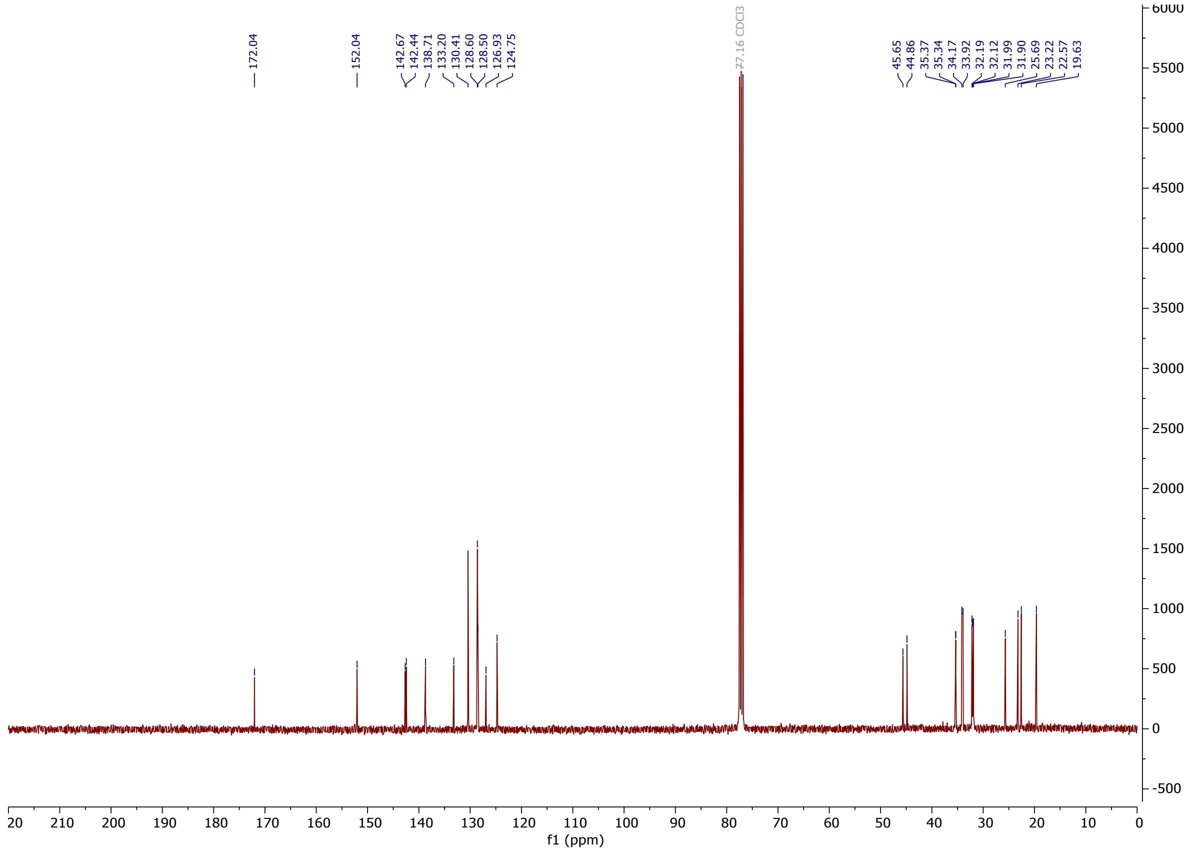

References

1. Artaryan, A.; Mardyukov, A.; Kulbitski, K.; Avigdori, I.; Nisnevich, G. A.; Schreiner, P. R.; Gandelman, M., Aliphatic C–H Bond Iodination by a N-Iodoamide and Isolation of an Elusive N-Amidyl Radical. *J. Org. Chem.* **2017,** *82* (14), 7093–7100.
2. Yi, J.; Lu, X.; Sun, Y.-Y.; Xiao, B.; Liu, L., Nickel-Catalyzed Sonogashira Reactions of Non-activated Secondary Alkyl Bromides and Iodides. *Angew. Chem. Int. Ed.* **2013,** *52* (47), 12409–12413.
3. Tissot, M.; Body, N.; Petit, S.; Claessens, J.; Genicot, C.; Pasau, P., Synthesis of Electron-Deficient Heteroaromatic 1,3-Substituted Cyclobutyls via Zinc Insertion/Negishi Coupling Sequence under Batch and Automated Flow Conditions. *Org. Lett.* **2018,** *20* (24), 8022–8025.
4. Rezazadeh, S.; Devannah, V.; Watson, D. A., Nickel-Catalyzed C-Alkylation of Nitroalkanes with Unactivated Alkyl Iodides. *J. Am. Chem. Soc.* **2017,** *139* (24), 8110–8113.
5. Lutter, F. H.; Grokenberger, L.; Benz, M.; Knochel, P., Cobalt-Catalyzed Csp3–Csp3 Cross-Coupling of Functionalized Alkylzinc Reagents with Alkyl Iodides. *Org. Lett.* **2020,** *22* (8), 3028–3032.
6. Miele, M.; Citarella, A.; Langer, T.; Urban, E.; Zehl, M.; Holzer, W.; Ielo, L.; Pace, V., Chemoselective Homologation–Deoxygenation Strategy Enabling the Direct Conversion of Carbonyls into (n+1)-Halomethyl-Alkanes. *Org. Lett.* **2020,** *22* (19), 7629–7634.
7. Hossain, M. A.; Sattenapally, N.; Parikh, H. I.; Li, W.; Rumbaugh, K. P.; German, N. A., Design, synthesis, and evaluation of compounds capable of reducing Pseudomonas aeruginosa virulence. *Eur. J. Med. Chem.* **2020,** *185*, 111800.
8. Zhang, G.; Bai, R.-X.; Li, C.-H.; Feng, C.-G.; Lin, G.-Q., Halogenation of 1,1-diarylethylenes by N-halosuccinimides. *Tetrahedron* **2019,** *75* (12), 1658–1662.
9. Molander, G. A.; Dowdy, E. D., Lanthanide-Catalyzed Hydroamination of Hindered Alkenes in Synthesis: Rapid Access to 10,11-Dihydro-5H-dibenzo- [a,d]cyclohepten-5,10-imines. *J. Org. Chem.* **1999,** *64* (17), 6515–6517.
10. Gu, F.; Huang, W.; Liu, X.; Chen, W.; Cheng, X., Substituted Hantzsch Esters as Versatile Radical Reservoirs in Photoredox Reactions. *Adv. Synth. Catal.* **2018,** *360* (5), 925–931.
11. Gao, L.; Wang, G.; Cao, J.; Yuan, D.; Xu, C.; Guo, X.; Li, S., Organocatalytic decarboxylative alkylation of N-hydroxy-phthalimide esters enabled by pyridine-boryl radicals. *Chem. Commun.* **2018,** *54* (82), 11534–11537.
12. Zhang, W.; Lin, S., Electroreductive Carbofunctionalization of Alkenes with Alkyl Bromides via a Radical-Polar Crossover Mechanism. *J. Am. Chem. Soc.* **2020,** *142* (49), 20661–20670.
13. Xie, H.; Guo, J.; Wang, Y.-Q.; Wang, K.; Guo, P.; Su, P.-F.; Wang, X.; Shu, X.-Z., Radical Dehydroxylative Alkylation of Tertiary Alcohols by Ti Catalysis. *J. Am. Chem. Soc.* **2020,** *142* (39), 16787–16794.
14. Suga, T.; Takahashi, Y.; Miki, C.; Ukaji, Y., Direct and Unified Access to Carbon Radicals from Aliphatic Alcohols by Cost-Efficient Titanium-Mediated Homolytic C−OH Bond Cleavage. *Angew. Chem. Int. Ed.* **2022,** *61* (10), e202112533.
15. He, B.; Pan, Q.; Guo, Y.; Chen, Q.-Y.; Liu, C., Cobalt-Catalyzed Radical Hydrotrifluoroethylation of Styrenes with Trifluoroethyl Iodide. *Org. Lett.* **2020,** *22* (16), 6552–6556.
16. Peng, L.; Li, Z.; Yin, G., Photochemical Nickel-Catalyzed Reductive Migratory Cross-Coupling of Alkyl Bromides with Aryl Bromides. *Org. Lett.* **2018,** *20* (7), 1880–1883.
17. Song, L.; Fu, D.-M.; Chen, L.; Jiang, Y.-X.; Ye, J.-H.; Zhu, L.; Lan, Y.; Fu, Q.; Yu, D.-G., Visible-Light Photoredox-Catalyzed Remote Difunctionalizing Carboxylation of Unactivated Alkenes with CO2. *Angew. Chem. Int. Ed.* **2020,** *59* (47), 21121–21128.
18. Fukuyama, T.; Nishikawa, T.; Yamada, K.; Ravelli, D.; Fagnoni, M.; Ryu, I., Photocatalyzed Site-Selective C(sp3)–H Functionalization of Alkylpyridines at Non-Benzylic Positions. *Org. Lett.* **2017,** *19* (23), 6436–6439.
19. Lowry, M. S.; Jonas I. Goldsmith; Jason D. Slinker; Richard Rohl; Pascal, J., Robert A.; George G. Malliaras; Bernhard, S., Single-Layer Electroluminescent Devices and Photoinduced Hydrogen Production from an Ionic Iridium(III) Complex. *Chem. Mater.* **2005,** *17* (23), 5712–5719.
